# Multimetallic catalysed radical oxidative C(sp^3^)–H/C(sp)–H cross-coupling between unactivated alkanes and terminal alkynes

**DOI:** 10.1038/ncomms11676

**Published:** 2016-06-24

**Authors:** Shan Tang, Pan Wang, Haoran Li, Aiwen Lei

**Affiliations:** 1 College of Chemistry and Molecular Sciences, Institute for Advanced Studies (IAS), Wuhan University, Wuhan 430072, China; 2 National Research Center for Carbohydrate Synthesis, Jiangxi Normal University, Nanchang 330022, China

## Abstract

Radical involved transformations are now considered as extremely important processes in modern organic synthetic chemistry. According to the demand by atom-economic and sustainable chemistry, direct C(sp^3^)–H functionalization through radical oxidative coupling represents an appealing strategy for C–C bond formations. However, the selectivity control of reactive radical intermediates is still a great challenge in these transformations. Here we show a selective radical oxidative C(sp^3^)–H/C(sp)–H cross-coupling of unactivated alkanes with terminal alkynes by using a combined Cu/Ni/Ag catalytic system. It provides a new way to access substituted alkynes from readily available materials. Preliminary mechanistic studies suggest that this reaction proceeds through a radical process and the C(sp^3^)–H bond cleavage is the rate-limiting step. This study may have significant implications for controlling selective C–C bond formation of reactive radical intermediates by using multimetallic catalytic systems.

## Abstract (web summary)

Cross coupling via the activation of two carbon-hydrogen bonds avoids the need for organometallic coupling partners and associated waste. Here, the authors report a radical process that allows selective coupling between alkynes and non-activated C(sp^3^)-H groups via a multimetallic catalytic system.

## Graphical abstract

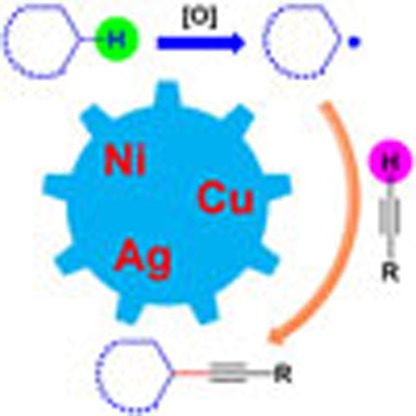

Substituted alkynes are fundamental structural motifs in numerous natural products, bioactive molecules and functional materials^1^,^2^,^3^. They also serve as versatile intermediates in many chemical transformations. Continuous efforts have been devoted to their synthesis throughout the history of organic chemistry. During the last decades, the transition-metal-catalysed Sonogashira coupling has been proven to be one of the most popular and efficient approach for the synthesis of substituted alkynes^4^. While early studies focused on C(sp^2^)–C(sp) coupling of terminal alkynes with vinyl/aryl electrophiles, recent attention has been paid to the C(sp^3^)–C(sp) coupling of terminal alkynes with unactivated alkyl halides. In 2003, Eckhardt and Fu^5^ pioneered the cross-coupling of terminal alkynes with unactivated primary bromides and iodides by using Pd/Cu synergistic catalysis with N–heterocyclic carbene ligands. Later on, this reaction protocol was extended to unactivated secondary bromides and iodides by Altenhoff *et al*.^6^. In 2009, Vechorkin *et al*.^7^ applied a combined Ni/Cu catalytic system to achieve the coupling of terminal alkynes with unactivated primary halides. Similarly, a modified Ni/Cu co-catalysed system was developed by Yi *et al*.^8^ to deal with the coupling with secondary bromides and iodides. More recently, Chen *et al*.^9^ developed a photo–promoted, transition-metal-free protocol to couple terminal alkynes with all types of unactivated alkyl iodides. As an alternative process for achieving the direct alkynylation of terminal alkynes, our group demonstrated a Pd-catalysed oxidative cross-coupling between terminal alkynes and alkylzinc reagents for the synthesis of substituted alkynes. Echoing the pursuit of atom-economic and sustainable chemistry, direct utilization of unactivated alkanes to replace unactivated alkyl halides and alkylzinc reagents in the synthesis of substituted alkynes has great significance in terms of both concept innovation and practical application.

Direct C(sp^3^)–H functionalization is a highly attractive approach for converting alkanes into functionalized organic compounds. However, the development of direct and selective methods for alkane functionalization is still in its infancy due to the low reactivity of C(sp^3^)–H bonds^10^,^11^,^12^,^13^,^14^,^15^. With the rapid development of C–H functionalization, direct C–H alkynylation with terminal alkynes has recently emerged as one of the most attractive approaches to access substituted alkynes^16^,^17^,^18^,^19^,^20^,^21^,^22^,^23^,^24^,^25^,^26^. This transformation has been considered to be challenging because of the facile homo-coupling and polymerization of terminal alkynes under oxidative conditions. Methods for the oxidative C(sp^3^)–H alkynylation of tertiary amines^27^,^28^,^29^,^30^ or benzylic ethers^31^,^32^ have been developed through normal cross-dehydrogenative coupling^33^ pathway (Fig. 1a), but direct oxidative C(sp^3^)–H alkynylation of unactivated alkanes with terminal alkynes to form substituted alkynes is still a great challenge and remains undeveloped.

Herein, we report a Cu/Ni/Ag co-catalysed oxidative C(sp^3^)–H alkynylation of unactivated alkanes with terminal alkynes. This protocol provides a new approach for the synthesis of substituted alkynes from readily available materials. Various alkanes and terminal alkynes are suitable in this transformation, affording the C(sp^3^)–C(sp) coupling product in good to high yields.

## Results

### Designing strategy

**Figure 1:**
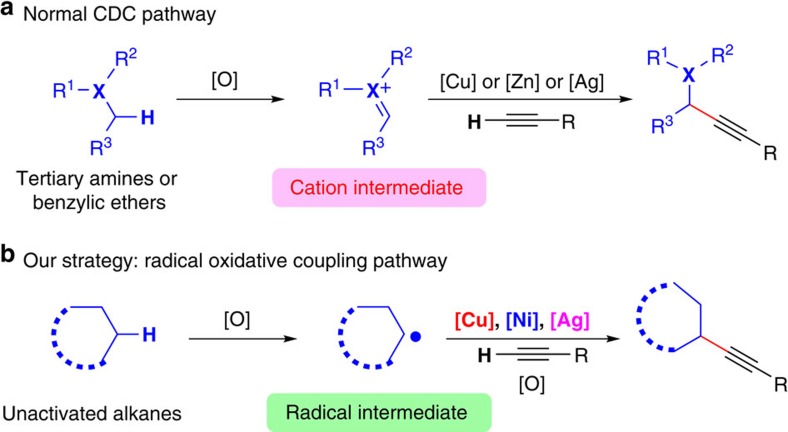
Approaches for C(sp^3^)–H/C(sp)–H coupling. (**a**) Alkynylation of activated C(sp^3^)–H bonds through CDC pathway. (**b**) Proposed radical oxidative coupling pathway for C(sp^3^)–H alkynylation of unactivated alkanes with terminal alkynes.

Radicals have been widely utilized in a large range of processes such as organic synthesis, biological processes and polymerization^34^,^35^,^36^,^37^. Generally, radicals with a single electron have a strong tendency to form chemical bonds. However, selective bond formation from radical intermediates was less developed compared with the ionic intermediates. Recent achievements showed that radical cross-coupling can provide a new opportunity for the formation of C–C bonds^38^,^39^,^40^. Considering that unactivated alkanes can be converted into corresponding alkyl radicals in the presence of oxidants, we envisioned that a radical oxidative cross-coupling pathway might provide a solution for the C(sp^3^)–H/C(sp)–H cross-coupling between unactivated alkanes and terminal alkynes (Fig. 1b). Nevertheless, the direct coupling of an alkyl radical with terminal alkynes usually ends up with reductive addition or difunctionalization to afford internal alkenes^41^,^42^,^43^,^44^. It was difficult to control the selectivity toward direct alkynylation rather than simple addition to alkyne. To deal with this challenging transformation, we wish to report a selective radical oxidative C(sp^3^)–H/C(sp)–H cross-coupling of unactivated alkanes with terminal alkynes by using a multimetallic catalysis^45^ system (Fig. 1b).

### Optimization of reaction conditions

**Figure 2:**
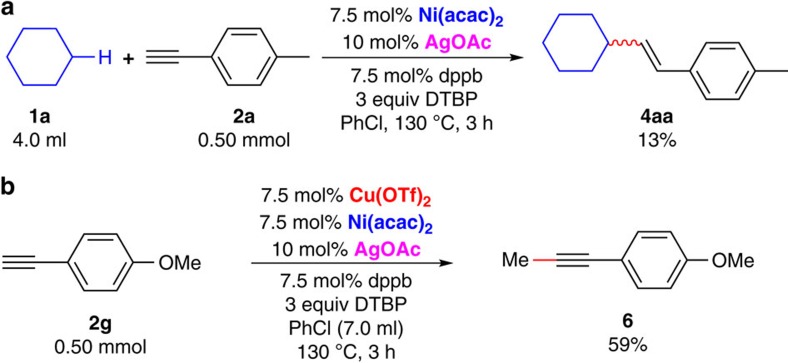
Control experiments. (**a**) Addition to terminal alkyne in the absence of copper catalyst. (**b**) Direct methylation of terminal alkyne in the absence of alkane substrates.

We started our research by examining the model reaction between cyclohexane (**1a**) and *p*-tolylacetylene (**2a**) under various conditions. After considerable efforts, we found that the combination of Cu(OTf)_2_, Ni(acac)_2_ and AgOAc as catalysts, 1,4–bis(diphenylphosphino)butane (dppb) as ligand, and di-*tert*-butyl peroxide (DTBP) as oxidant in chlorobenzene at 130 °C gave the best result (see Supplementary Tables 1–5 for detailed condition optimization). A 75% GC yield could be obtained within 3 h (Table 1, entry 1). The effect of each reaction parameter was examined and listed in Table 1. Both copper and nickel catalysts were crucial for this C(sp^3^)–C(sp) coupling reaction. Only 6% yield of the desired product was obtained in the absence of Ni(acac)_2_ (Table 1, entry 2). No desired products could be observed in the absence of Cu(OTf)_2_ (Table 1, entry 3). Instead, direct addition product **4a** was obtained in 13% yield (Fig. 2a). Moreover, CuOTf failed to furnish the coupling product (Table 1, entry 4). Ligand was not indispensable for this oxidative cross-coupling reaction. A moderate yield could be obtained in the absence of ligand (Table 1, entry 5). Addition of bipyridine did not improve the reaction yield (Table 1, entry 6). PPh_3_ was less effective than dppb in this transformation (Table 1, entry 7). Control experiments regarding the role of silver were also performed (Table 1, entries 8–9). In the absence of AgOAc, a good but slightly decreased yield could still be obtained (Table 1, entry 8). When CsOAc was used instead of AgOAc, the reaction resulted in a poor yield (Table 1, entry 9). Silver likely plays a role in the C(sp)–H activation step since it could coordinate with the alkynyl group^46^,^47^,^48^. Solvent effects were also investigated in this transformation. Without additional solvent, the reaction yield decreased significantly (Table 1, entry 10). Benzene gave a similar result with chlorobenzene (Table 1, entry 11). Different oxidants were also applied in this transformation. Dicumyl peroxide could furnish the desired product in a lower yield (Table 1, entry 12). Benzoyl peroxide was not suitable in this transformation (Table 1, entry 13). In addition, the influence of temperature was also explored. Both decreased and increased temperatures gave decreased yields (Table 1, entries 14–15). It is worthy of note that oligomerization of **2a** was the major side reaction pathway in all the above conditions.

### Scope of unactivated alkanes

**Figure 3:**
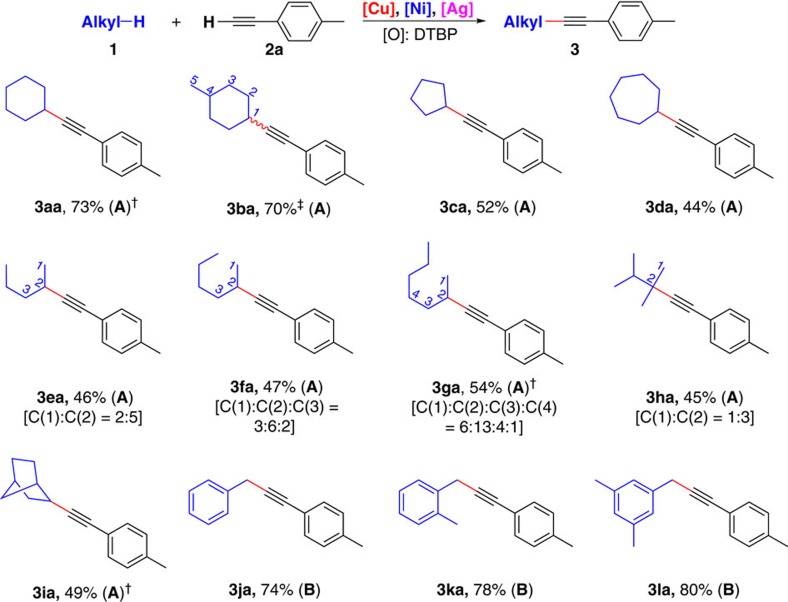
Scope of alkanes substrates. Reaction conditions **A:** **1** (4.0 ml), **2a** (0.50 mmol), Cu(OTf)_2_ (7.5 mol%), Ni(acac)_2_ (7.5 mol%), AgOAc (10 mol%), dppb (7.5 mol%) and DTBP (2.0 mmol), PhCl (3.0 ml), 130 °C, 3 h. The ratio of regioisomers shown in parentheses was determined by GC–MS. Reaction conditions **B:** **1** (7.0 ml), **2a** (0.50 mmol), Cu(OTf)_2_ (10 mol%), Ni(acac)_2_ (10 mol%), AgOAc (5 mol%), dppb (10 mol%) and DTBP (0.75 mmol), 130 °C, 3 h. ^†^DTBP (1.5 mmol) was used. ^‡^Yields of all regioisomers.

To further demonstrate the applicability of this transformation, the reaction system was applied to other unactivated alkanes for the synthesis of substituted alkynes (Fig. 3). Both cyclohexane and methylcyclohexane showed a good reaction efficiency toward alkynylation (**3aa** and **3ba**). Other cycloalkanes were applied as substrates in this transformation. The ring size had evident effect on the yield of the corresponding alkynylation products. For example, cyclopentane and cycloheptane did furnish the desired products but with decreased reaction efficiency (**3ca** and **3da**). Unactivated acyclic alkanes including linear alkanes and branched alkanes were tested in this oxidative C(sp^3^)–H alkynylation reaction. The reaction of **2a** with linear alkanes including *n*-pentane, *n*-hexane and *n*-heptane proceeded smoothly but afforded a mixture of regioisomers (**3ea**–**3ga**). The reaction result of a branched alkane was also presented. Neohexane was also able to couple with **2a** and afforded the desired product as two regioisomers (**3ha**). Oxidative C(sp^3^)–H alkynylation of norbornane proceeded with single-site selectivity and gave **3ia** in 49% yield. Despite simple alkanes, toluene derivatives were also suitable in this transformation. Direct oxidative benzylic C(sp^3^)–H alkynylation of toluene derivatives could be obtained in good yields under similar conditions (**3ja–3la**). Since *tert*-butoxyl radical can undergo β–Me scission to generate a methyl radical^49^,^50^,^51^,^52^, direct methylation of terminal alkyne was observed as a competing side reaction in the above cases. Importantly, efficient methylation of terminal alkyne could be achieved in the absence of alkane substrates (Fig. 2b).

### Scope of terminal alkynes

**Figure 4:**
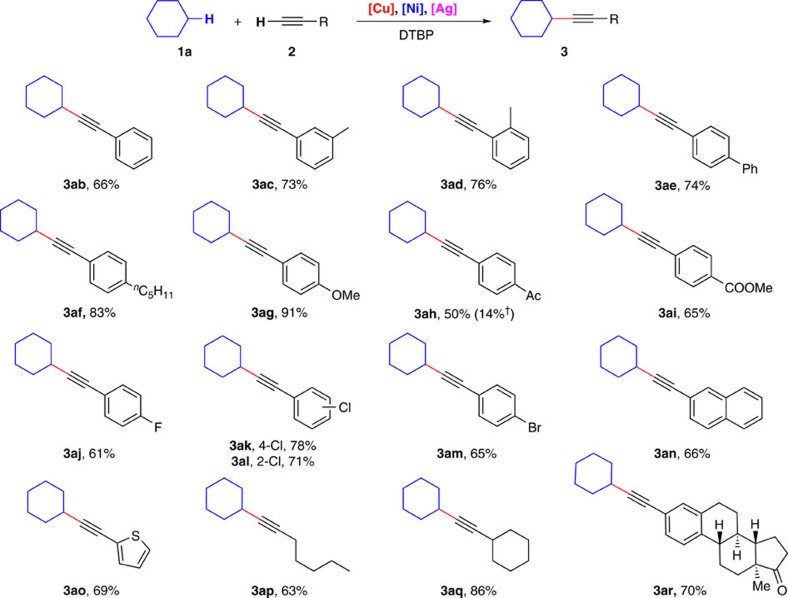
Scope of terminal alkynes. Reaction conditions: **1a** (4.0 ml), **2** (0.50 mmol), Cu(OTf)_2_ (7.5 mol%), Ni(acac)_2_ (7.5 mol%), dppb (7.5 mol%), AgOAc (10 mol%) and DTBP (1.5 mmol), PhCl (3.0 ml), 130 °C, 3 h. Isolated yields are shown. ^†^In the absence of AgOAc.

Different terminal alkynes were applied as substrates to react with cyclohexane (Fig. 4). The reactions of simple phenylacetylene, *meta*- and *ortho*-methyl substituted phenylacetylene all proceeded well and afforded the corresponding aliphatic internal alkynes in good yields (**3ab** and **3ad**). 4–Ethynyl–1,1′–biphenyl also gave the desired products in good yields (**3ae**). To our delight, electron-rich phenylacetylenes were more reactive, furnishing the desired products in higher yields (**3af** and **3ag**). At the same time, strongly electron-deficient phenylacetylenes were also suitable but with slightly decreased efficiency in this reaction system (**3ah** and **3ai**). It is noteworthy that silver had evident effect on the reaction yield of electron-deficient phenylacetylenes. For example, the yield of **3ah** decreased markedly (14%) in the absence of AgOAc. Notably, halide substituents such as F, Cl and Br were all tolerated in this transformation, which provides the possibility for further functionalization (**3aj**–**3am**). Other aromatic alkynes were also applied in this transformation. 2-ethynylnaphthalene and 2-ethynylthiophene both furnished the desired products in good yields (**3an** and **3ao**). Delightfully, aliphatic alkyne such as 1-heptyne and cyclohexylacetylene were also suitable in the oxidative C(sp^3^)–H/C(sp) cross-coupling and afforded the desired product in good to excellent yields (**3ap** and **3aq**). Late-stage modification of a bioactive molecule is highly important for medical chemistry studies. Delightfully, 3-ethynylestrone containing carbonyl group and four continuous chiral centres furnished the desired coupling product in 70% yield under the standard conditions (**3ar**).

## Discussion

**Figure 5:**
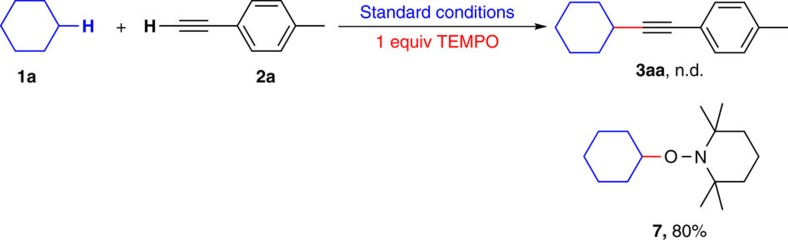
Radical trapping experiment. Observation of trapped cyclohexyl radical.

Since the method had been established, we then tried to gain some insights into the catalytic pathway. To confirm the existence of radical intermediates, a radical trapping experiment was carried out by using 1 equiv of (2, 2, 6, 6–tetramethylpiperidin–1–yl)oxy (TEMPO). No cross-coupling product **3aa** was obtained in this reaction (Fig. 5). Instead, the GC–MS and ^1^H NMR analysis of the reaction mixture showed the existence of cyclohexyl radical trapped by TEMPO (Supplementary Figs 66,67).

**Figure 6:**
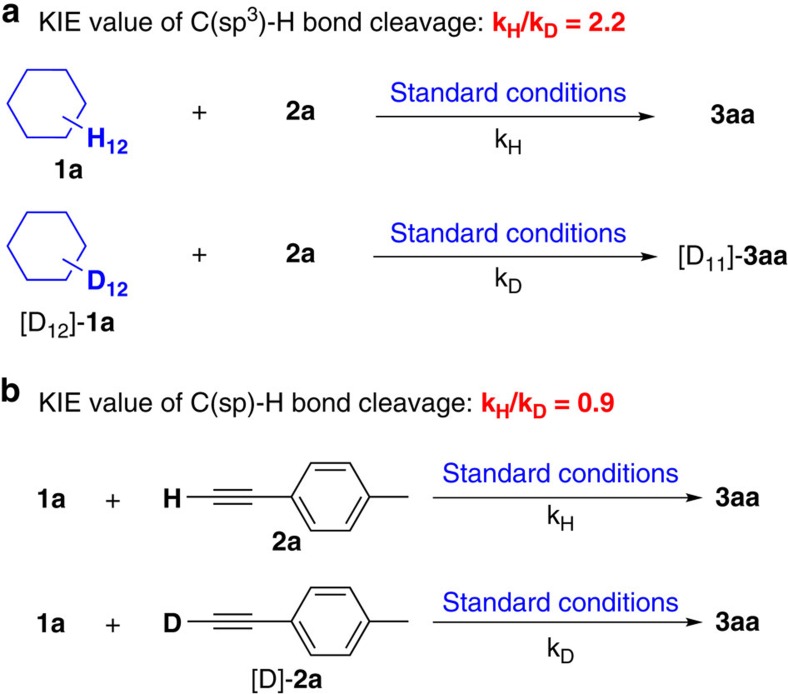
Kinetic isotope effect experiments. (**a**) The initial reaction rate of **1a** with **2a**, [D_12_]–**1a** with **2a**. (**b**) The initial reaction rate of **1a** with **2a**, **1a** with [D]–**2a**.

Next, kinetic isotopic effect studies with separate kinetic experiments were performed to gain insights into the rate-determining step for this C–H/C–H cross-coupling reaction. Both the C(sp^3^)–H bond cleavage of **1a** and the C(sp)–H bond cleavage of **2a** were studied. A primary kinetic isotopic effect was observed for C(sp^3^)–H bond cleavage (Fig. 6a, k_H_/k_D_=2.2) while no obvious kinetic isotopic effect was observed for the C(sp)–H cleavage (Fig. 6b, k_H_/k_D_=0.9), suggesting that C(sp^3^)–H bond cleavage was probably the rate-determining step in this transformation (for details, see Supplementary Fig. 68)^53^.

**Figure 7:**
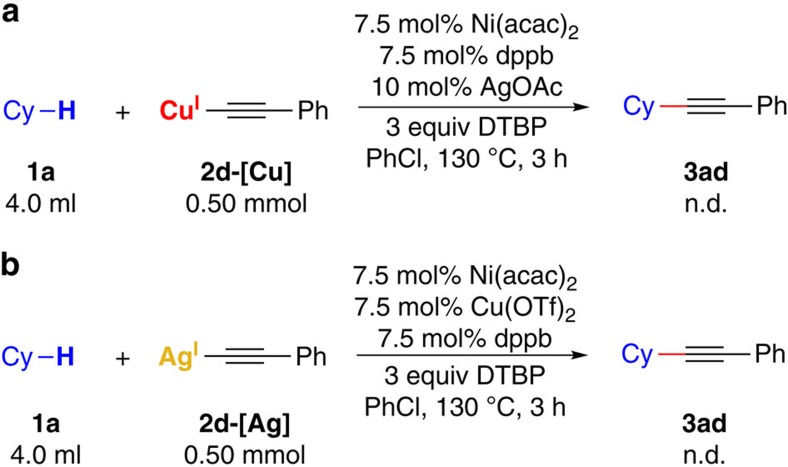
Reactions of cyclohexane with alkynyl metal species. (**a**) Reaction between **1a** and **2d–[Cu]** under standard conditions. (**b**) Reaction between **1a** and **2d–[Ag]** under standard conditions.

In the next step, the reactions with alkynyl metal species were performed to get some insights into the radical cross-coupling step. (Phenylethynyl)copper (2d–[Cu]) and (phenylethynyl)silver (2d–[Ag]) were prepared and used as substrates to react with **1a** under the standard conditions (Fig. 7). However, neither of them could furnish the cross-coupling product. Thus, both alkynyl Cu(I) complex and alkynyl Ag(I) complex are not likely to be involved in the C(sp^3^)–C(sp) cross-coupling process. An alkynyl Cu(II) complex is more possibly to be generated in this transformation.

**Figure 8:**
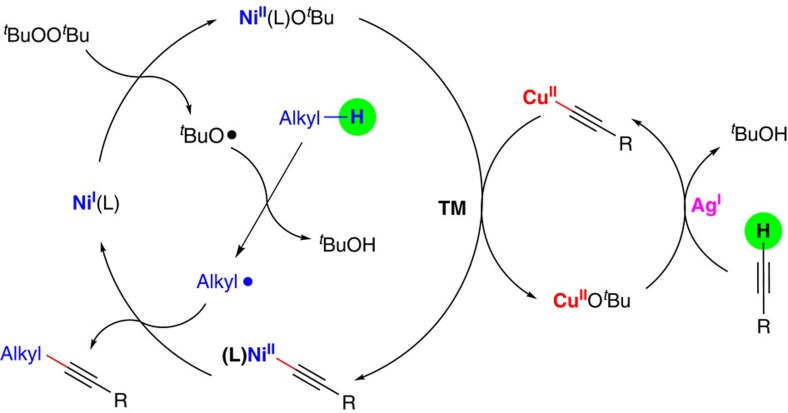
Proposed catalytic cycles for the radical alkynylation reaction through multimetallic catalysis. Tentative reaction mechanism involves oxidation of alkane to generate an alkyl radical, silver-assisted copper(II)-acetylide formation, transmetalation with nickel, and finally C(sp^3^)–C(sp) bond formation.

On the basis of the experimental results and previous reports^50^,^51^, a plausible reaction mechanism is presented in Fig. 8. Copper and silver work synergistically in the C(sp)–H activation of terminal alkyne, which leads to the formation of an alkynyl Cu(II) complex. The alkynyl copper complex is then transmetaled with Ni(II) species to generate an alkynyl Ni(II) complex. At the same time, an alkyl radical can be generated through hydrogen abstraction by *in situ* generated *tert*-butoxyl radical. This radical then reacts with the Ni(II) alkynyl complex^54^,^55^,^56^,^57^,^58^,^59^. The C(sp^3^)–C(sp) bond can be formed either through radical homolytic substitution or reductive elimination (see Supplementary Fig. 69 for details). Finally, the released Ni(I) species can be oxidized to Ni(II) species by DTBP to complete the nickel catalytic cycle.

In conclusion, we have developed a combined Cu/Ni/Ag catalytic system to achieve the challenging oxidative C(sp^3^)–H/C(sp)–H cross-coupling of unactivated alkanes with terminal alkynes. The utilization of multimetallic catalysis was the key for controlling the reaction selectivity toward C(sp^3^)–C(sp) bond formation. Various substituted alkynes were synthesized in good to high yields with a good functional group tolerance. Preliminary mechanistic studies suggest that the reaction proceeds through a transition-metal-catalysed radical reaction pathway and that the C(sp^3^)–H bond cleavage of unactivated alkanes is the rate-limiting step. This work not only provides an environmentally friendly approach to access alkyne compounds, but also contributes new knowledge to radical cross-coupling chemistry. The application of the radical alkynylation strategy in the synthesis of other substituted alkynes is underway in our laboratory.

## Methods

### General procedure (3aa)

In an oven-dried Teflon septum screw-capped tube equipped with a stir bar, Cu(OTf)_2_ (13.6 mg, 0.038 mmol), Ni(acac)_2_ (9.6 mg, 0.038 mmol), dppb (16.0 mg, 0.038 mmol) and AgOAc (8.3 mg, 0.050 mmol) were combined and sealed. The tube was then charged with nitrogen. Then cyclohexane (4.0 ml) and PhCl (3.0 ml) were injected into the tube by syringe. After stirring for 5 min, DTBP (135 mg, 1.5 mmol) and *p*-tolylacetylene (58.0 mg, 0.50 mmol) were subsequently injected into the reaction tube. The reaction was then heated to 130 °C. After stirring for 3 h, the reaction was cooled down to room temperature and quenched with saturated Na_2_S_2_O_3_ solution. After extraction with ethyl acetate (3 × 10 ml), the organic layers were combined and dried over anhydrous Na_2_SO_4_, the pure product was obtained by flash column chromatography on silica gel (petroleum:ethyl ether=10:1). Colourless oil was obtained in 73% isolated yield. ^1^H NMR (400 MHz, CDCl_3_) *δ* 7.28 (d, *J*=8.1 Hz, 2H), 7.06 (d, *J*=7.9 Hz, 2H), 2.56 (tt, *J*=9.0, 3.6 Hz, 1H), 2.31 (s, 3H), 1.92–1.82 (m, 2H), 1.80–1.69 (m, 2H), 1.59–1.46 (m, 3H), 1.40–1.28 (m, 3H). ^13^C NMR (101 MHz, CDCl_3_) *δ* 137.26, 131.38, 128.84, 120.99, 93.58, 80.47, 32.75, 29.65, 25.92, 24.90, 21.34. For ^1^H NMR, ^13^C NMR, ^19^F NMR and GC–MS (if applicable) spectra of compounds **3aa-3la**, **3ab–3ar**, **6** see Supplementary Figs 1–66. For the general information of the analytical methods and the mechanistic studies, please see Supplementary Methods.

### Data availability

The authors declare that the data supporting the findings of this study are available within the article and its Supplementary Information files.

## Additional information

**How to cite this article:** Tang, S. *et al*. Multimetallic catalysed radical oxidative C(sp^3^)–H/C(sp)–H cross-coupling between unactivated alkanes and terminal alkynes. *Nat. Commun.* 7:11676 doi: 10.1038/ncomms11676 (2016).

## Supplementary Material

Supplementary Information Supplementary Figures 1-69, Supplementary Tables 1-5, Supplementary Methods and Supplementary References

## Figures and Tables

**Table 1:**
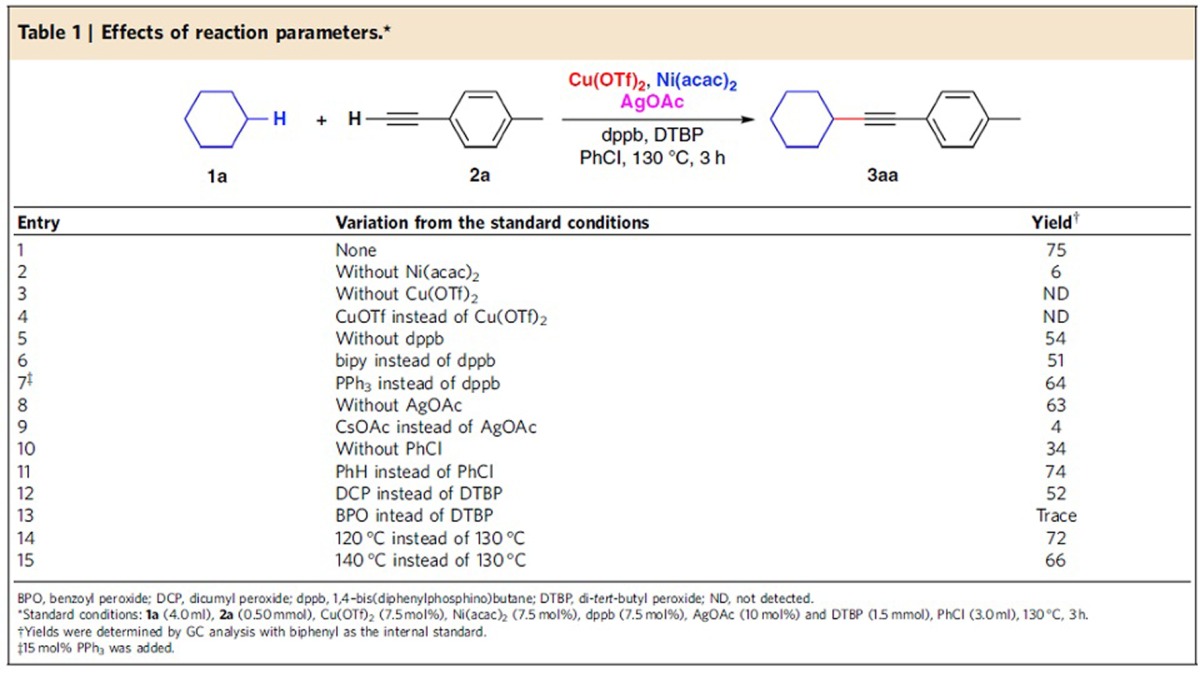
Effects of reaction parameters.

## References

[b1] Stang P. J. Diederich Fo. Modern acetylene chemistry VCH (1995).

[b2] Negishi E.–i. & Anastasia L. Palladium-catalysed alkynylation. Chem. Rev. 103, 1979–2018 (2003). 12744698 10.1021/cr020377i

[b3] Liu J., Lam J. W. Y. & Tang B. Z. Acetylenic polymers: syntheses, structures, and functions. Chem. Rev. 109, 5799–5867 (2009). 19678641 10.1021/cr900149d

[b4] Chinchilla R. & Nájera C. The sonogashira reaction: a booming methodology in synthetic organic chemistry. Chem. Rev. 107, 874–922 (2007). 17305399 10.1021/cr050992x

[b5] Eckhardt M. & Fu G. C. The first applications of carbene ligands in cross-couplings of alkyl electrophiles: Sonogashira reactions of unactivated alkyl bromides and iodides. J. Am. Chem. Soc. 125, 13642–13643 (2003). 14599185 10.1021/ja038177r

[b6] Altenhoff G., Würtz S. & Glorius F. The first palladium–catalysed Sonogashira coupling of unactivated secondary alkyl bromides. Tetrahedron. Lett. 47, 2925–2928 (2006).

[b7] Vechorkin O., Barmaz D., Proust V. & Hu X. Ni–catalysed Sonogashira coupling of nonactivated alkyl halides: orthogonal functionalization of alkyl iodides, bromides, and chlorides. J. Am. Chem. Soc. 131, 12078–12079 (2009). 19670863 10.1021/ja906040t

[b8] Yi J., Lu X., Sun Y.–Y., Xiao B. & Liu L. Nickel–catalysed sonogashira reactions of non–activated secondary alkyl bromides and iodides. Angew. Chem. Int. Ed. 52, 12409–12413 (2013). 10.1002/anie.201307069 24115611

[b9] Chen M., Zheng X., Li W., He J. & Lei A. Palladium–catalysed aerobic oxidative cross–coupling reactions of terminal alkynes with alkylzinc reagents. J. Am. Chem. Soc. 132, 4101–4103 (2010). 20218583 10.1021/ja100630p

[b10] Zhang S.–Y., Zhang F.–M. & Tu Y.–Q. Direct Sp^3^ [small alpha]–C–H activation and functionalization of alcohol and ether. Chem. Soc. Rev. 40, 1937–1949 (2011). 21286642 10.1039/c0cs00063a

[b11] Girard S. A., Knauber T. & Li C.–J. The cross-dehydrogenative coupling of C(sp)^3^–H Bonds: a versatile strategy for C–C bond formations. Angew Chem. Int. Ed. 53, 74–100 (2014). 10.1002/anie.201304268 24214829

[b12] Chen H., Schlecht S., Semple T. C. & Hartwig J. F. Thermal, catalytic, regiospecific functionalization of alkanes. Science 287, 1995–1997 (2000). 10720320 10.1126/science.287.5460.1995

[b13] White M. C. Adding aliphatic C–H bond oxidations to synthesis. Science 335, 807–809 (2012). 22344434 10.1126/science.1207661

[b14] Antonchick A. P. & Burgmann L. Direct selective oxidative cross–coupling of simple alkanes with heteroarenes. Angew Chem. Int. Ed. 52, 3267–3271 (2013). 10.1002/anie.201209584 23364911

[b15] Li K., Wu Q., Lan J. & You J. Coordinating activation strategy for C(sp^3^)-H/C(sp^3^)-H cross-coupling to access [beta]-aromatic [alpha]-amino acids. Nat. Commun. 6, 8404 (2015). 26415985 10.1038/ncomms9404 PMC4598627

[b16] Haro T. d. & Nevado C. Gold–catalysed ethynylation of arenes. J. Am. Chem. Soc. 132, 1512–1513 (2010). 20088525 10.1021/ja909726h

[b17] Wei Y., Zhao H., Kan J., Su W. & Hong M. Copper–catalysed direct alkynylation of electron–deficient polyfluoroarenes with terminal alkynes using O_2_ as an oxidant. J. Am. Chem. Soc. 132, 2522–2523 (2010). 20131777 10.1021/ja910461e

[b18] Yang L., Zhao L. & Li C.–J. Palladium–catalysed direct oxidative Heck–Cassar–Sonogashira type alkynylation of indoles with alkynes under oxygen. Chem. Commun. 46, 4184–4186 (2010). 10.1039/c0cc00014k 20440430

[b19] Matsuyama N., Kitahara M., Hirano K., Satoh T. & Miura M. Nickel– and Copper–catalysed direct alkynylation of azoles and polyfluoroarenes with terminal alkynes under O_2_ or atmospheric conditions. Org. Lett. 12, 2358–2361 (2010). 20415435 10.1021/ol100699g

[b20] Patil S. S., Jadhav R. P., Patil S. V. & Bobade V. D. Ligand and solvent–free iron catalysed oxidative alkynylation of azoles with terminal alkynes. Tetrahedron. Lett. 52, 5617–5619 (2011).

[b21] Kim S. H., Park S. H. & Chang S. Palladium-catalysed oxidative alkynylation of arene C–H bond using the chelation-assisted strategy. Tetrahedron 68, 5162–5166 (2012).

[b22] Shibahara F., Dohke Y. & Murai T. Palladium-catalysed C–H bond direct alkynylation of 5–membered heteroarenes: a well–defined synthetic route to azole derivatives containing two different alkynyl groups. J. Org. Chem. 77, 5381–5388 (2012). 22612569 10.1021/jo3008385

[b23] Jie X., Shang Y., Hu P. & Su W. Palladium-catalysed oxidative cross–coupling between heterocycles and terminal alkynes with low catalyst loading. Angew Chem. Int. Ed. 52, 3630–3633 (2013). 10.1002/anie.201210013 23404782

[b24] Shang M., Wang H.–L., Sun S.–Z., Dai H.–X. & Yu J.–Q. Cu(II)–mediated ortho C–H alkynylation of (hetero)arenes with terminal alkynes. J. Am. Chem. Soc. 136, 11590–11593 (2014). 25087720 10.1021/ja507704b

[b25] Liu Y.–H., Liu Y.–J., Yan S.–Y. & Shi B.–F. Ni(II)–catalysed dehydrative alkynylation of unactivated (hetero)aryl C–H bonds using oxygen: a user-friendly approach. Chem. Commun. 51, 11650–11653 (2015). 10.1039/c5cc03729h 26099578

[b26] Liu Y.–J., Liu Y.–H., Yin X.–S., Gu W.–J. & Shi B.–F. Copper/Silver-mediated direct ortho–ethynylation of unactivated (hetero)aryl C–H Bonds with terminal alkyne. Chem. Eur. J. 21, 205–209 (2015). 25400131 10.1002/chem.201405594

[b27] Li Z. & Li C.–J. CuBr–catalysed efficient alkynylation of sp^3^ C−H bonds adjacent to a nitrogen atom. J. Am. Chem. Soc. 126, 11810–11811 (2004). 15382913 10.1021/ja0460763

[b28] Volla C. M. R., Vogel P. & Chemoselective C−H bond activation: Ligand and solvent free iron–catalysed oxidative C−C cross–coupling of tertiary amines with terminal alkynes. Reaction scope and mechanism. Org. Lett. 11, 1701–1704 (2009). 19296636 10.1021/ol9002509

[b29] Xu X. & Li X. Copper/diethyl azodicarboxylate mediated regioselective alkynylation of unactivated aliphatic tertiary methylamine with terminal alkyne. Org. Lett. 11, 1027–1029 (2009). 19159324 10.1021/ol802974b

[b30] Sugiishi T. & Nakamura H. Zinc(II)–catalysed redox cross–dehydrogenative coupling of propargylic amines and terminal alkynes for synthesis of N–tethered 1,6–enynes. J. Am. Chem. Soc. 134, 2504–2507 (2012). 22283631 10.1021/ja211092q

[b31] Correia C. A. & Li C. J. Silver–catalysed oxidative coupling of terminal aromatic alkynes and benzylic ethers. Heterocycles 82, 555–562 (2010).

[b32] Xiang S.–K., Zhang B., Zhang L.–H., Cui Y. & Jiao N. Iron–mediated cross dehydrogenative coupling (CDC) of terminal alkynes with benzylic ethers and alkanes. Sci. China Chem. 55, 50–54 (2012).

[b33] Li C.–J. Cross–dehydrogenative Ccoupling (CDC): exploring C−C bond formations beyond functional group transformations. Acc. Chem. Res. 42, 335–344 (2009). 19220064 10.1021/ar800164n

[b34] Tōgō H. Advanced free radical reactions for organic synthesis 1st edn Elsevier (2004).

[b35] Jasperse C. P., Curran D. P. & Fevig T. L. Radical reactions in natural product synthesis. Chem. Rev. 91, 1237–1286 (1991).

[b36] Riley P. A. Free radicals in biology: oxidative stress and the effects of ionizing radiation. Int. J. Radiat. Biol. 65, 27–33 (1994). 7905906 10.1080/09553009414550041

[b37] Carraher C. E. Introduction to polymer chemistry 2nd edn CRC Press (2010).

[b38] Liu Q., Jackstell R. & Beller M. Oxidative catalytic coupling reactions: selective formation of C–C and C–X bonds using radical processes. Angew Chem. Int. Ed. 52, 13871–13873 (2013). 10.1002/anie.201307865 24194256

[b39] Liu C., Liu D. & Lei A. Recent advances of transition–metal catalysed radical oxidative cross–couplings. Acc. Chem. Res. 47, 3459–3470 (2014). 25364854 10.1021/ar5002044

[b40] Studer A. & Curran D. P. Catalysis of radical reactions: a radical chemistry perspective. Angew Chem. Int. Ed. 55, 58–102 (2015). 10.1002/anie.201505090 26459814

[b41] Ichinose Y., Matsunaga S.–I., Fugami K., Oshima K. & Utimoto K. Triethylborane-induced radical adsdition of alkyl iodides to acetylenes. Tetrahedron. Lett. 30, 3155–3158 (1989).

[b42] Liu Z.–Q. . Free-radical-initiated coupling reaction of alcohols and alkynes: Not C−O but C−C bond formation. Org. Lett. 11, 1437–1439 (2009). 19243113 10.1021/ol900145u

[b43] Cheung C. W., Zhurkin F. E. & Hu X. Z–Selective olefin synthesis via iron–catalysed reductive coupling of alkyl halides with terminal Arylalkynes. J. Am. Chem. Soc. 137, 4932–4935 (2015). 25831473 10.1021/jacs.5b01784 PMC4415033

[b44] Li J., Zhang J., Tan H. & Wang D. Z. Visible-light-promoted vinylation of tetrahydrofuran with alkynes through direct C–H bond functionalization. Org. Lett. 17, 2522–2525 (2015). 25908495 10.1021/acs.orglett.5b01053

[b45] Ackerman L. K. G., Lovell M. M. & Weix D. J. Multimetallic catalysed cross-coupling of aryl bromides with aryl triflates. Nature 524, 454–457 (2015). 26280337 10.1038/nature14676 PMC4552586

[b46] Létinois–Halbes U., Pale P. & Berger S. Ag NMR as a tool for mechanistic studies of Ag–catalysed reactions: Evidence for *in situ* formation of alkyn–1–yl silver from alkynes and silver salts. J. Org. Chem. 70, 9185–9190 (2005). 16268588 10.1021/jo0511546

[b47] Halbes–Letinois U., Weibel J.–M. & Pale P. The organic chemistry of silver acetylides. Chem. Soc. Rev. 36, 759–769 (2007). 17471400 10.1039/b602151b

[b48] Vitérisi A., Orsini A., Weibel J.–M. & Pale P. A mild access to silver acetylides from trimethylsilyl acetylenes. Tetrahedron. Lett. 47, 2779–2781 (2006).

[b49] Walling C. Free radicals in solution Wiley (1957).

[b50] Tran B. L., Driess M. & Hartwig J. F. Copper-catalysed oxidative dehydrogenative carboxylation of unactivated alkanes to allylic esters via alkenes. J. Am. Chem. Soc. 136, 17292–17301 (2014). 25389772 10.1021/ja510093x PMC4262675

[b51] Tran B. L., Li B., Driess M. & Hartwig J. F. Copper-catalysed intermolecular amidation and imidation of unactivated alkanes. J. Am. Chem. Soc. 136, 2555–2563 (2014). 24405209 10.1021/ja411912p PMC3985719

[b52] Bunescu A., Wang Q. & Zhu J. Synthesis of functionalized epoxides by copper-catalysed alkylative epoxidation of allylic alcohols with alkyl nitriles. Org. Lett. 17, 1890–1893 (2015). 25825802 10.1021/acs.orglett.5b00571

[b53] Simmons E. M. & Hartwig J. F. On the interpretation of deuterium kinetic isotope effects in C–H bond functionalizations by transition-metal complexes. Angew Chem. Int. Ed. 51, 3066–3072 (2012). 10.1002/anie.201107334 22392731

[b54] Jones G. D. . Ligand redox effects in the synthesis, electronic structure, and reactivity of an alkyl−alkyl cross–coupling catalyst. J. Am. Chem. Soc. 128, 13175–13183 (2006). 17017797 10.1021/ja063334i

[b55] Lin X. & Phillips D. L. Density functional theory studies of negishi alkyl–alkyl cross–coupling reactions catalysed by a methylterpyridyl–Ni(I) complex. J. Org. Chem. 73, 3680–3688 (2008). 18410144 10.1021/jo702497p

[b56] Lin X., Sun J., Xi Y. & Lin D. How racemic secondary alkyl electrophiles proceed to enantioselective products in Negishi cross–coupling reactions. Organometallics 30, 3284–3292 (2011).

[b57] Biswas S. & Weix D. J. Mechanism and selectivity in nickel-catalysed cross-electrophile coupling of aryl halides with alkyl halides. J. Am. Chem. Soc. 135, 16192–16197 (2013). 23952217 10.1021/ja407589e PMC3946589

[b58] Breitenfeld J., Ruiz J., Wodrich M. D. & Hu X. Bimetallic oxidative addition involving radical intermediates in nickel-catalysed alkyl–alkyl Kumada coupling reactions. J. Am. Chem. Soc. 135, 12004–12012 (2013). 23865460 10.1021/ja4051923

[b59] Schley N. D. & Fu G. C. Nickel-catalysed Negishi arylations of propargylic bromides: A mechanistic investigation. J. Am. Chem. Soc. 136, 16588–16593 (2014). 25402209 10.1021/ja508718m PMC4277758

