# Supplementary material for: Multimetallic catalysed radical oxidative C(sp3)–H/C(sp)–H cross-coupling between unactivated alkanes and terminal alkynes
Source: Nat Commun. 2016 Jun 24;7:11676. doi: 10.1038/ncomms11676 (PMC4931026; doi:10.1038/ncomms11676)
Supplement: Supplementary Information — Supplementary Figures 1-69, Supplementary Tables 1-5, Supplementary Methods and Supplementary References [file ncomms11676-s1.pdf]

Supplementary Figures

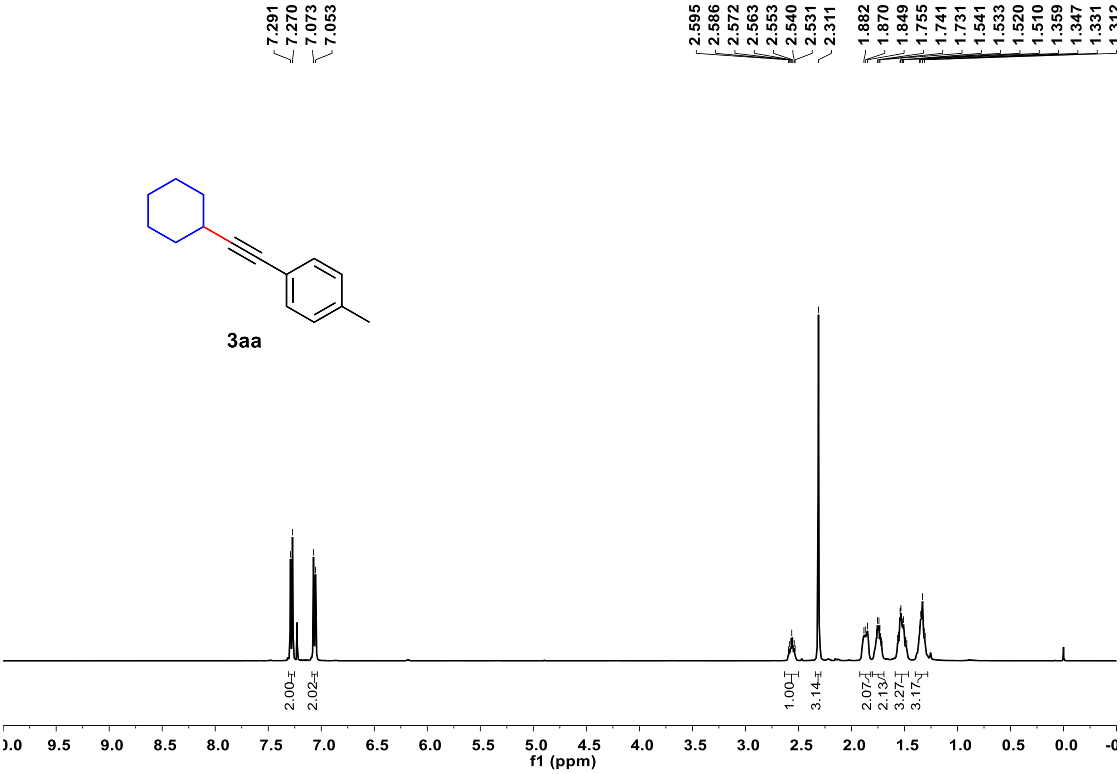

Supplementary Figure 1. ^{1}H NMR (400 MHz, $CDCl_3$) spectrum of 3aa

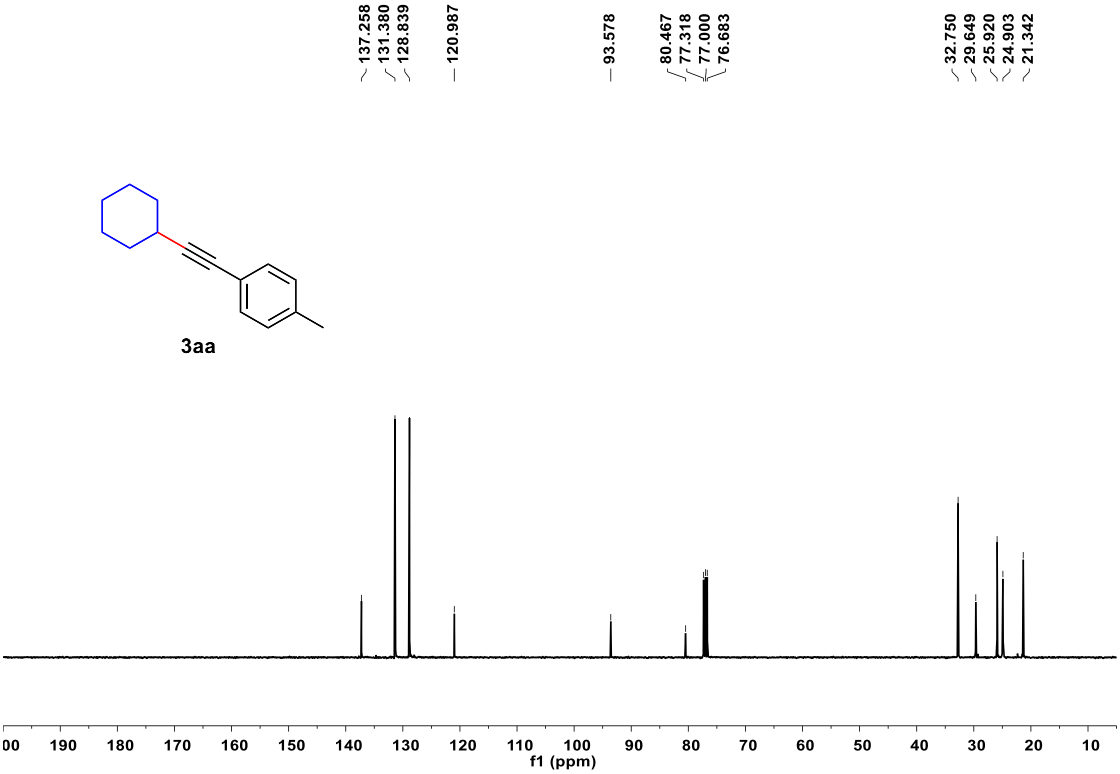

Supplementary Figure 2. ^{13}C NMR (101 MHz, $CDCl_3$) spectrum of 3aa

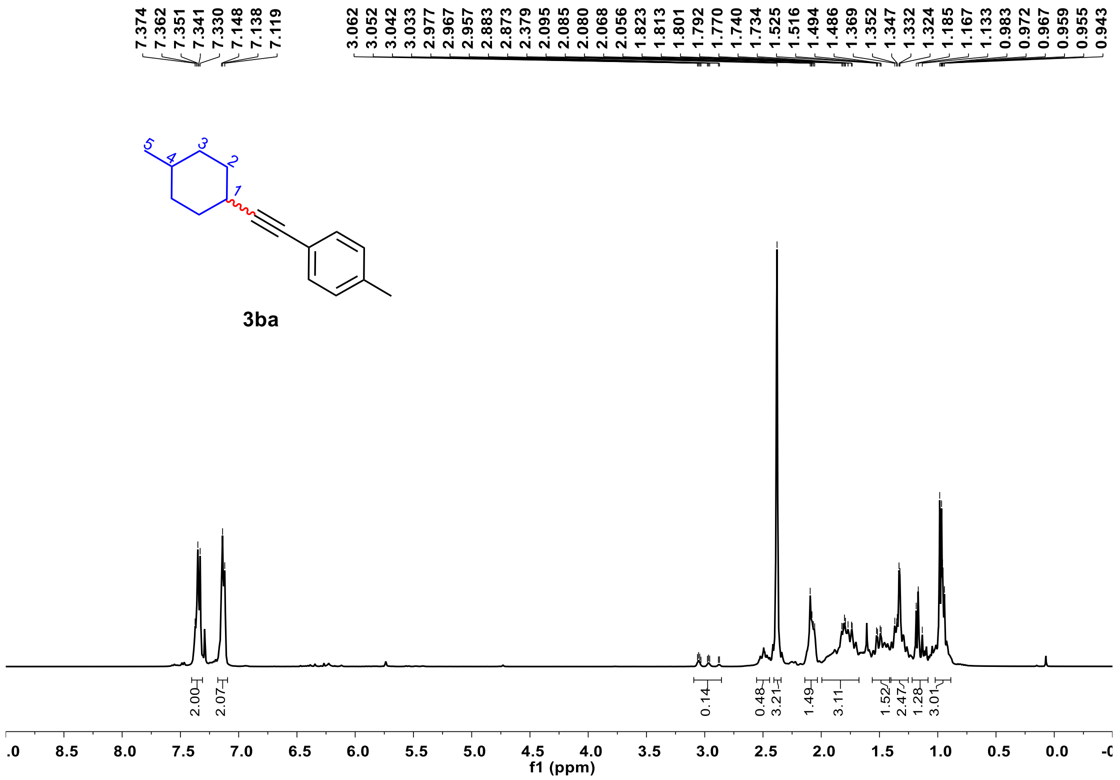

Supplementary Figure 3. ^{1}H NMR (400 MHz, $CDCl_3$) spectrum of 3ba.

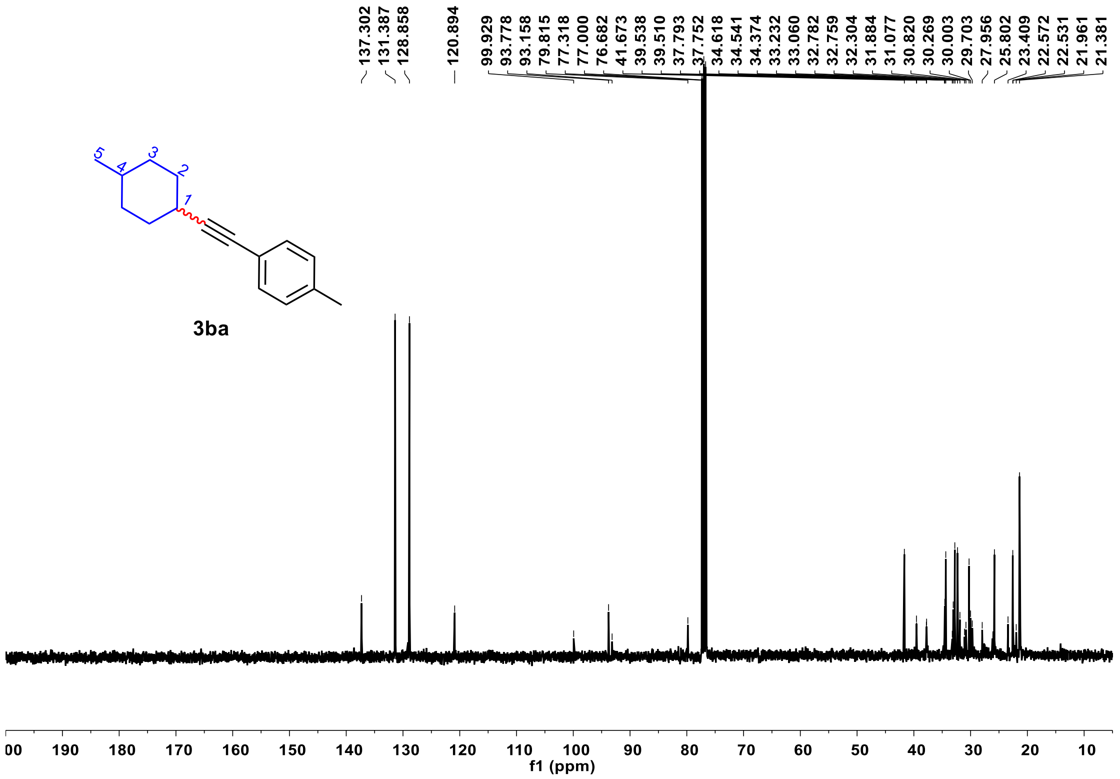

Supplementary Figure 4. ^{13}C NMR (101 MHz, $CDCl_3$) spectrum of 3ba

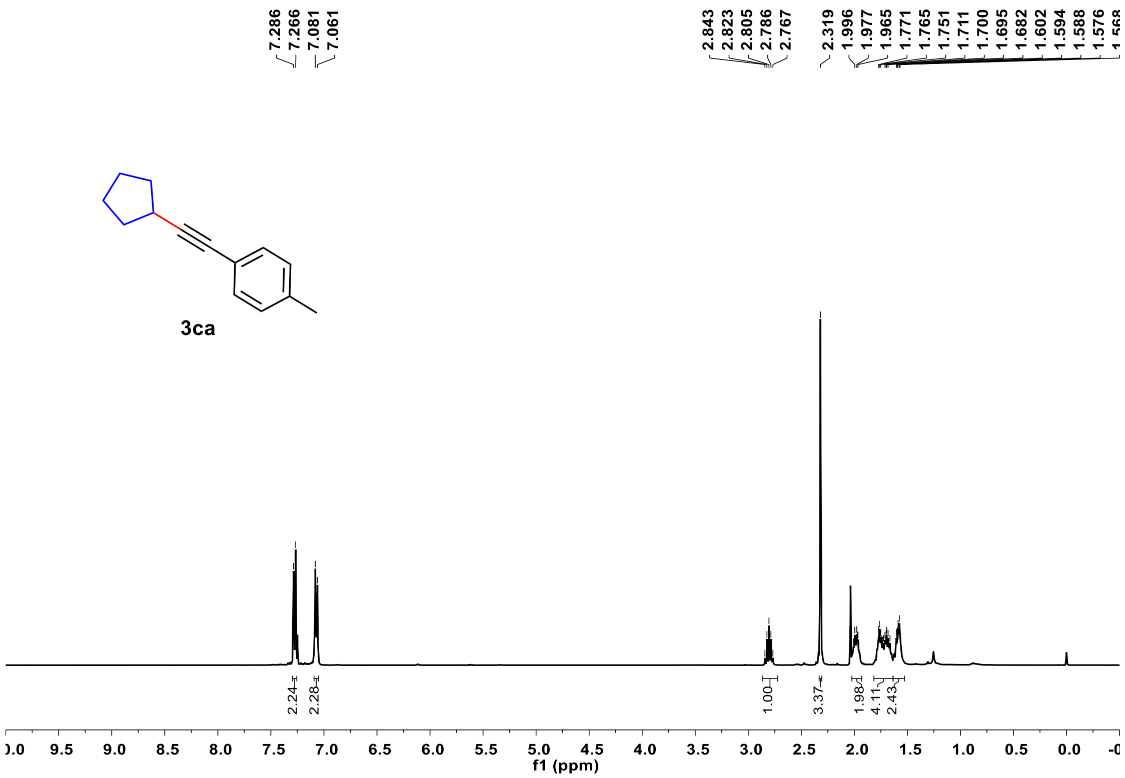

Supplementary Figure 5. **^{1}H NMR (400 MHz, $CDCl_3$) spectrum of 3ca**

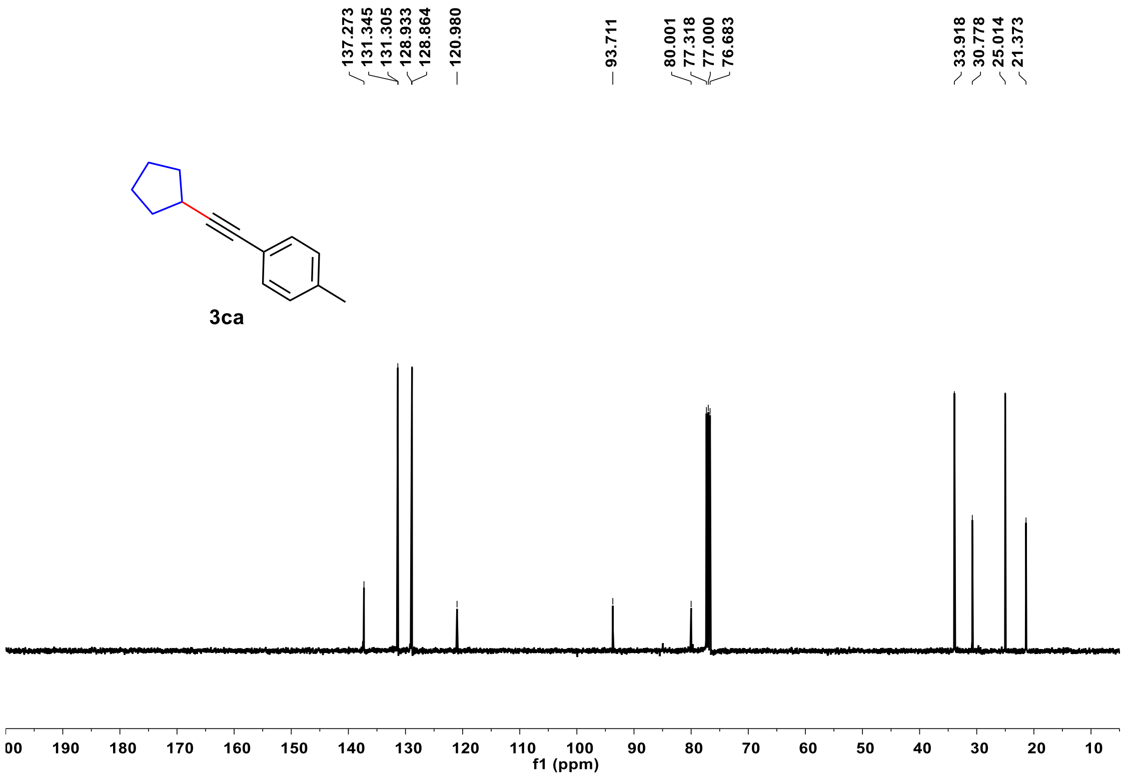

Supplementary Figure 6. **^{13}C NMR (101 MHz, $CDCl_3$) spectrum of 3ca**

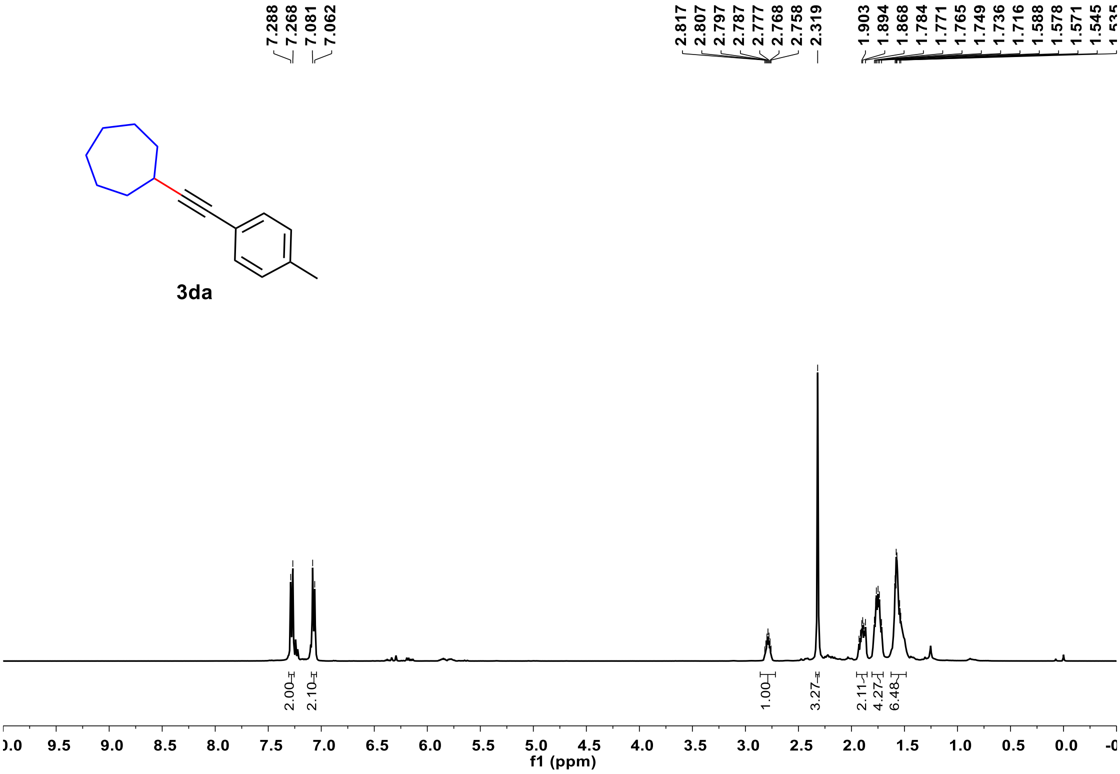

Supplementary Figure 7. ^{1}H NMR (400 MHz, $CDCl_3$) spectrum of 3da

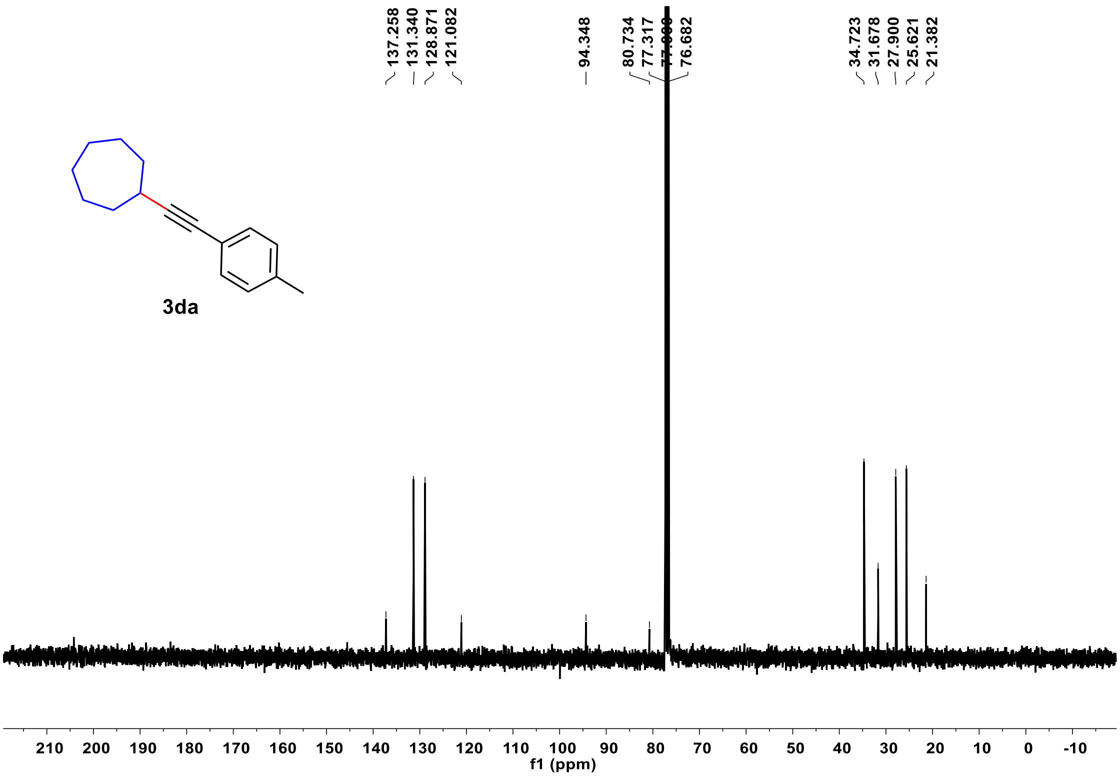

Supplementary Figure 8. ^{13}C NMR (101 MHz, $CDCl_3$) spectrum of 3da

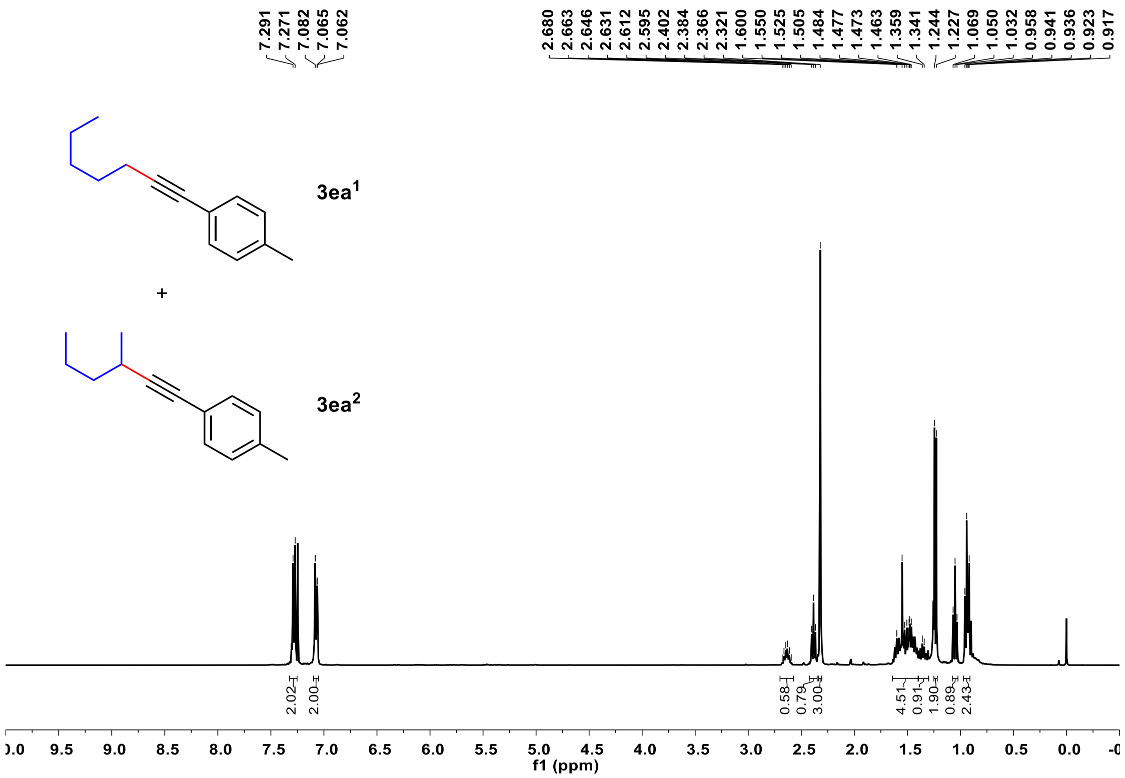

Supplementary Figure 9. ^{1}H **NMR (400 MHz,** $CDCl_3$**) spectrum of 3ea**

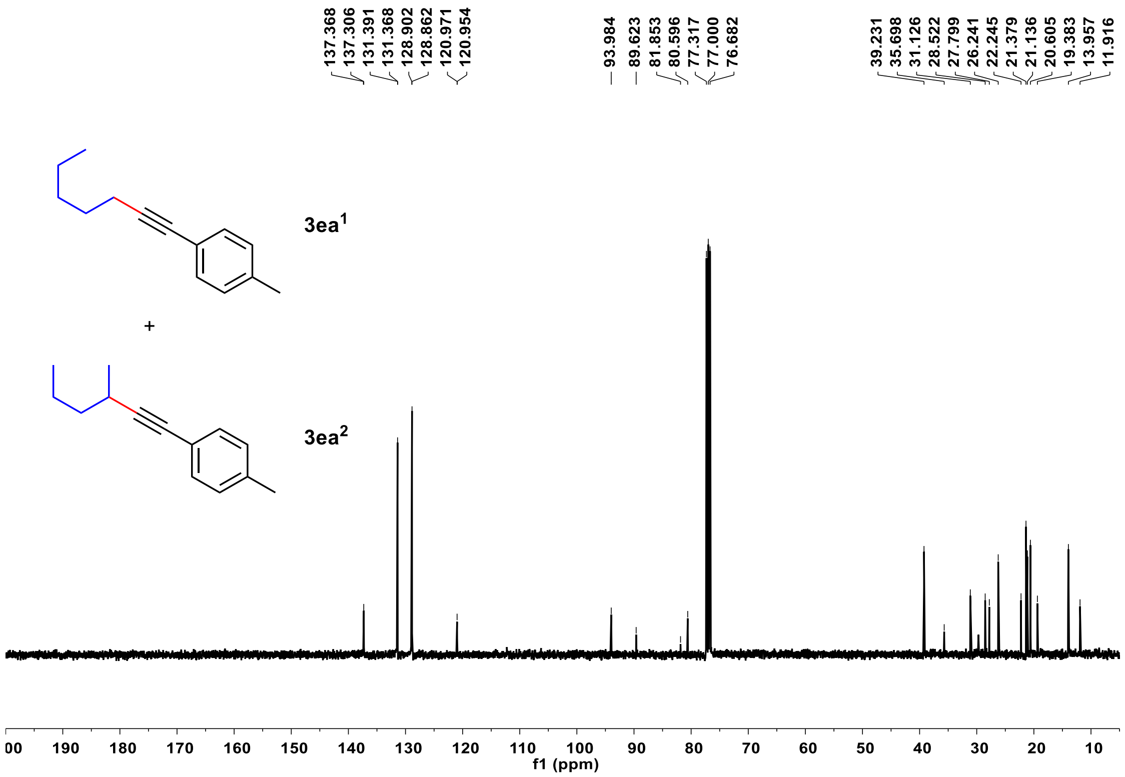

Supplementary Figure 10. ^{13}C **NMR (101 MHz,** $CDCl_3$**) spectrum of 3ea**

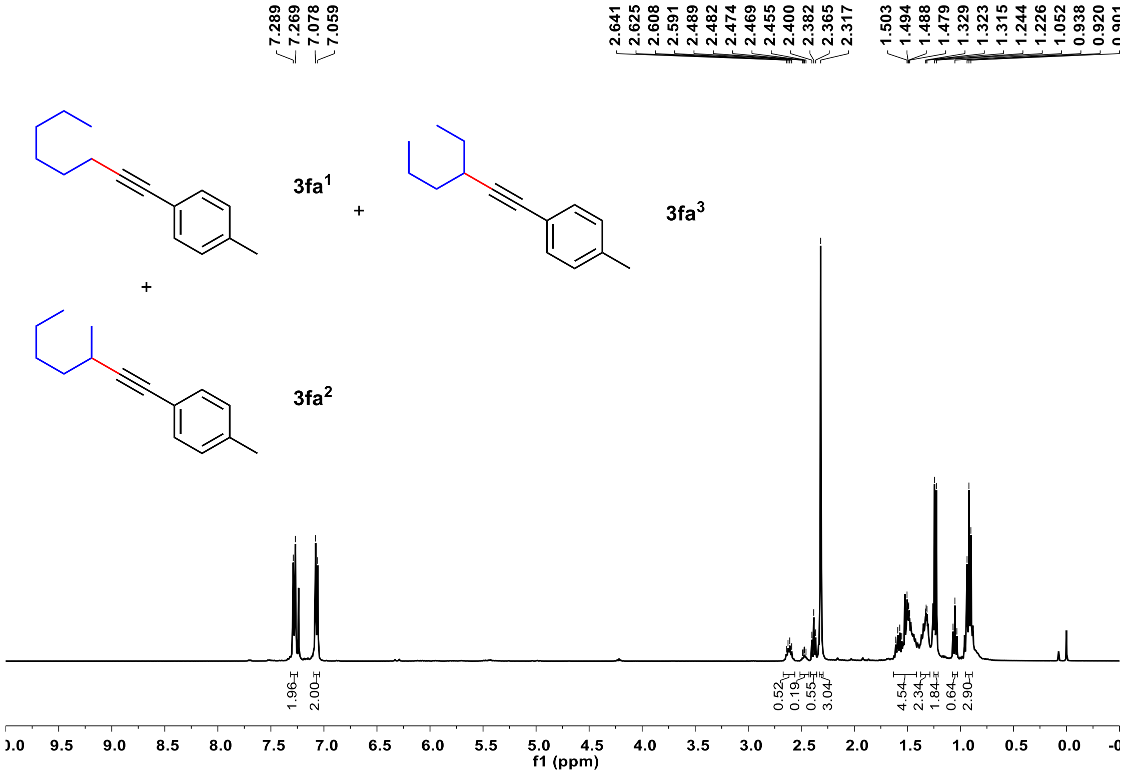

Supplementary Figure 11. ^{1}H NMR (400 MHz, $CDCl_3$) spectrum of 3fa

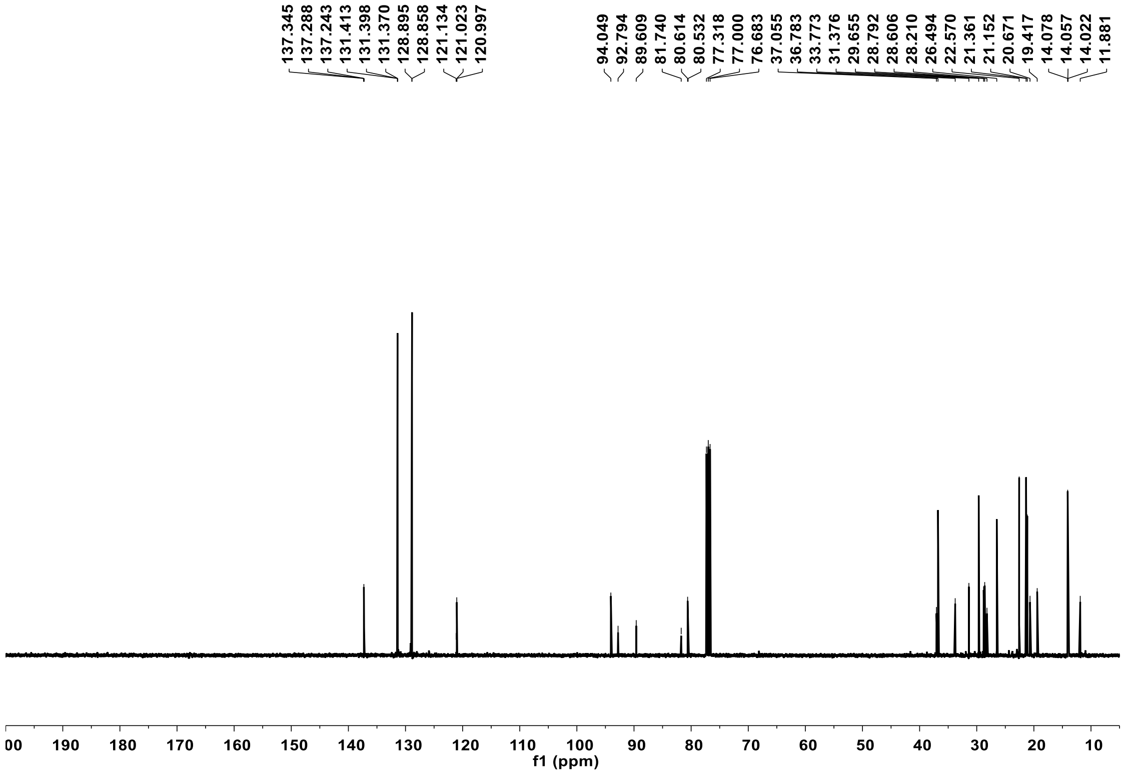

Supplementary Figure 12. ^{13}C NMR (101 MHz, $CDCl_3$) spectrum of 3fa

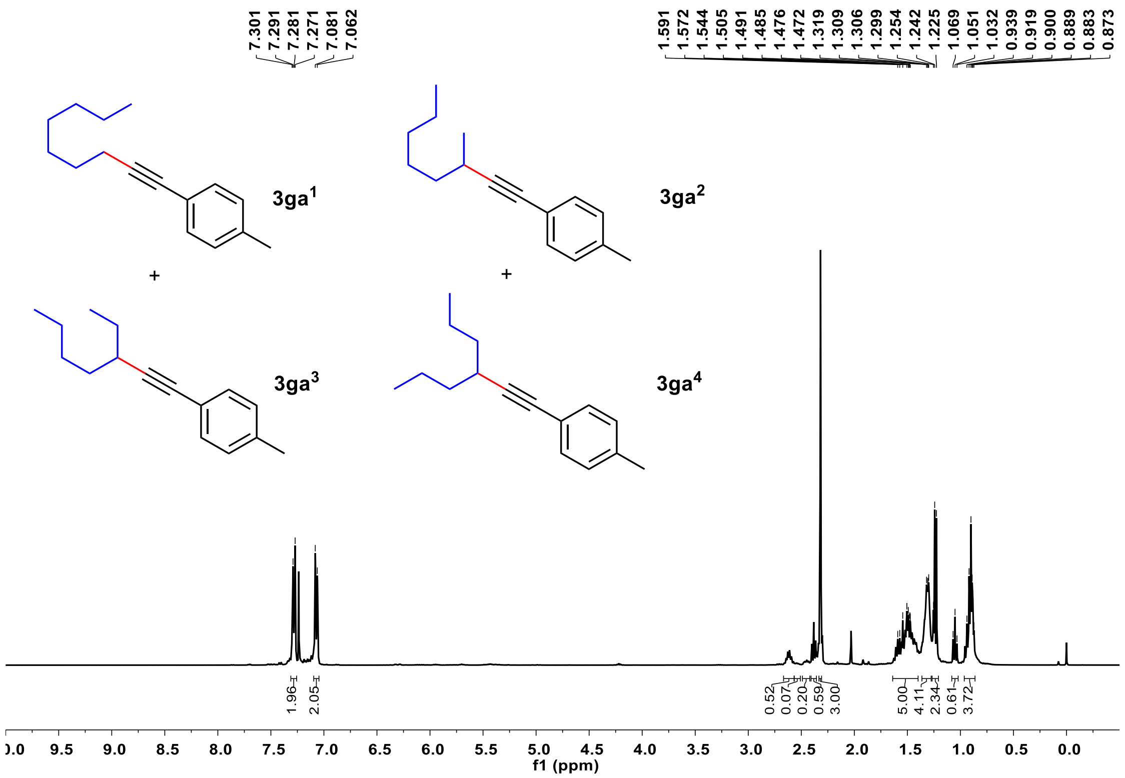

Supplementary Figure 13. ^{1}H NMR (400 MHz, $CDCl_3$) spectrum of 3ga

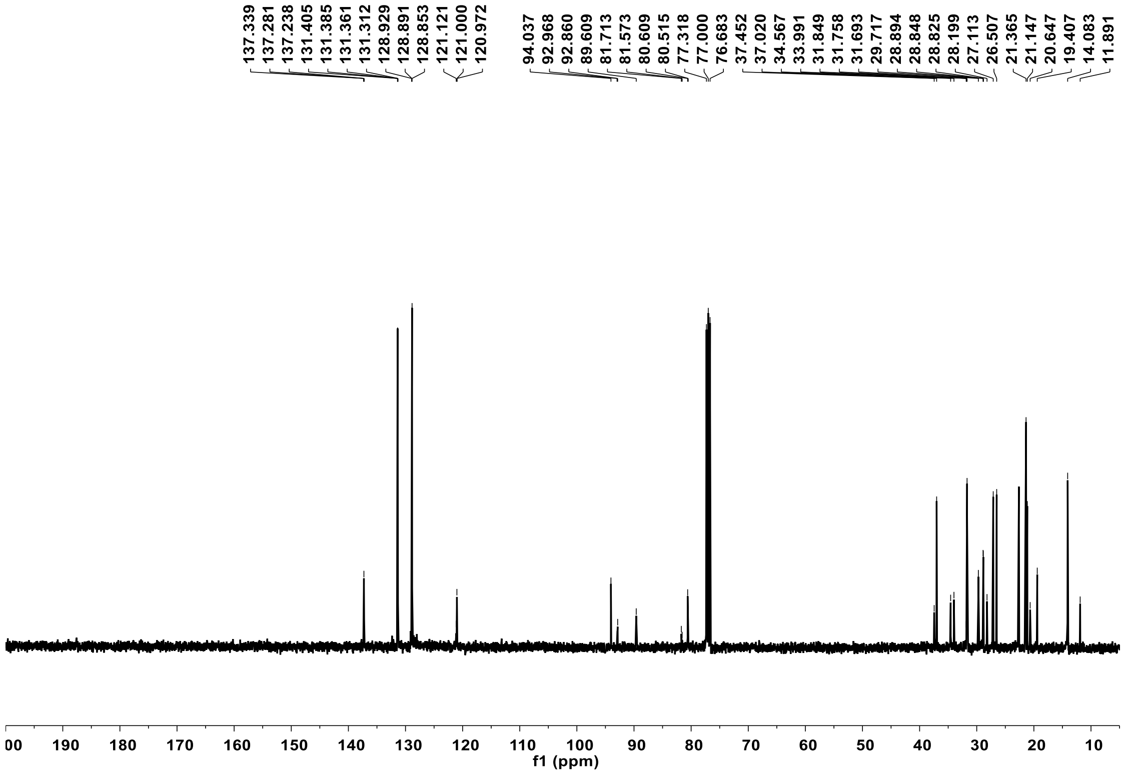

Supplementary Figure 14. ^{13}C NMR (101 MHz, $CDCl_3$) spectrum of 3ga

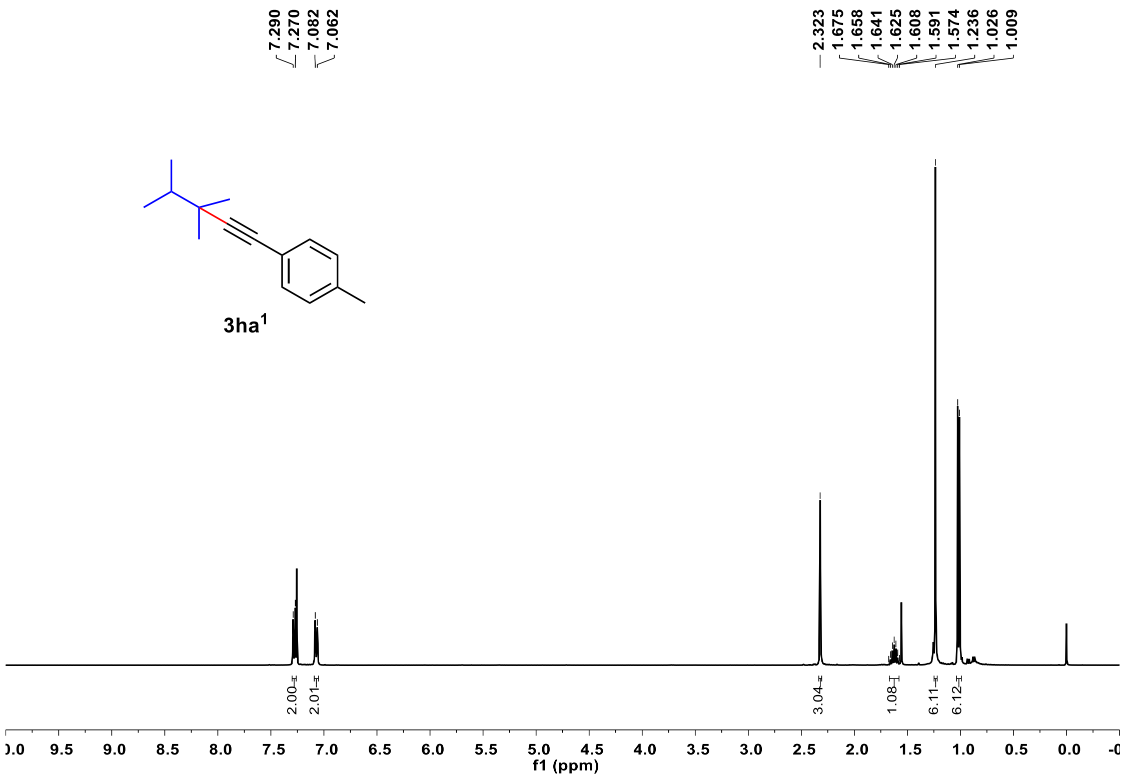

Supplementary Figure 15. **^{1}H NMR (400 MHz, $CDCl_3$) spectrum of 3ha[1]**

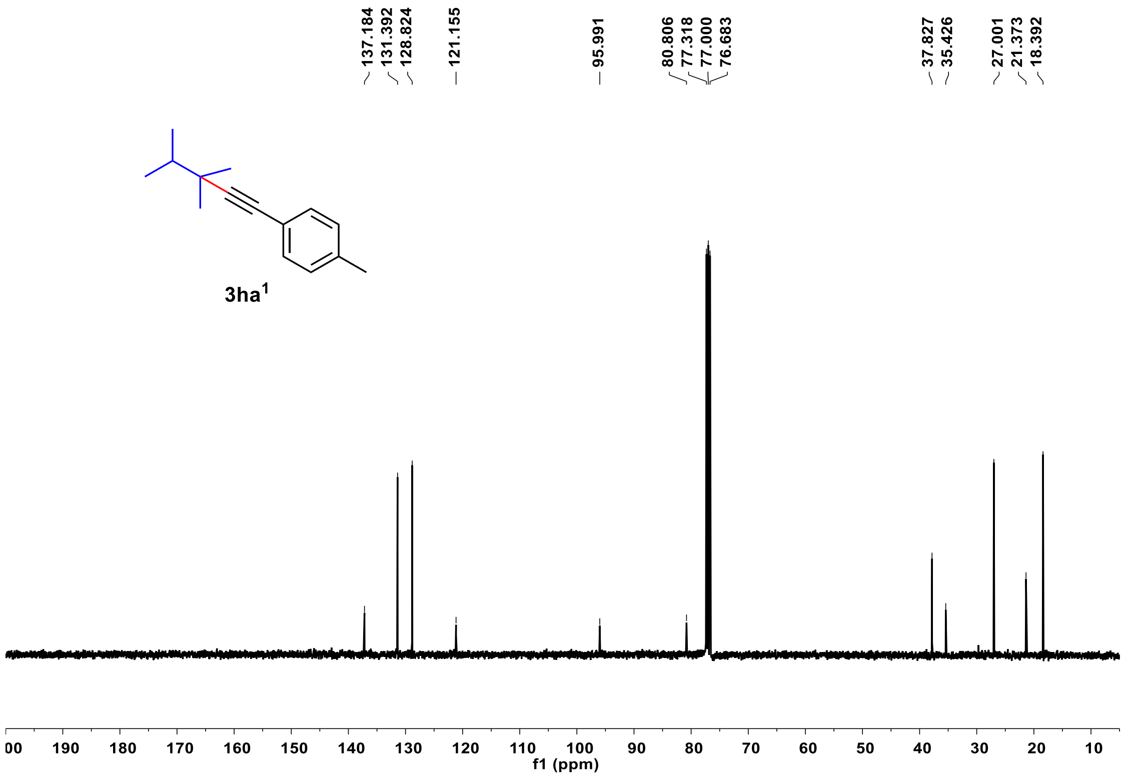

Supplementary Figure 16. **^{13}C NMR (101 MHz, $CDCl_3$) spectrum of 3ha[1]**

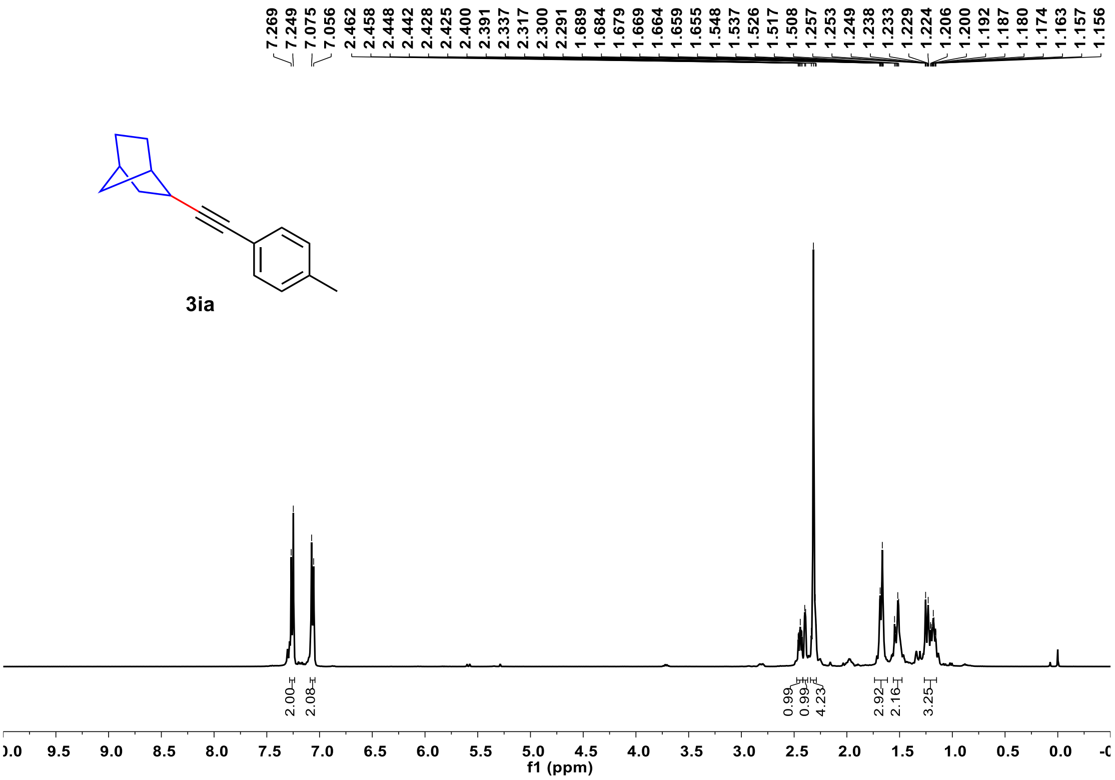

Supplementary Figure 17. ^{1}**H NMR (400 MHz, CDCl$_3$) spectrum of 3ia**

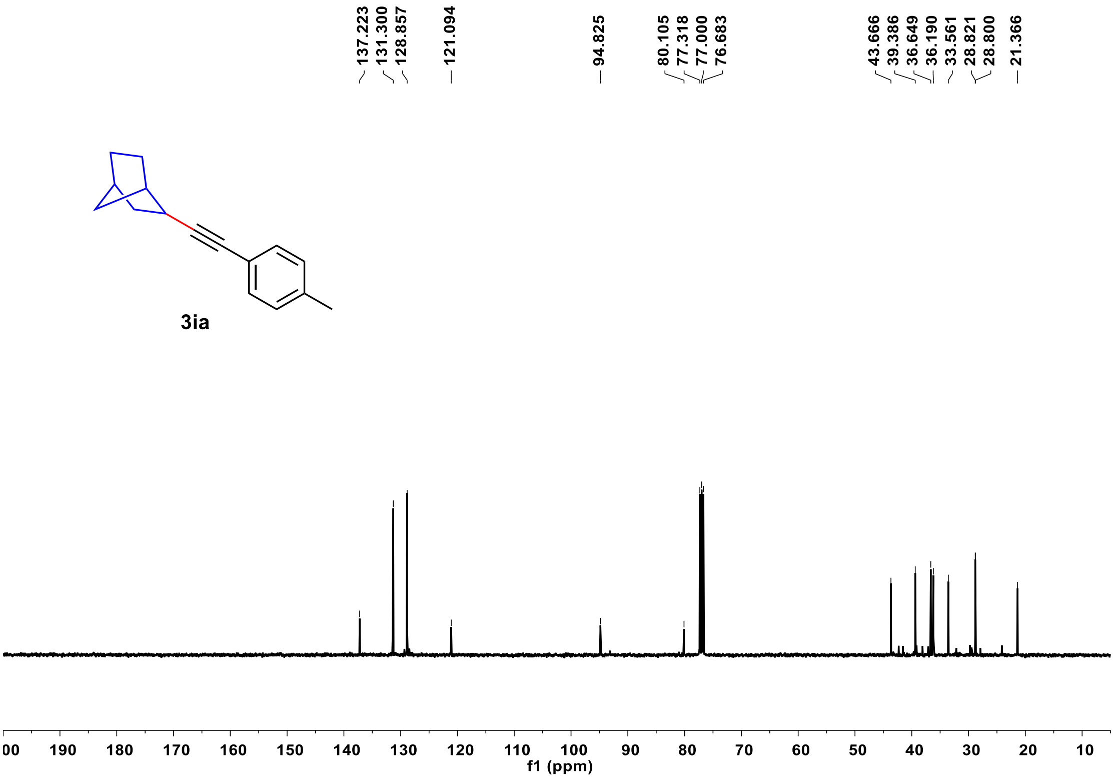

Supplementary Figure 18. ^{13}**C NMR (101 MHz, CDCl$_3$) spectrum of 3ia**

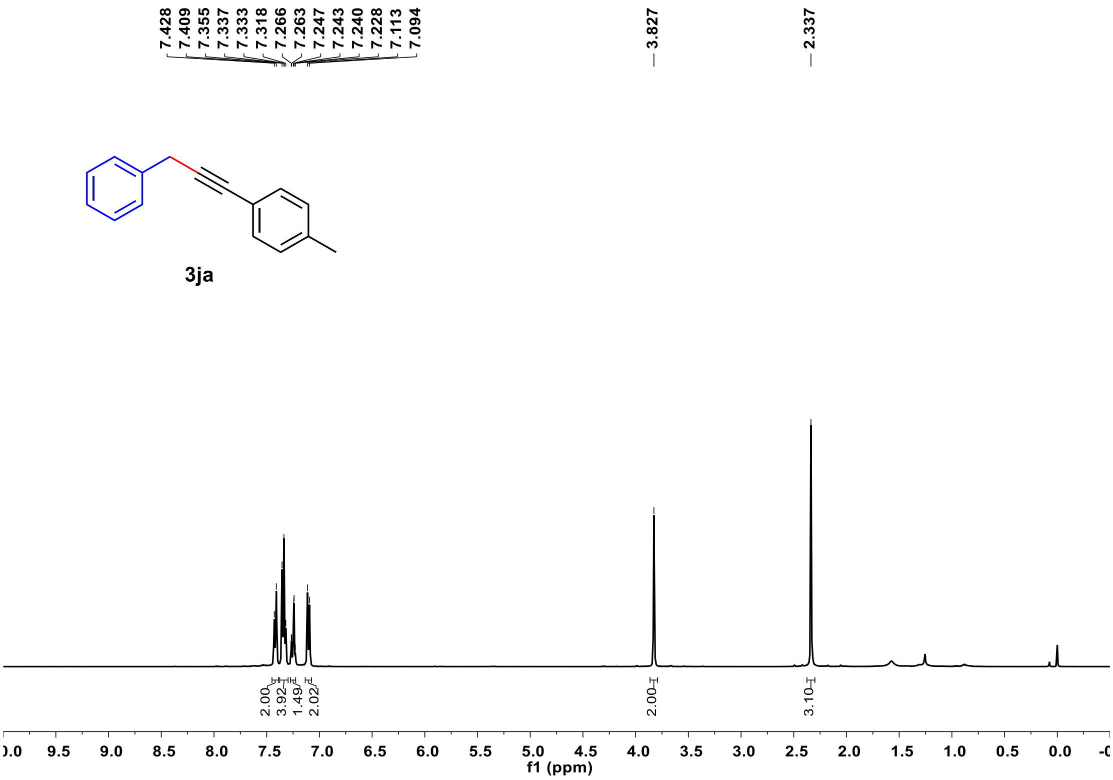

Supplementary Figure 19. ^{1}H **NMR (400 MHz,** $CDCl_3$**) spectrum of 3ja**

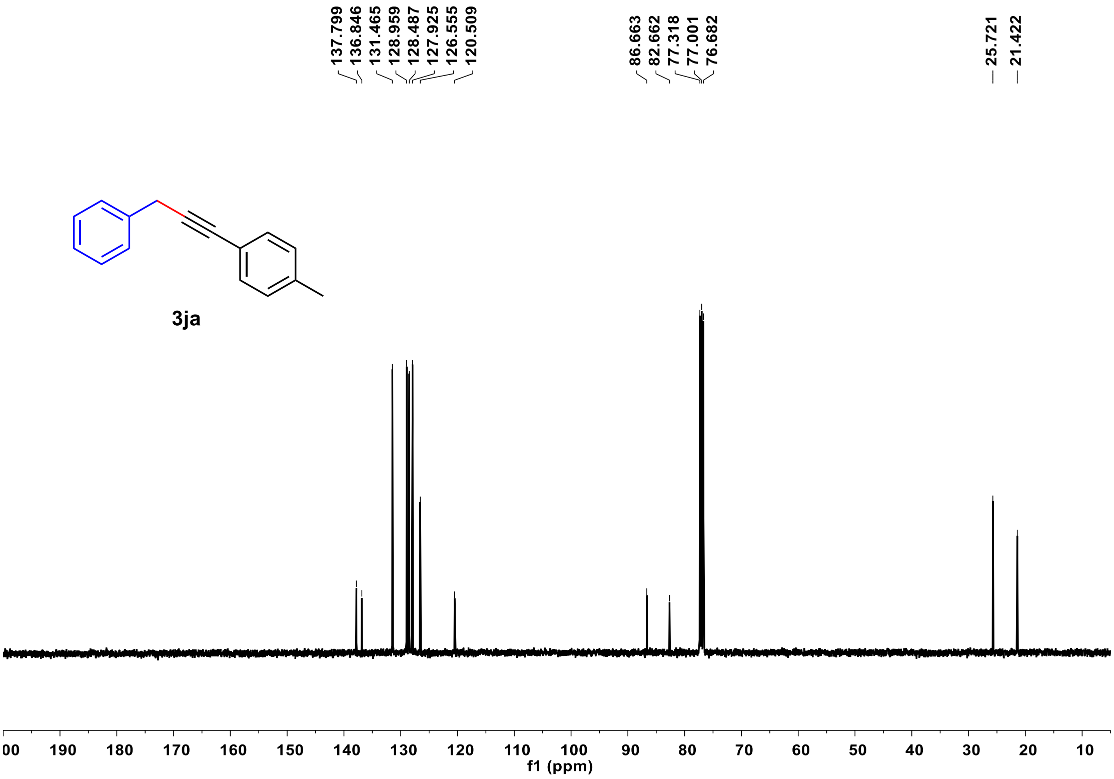

Supplementary Figure 20. ^{13}C **NMR (101 MHz,** $CDCl_3$**) spectrum of 3ja**

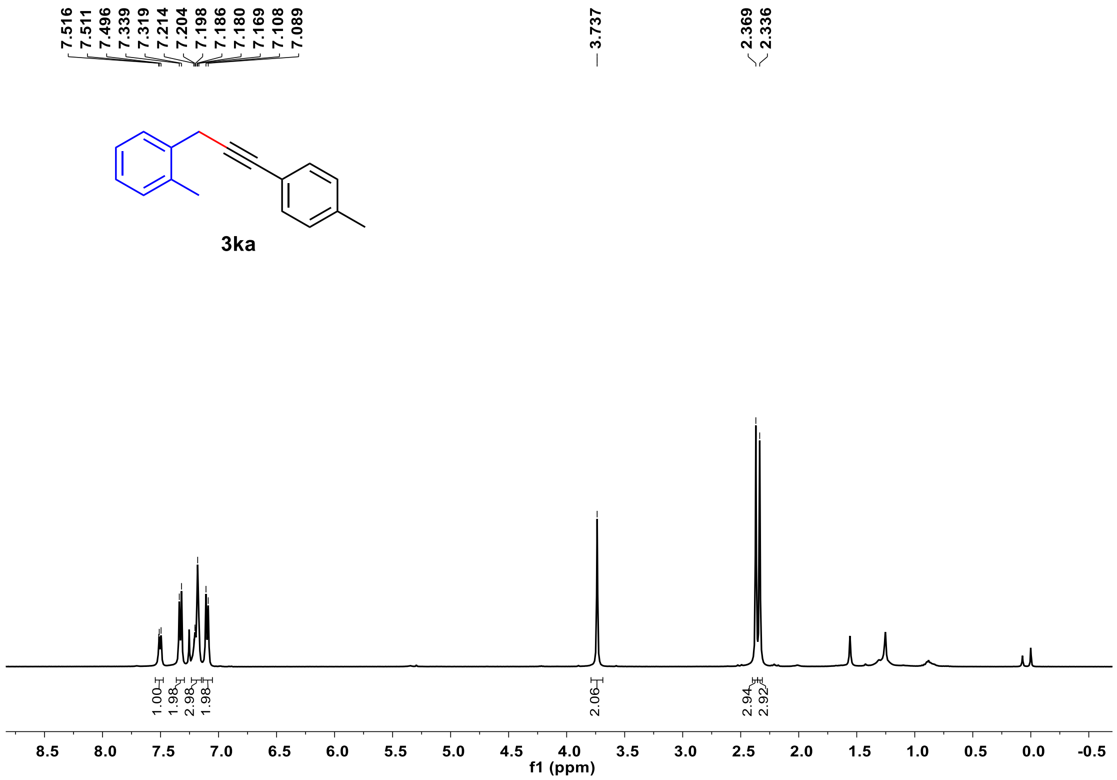

Supplementary Figure 21. ^{1}H **NMR (400 MHz, $CDCl_3$) spectrum of 3ka**

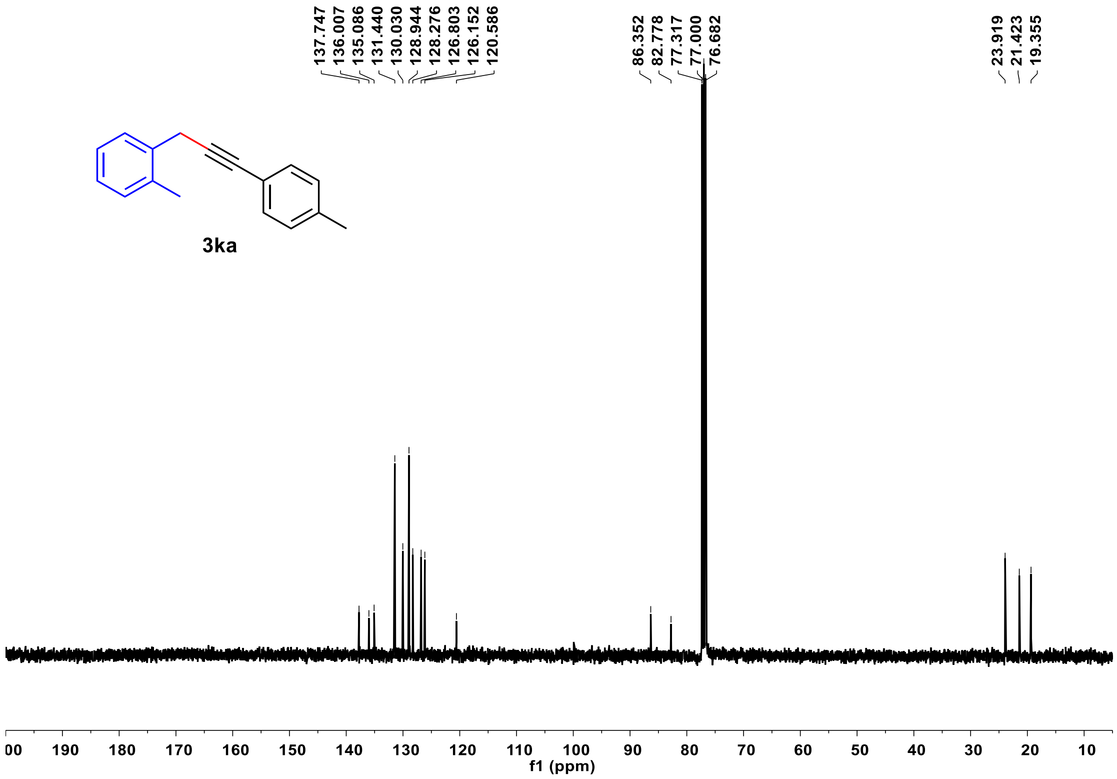

Supplementary Figure 22. ^{13}C **NMR (101 MHz, $CDCl_3$) spectrum of 3ka**

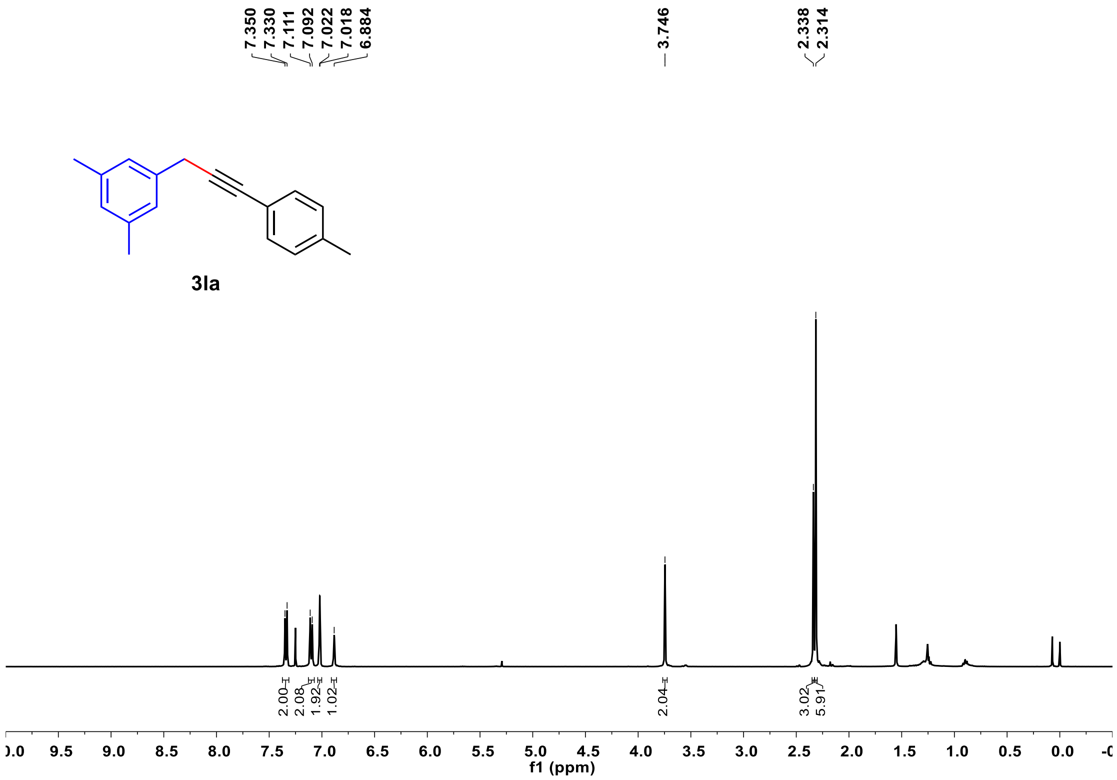

Supplementary Figure 23. ^{1}H NMR (400 MHz, $CDCl_3$) spectrum of 3la

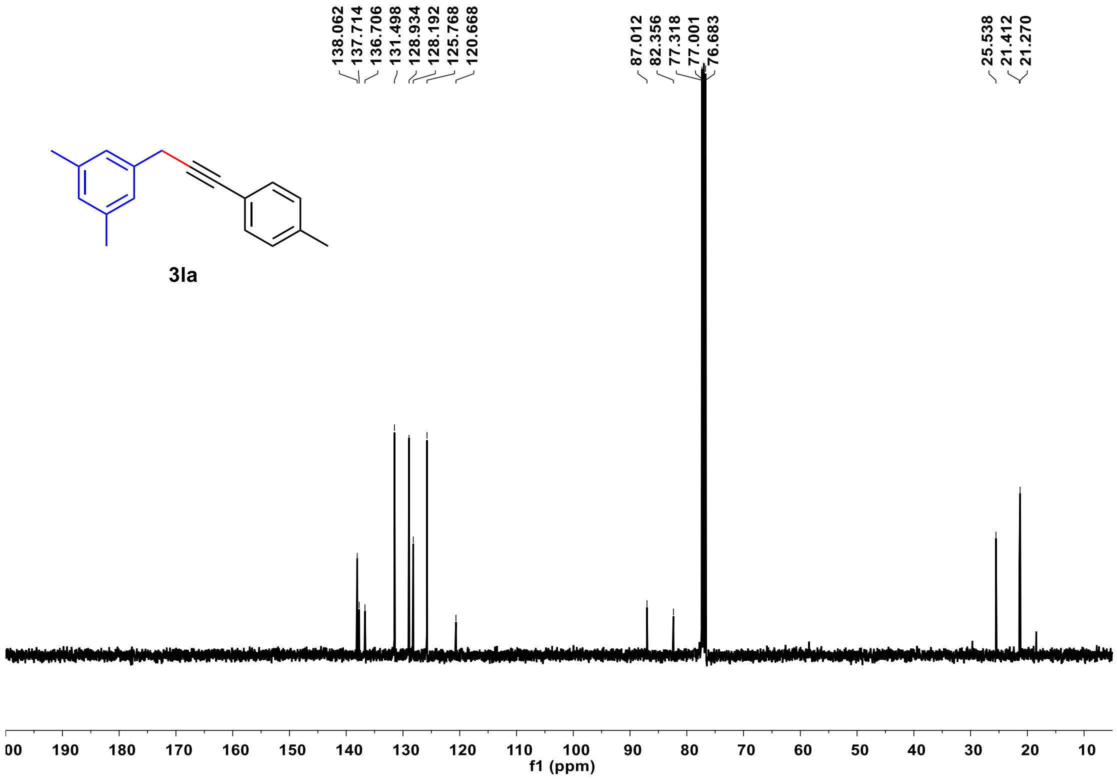

Supplementary Figure 24. ^{13}C NMR (101 MHz, $CDCl_3$) spectrum of 3la

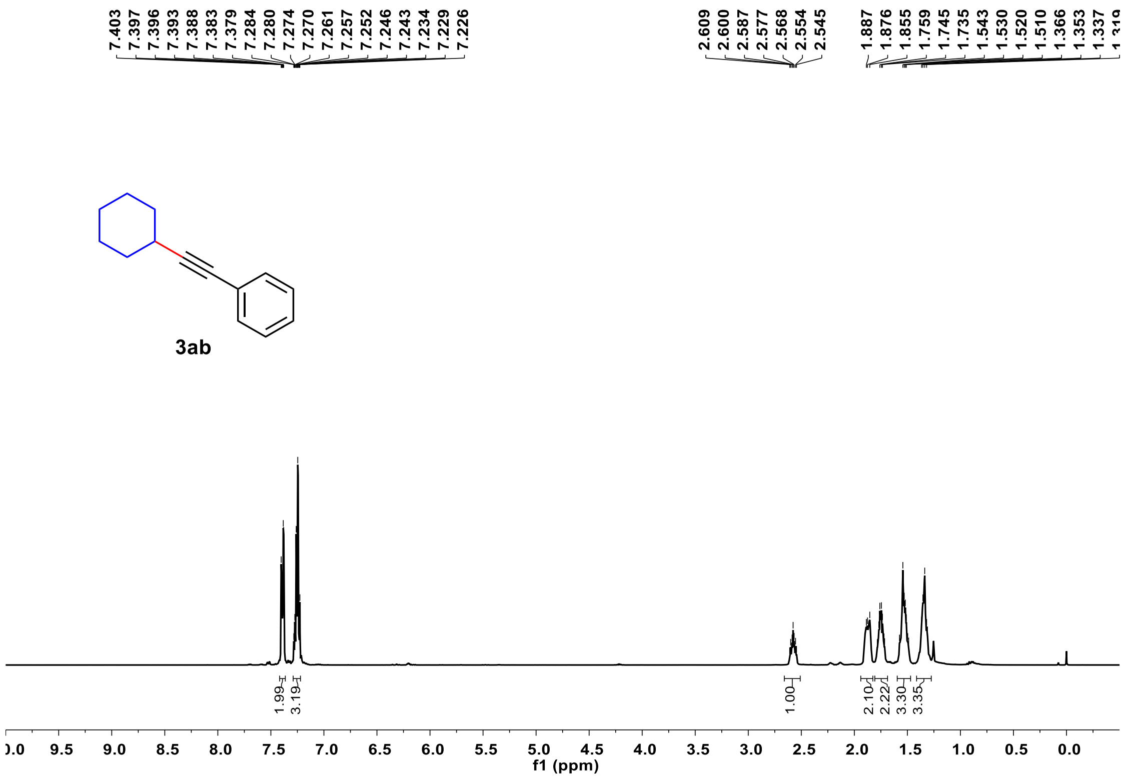

Supplementary Figure 25. ^{1}H NMR (400 MHz, $CDCl_3$) spectrum of 3ab

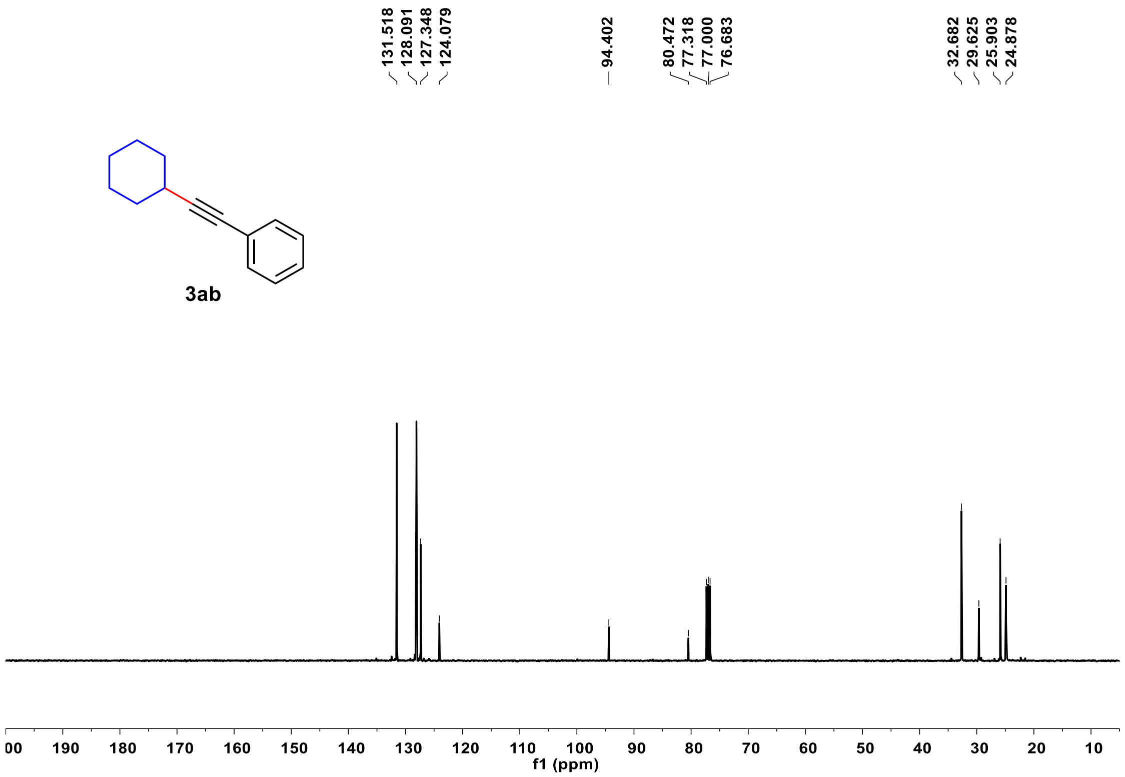

Supplementary Figure 26. ^{13}C NMR (101 MHz, $CDCl_3$) spectrum of 3ab

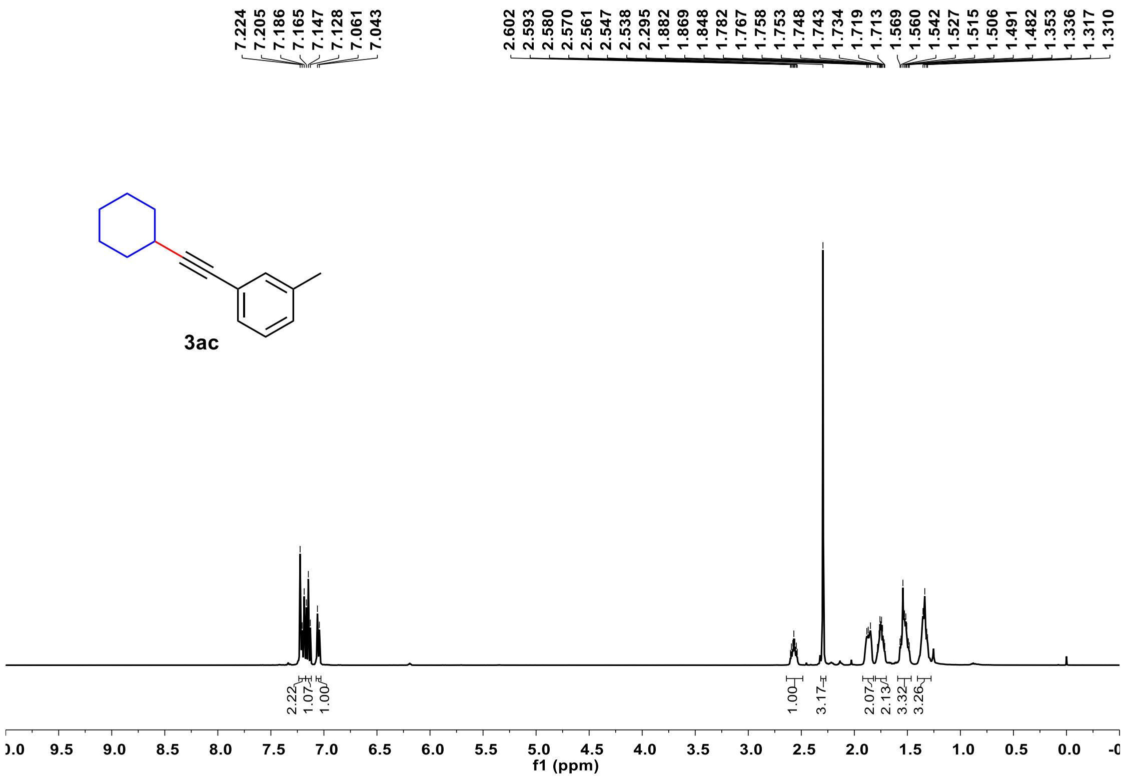

Supplementary Figure 27. ^{1}H NMR (400 MHz, $CDCl_3$) spectrum of 3ac

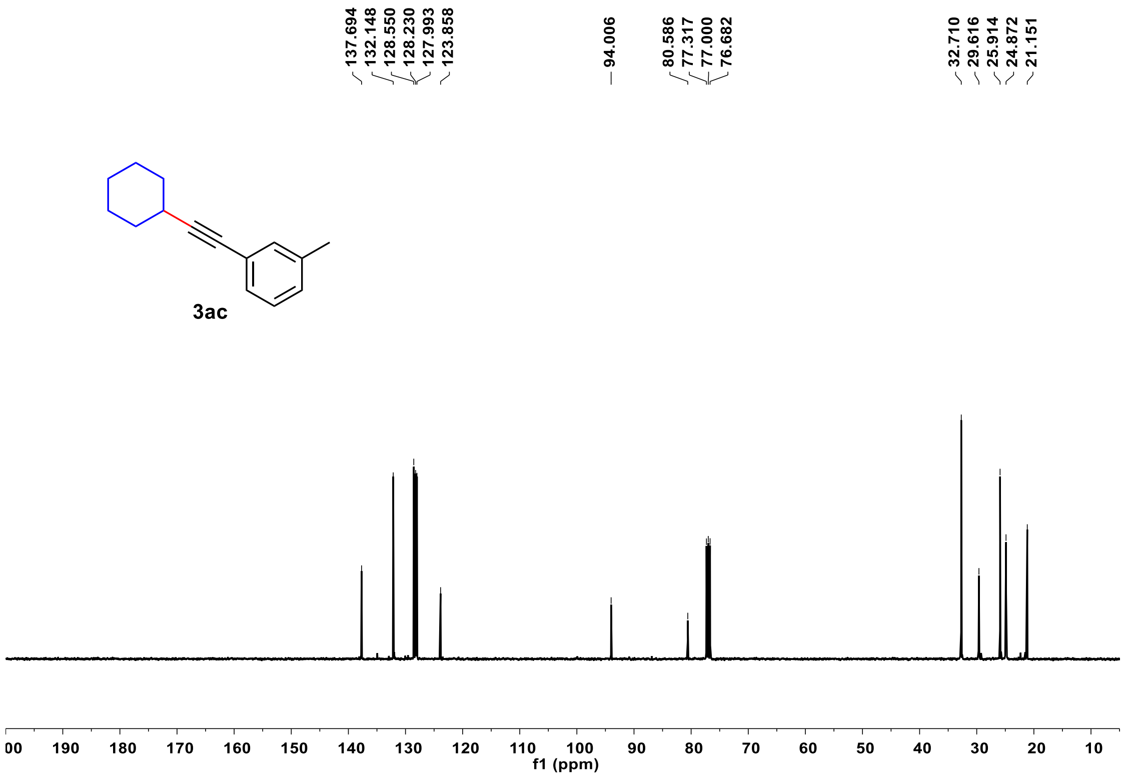

Supplementary Figure 28. ^{13}C NMR (101 MHz, $CDCl_3$) spectrum of 3ac

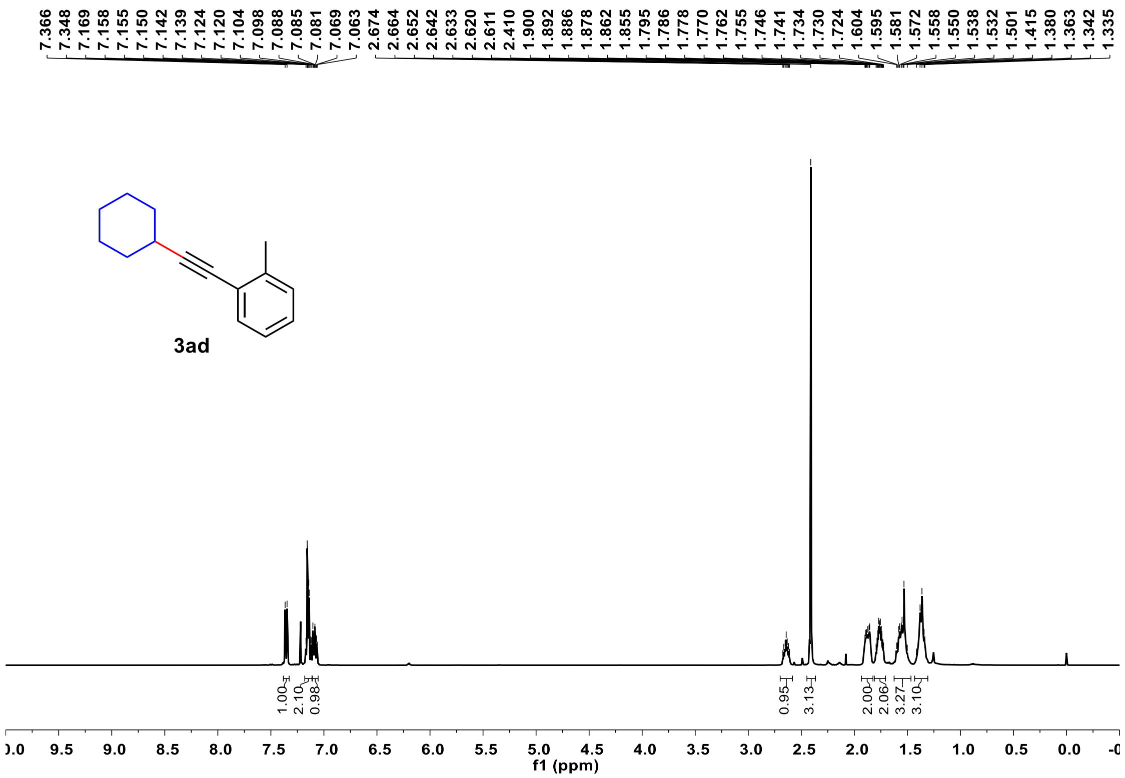

Supplementary Figure 29. ^{1}H **NMR (400 MHz,** $CDCl_3$**) spectrum of 3ad**

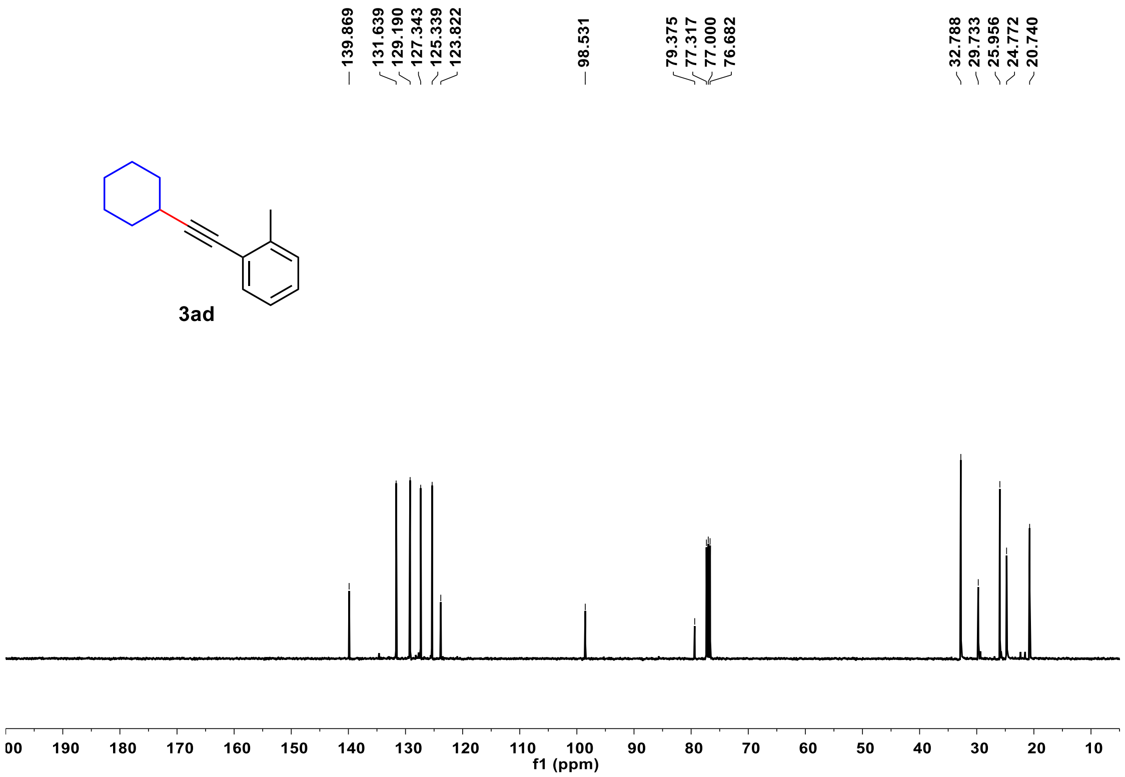

Supplementary Figure 30. ^{13}C **NMR (101 MHz,** $CDCl_3$**) spectrum of 3ad**

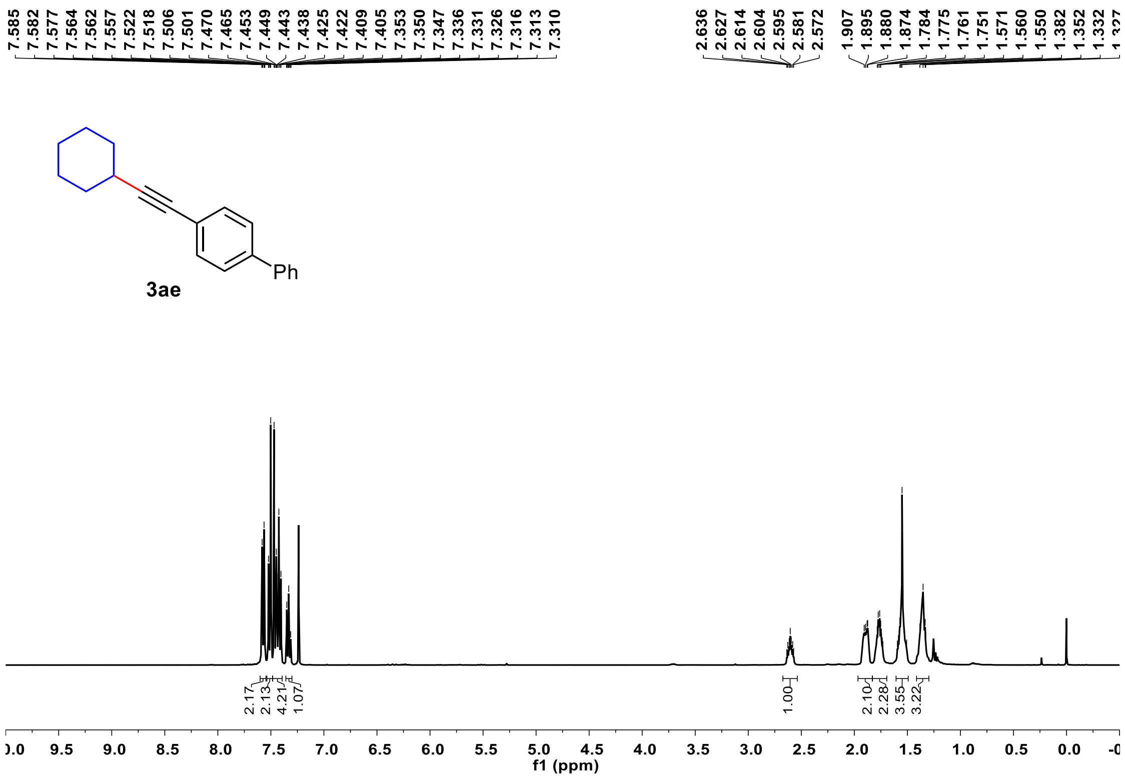

Supplementary Figure 31. ^{1}H NMR (400 MHz, $CDCl_3$) spectrum of 3ae

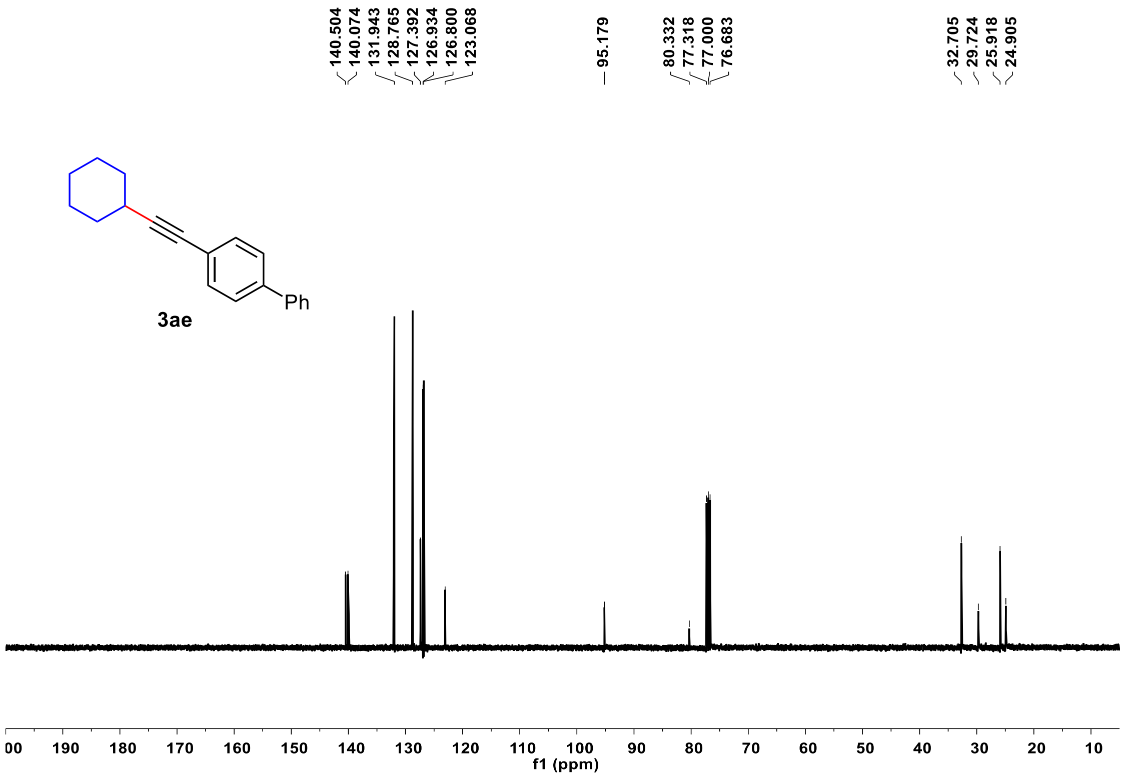

Supplementary Figure 32. ^{13}C NMR (101 MHz, $CDCl_3$) spectrum of 3ae

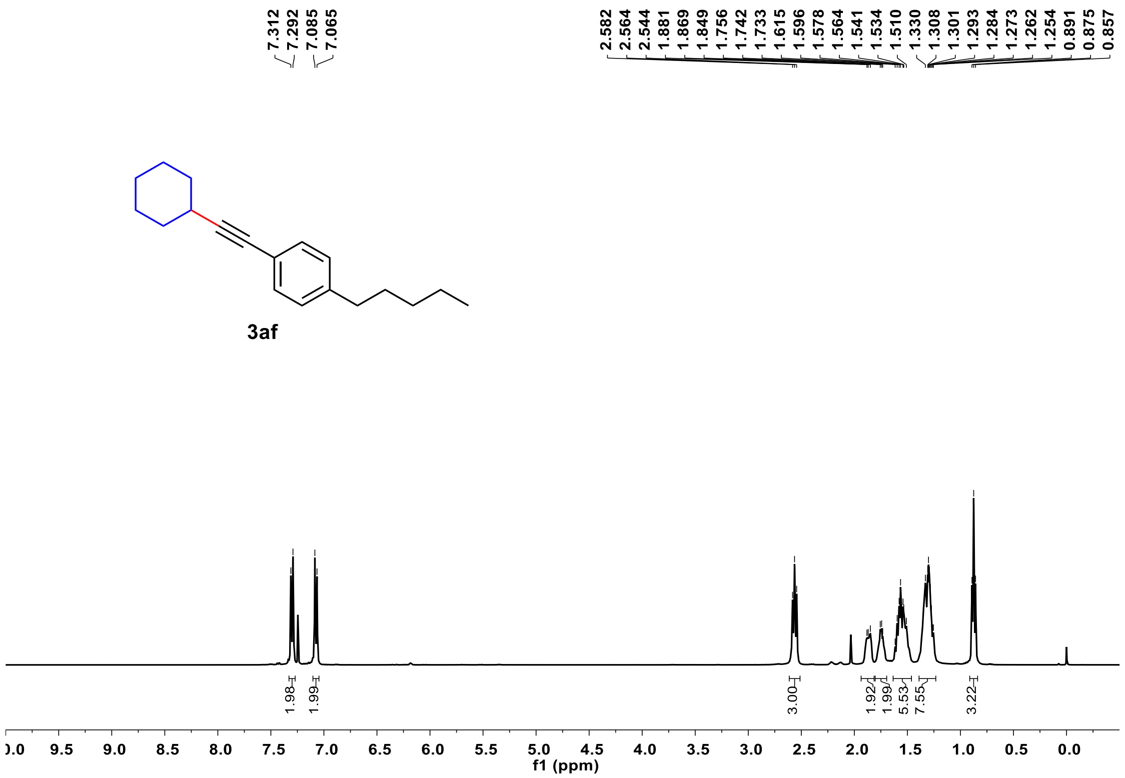

Supplementary Figure 33. ^{1}H NMR (400 MHz, $CDCl_3$) spectrum of 3af

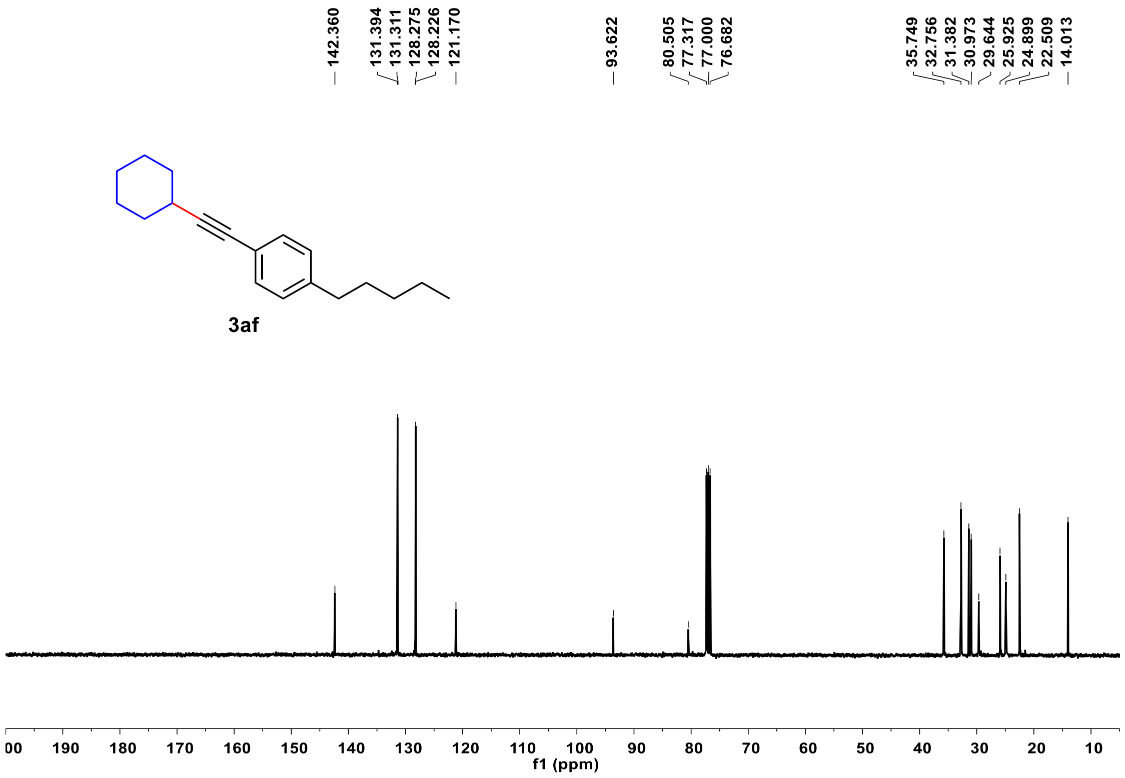

Supplementary Figure 34. ^{13}C NMR (101 MHz, $CDCl_3$) spectrum of 3af

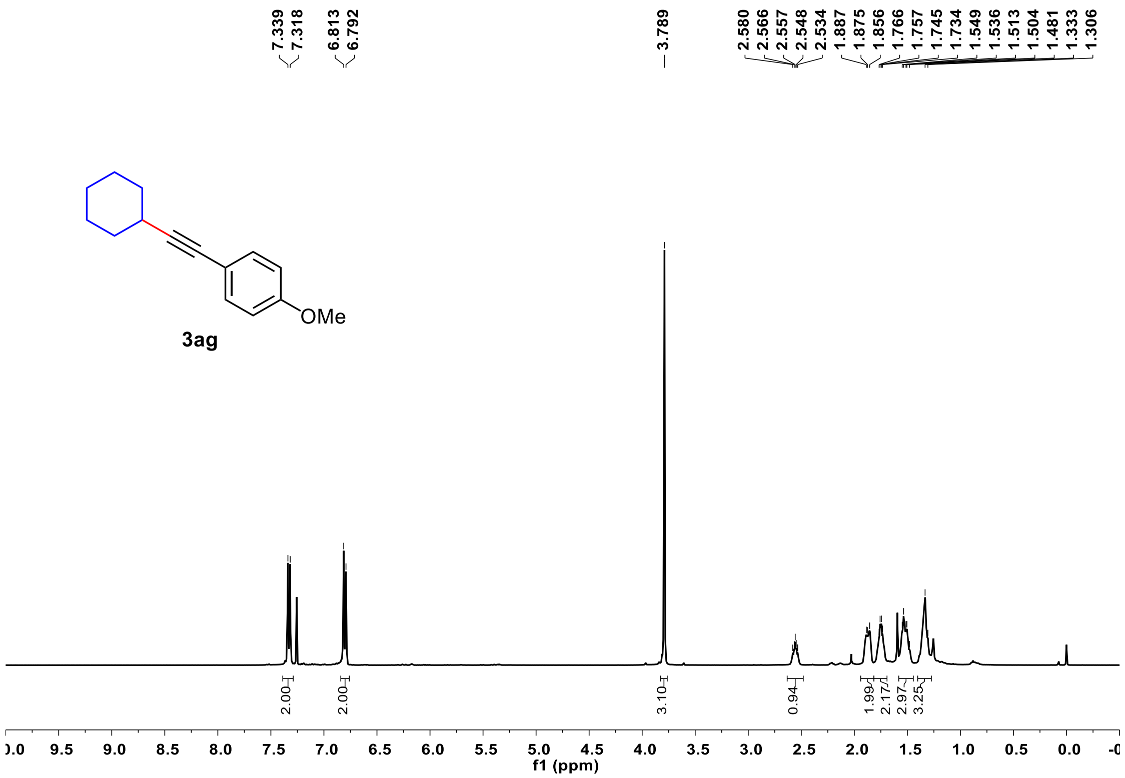

Supplementary Figure 35. ^{1}H NMR (400 MHz, $CDCl_3$) spectrum of 3ag

^{13}C NMR

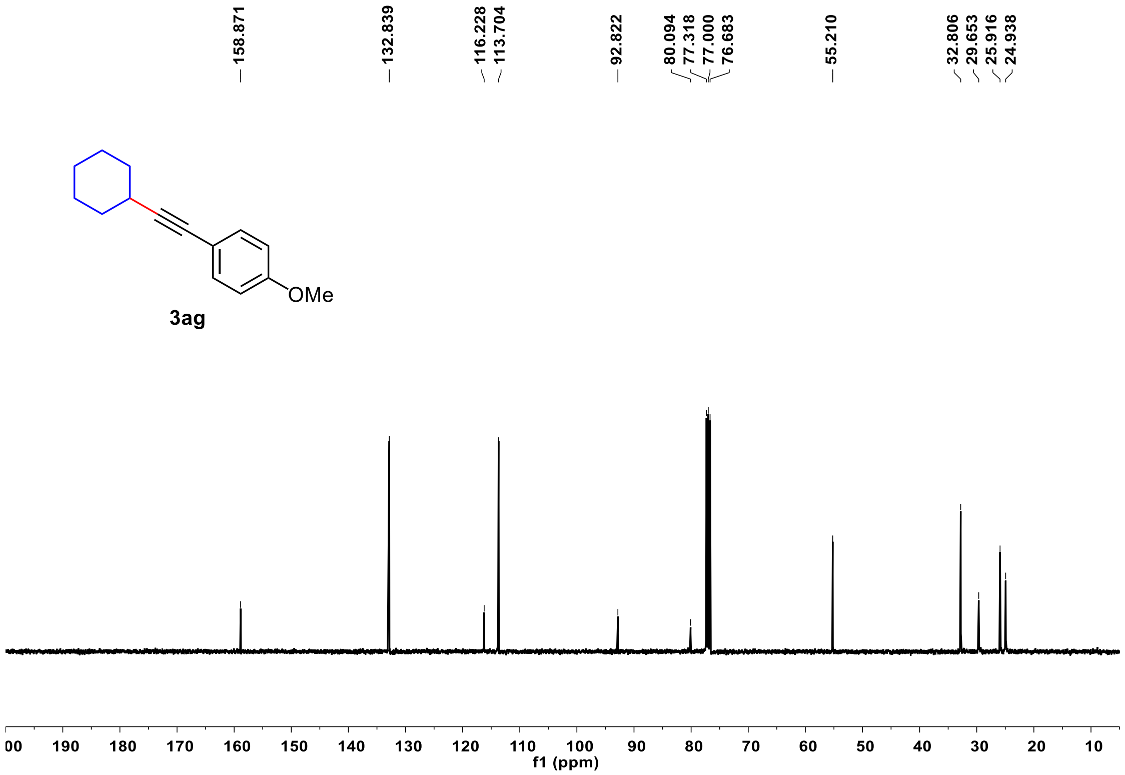

Supplementary Figure 36. ^{13}C NMR (101 MHz, $CDCl_3$) spectrum of 3ag

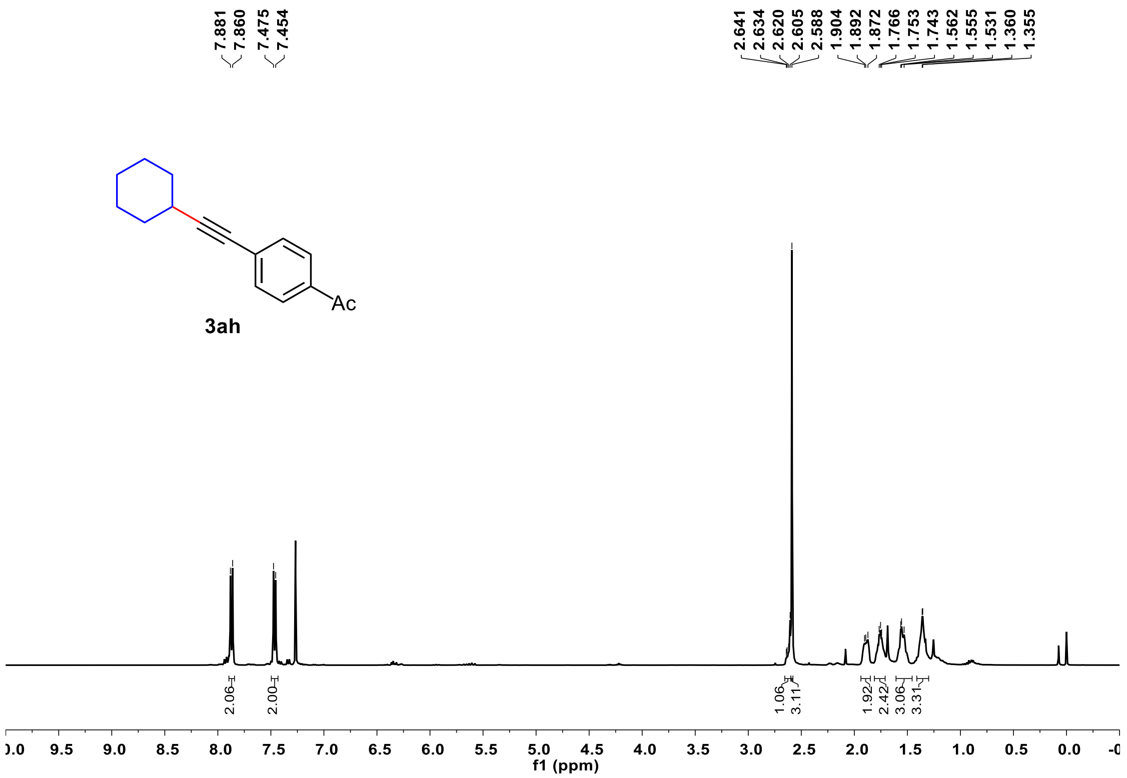

Supplementary Figure 37. ^{1}H NMR (400 MHz, $CDCl_3$) spectrum of 3ah

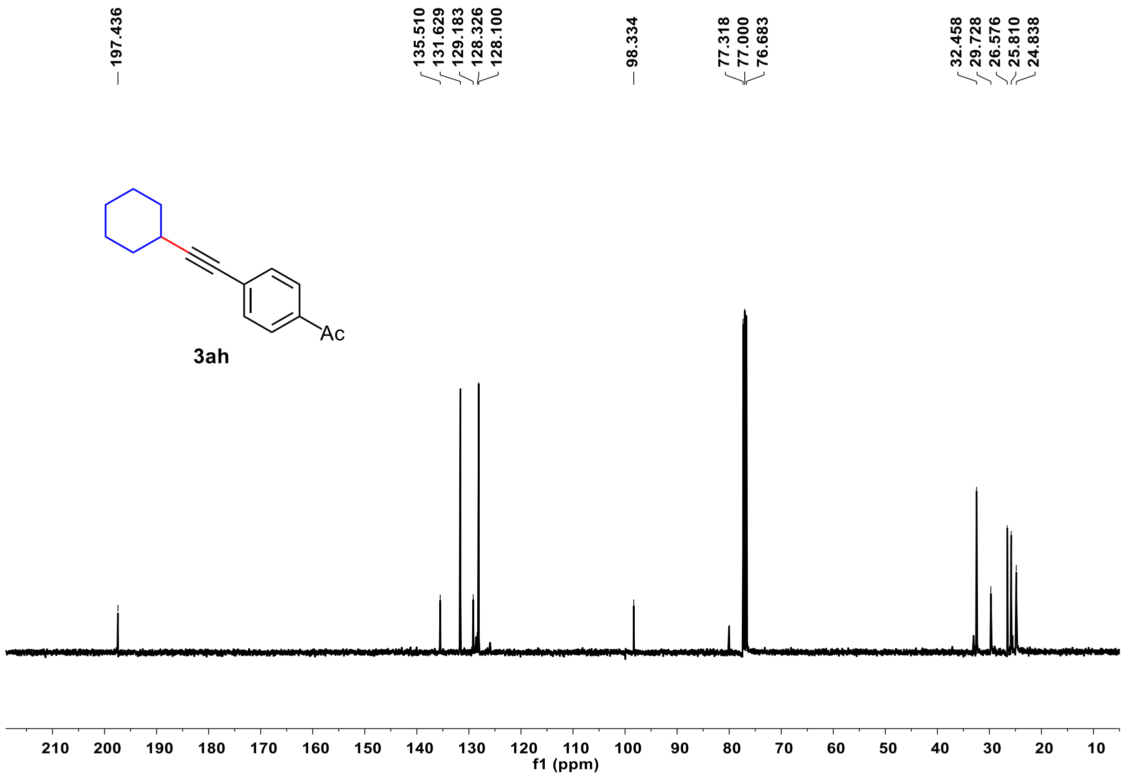

Supplementary Figure 38. ^{13}C NMR (101 MHz, $CDCl_3$) spectrum of 3ah

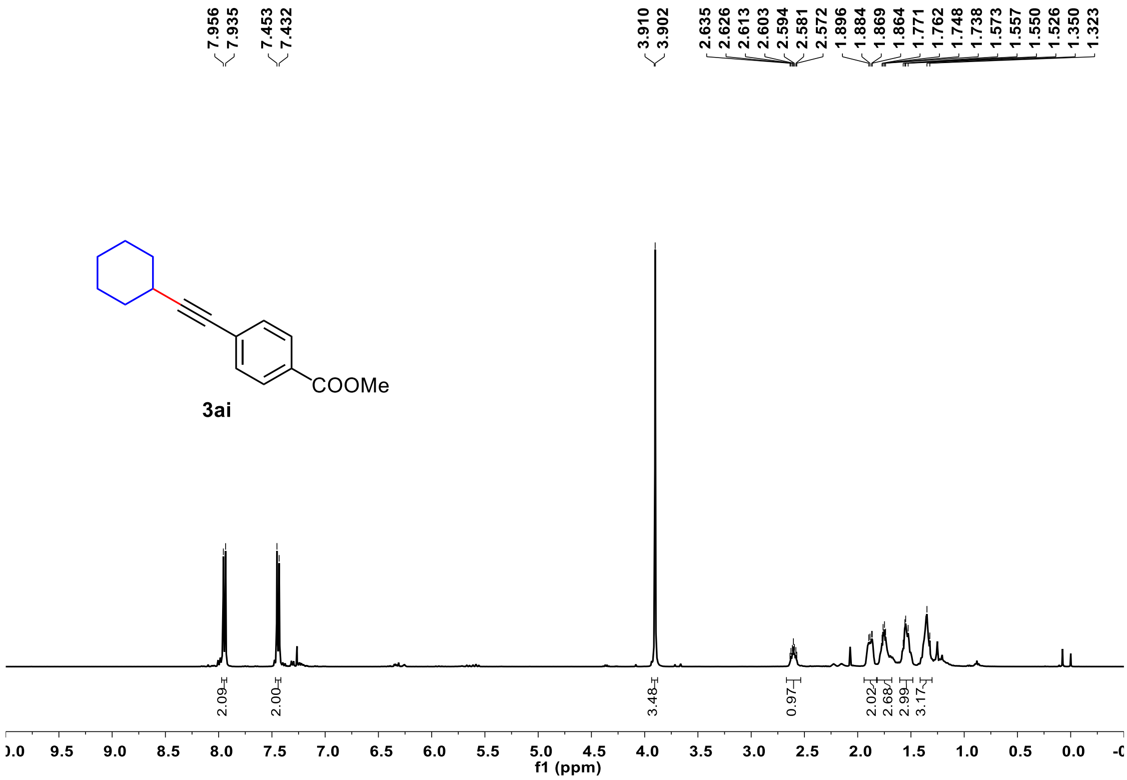

Supplementary Figure 39. **^{1}H NMR (400 MHz, $CDCl_3$) spectrum of 3ai**

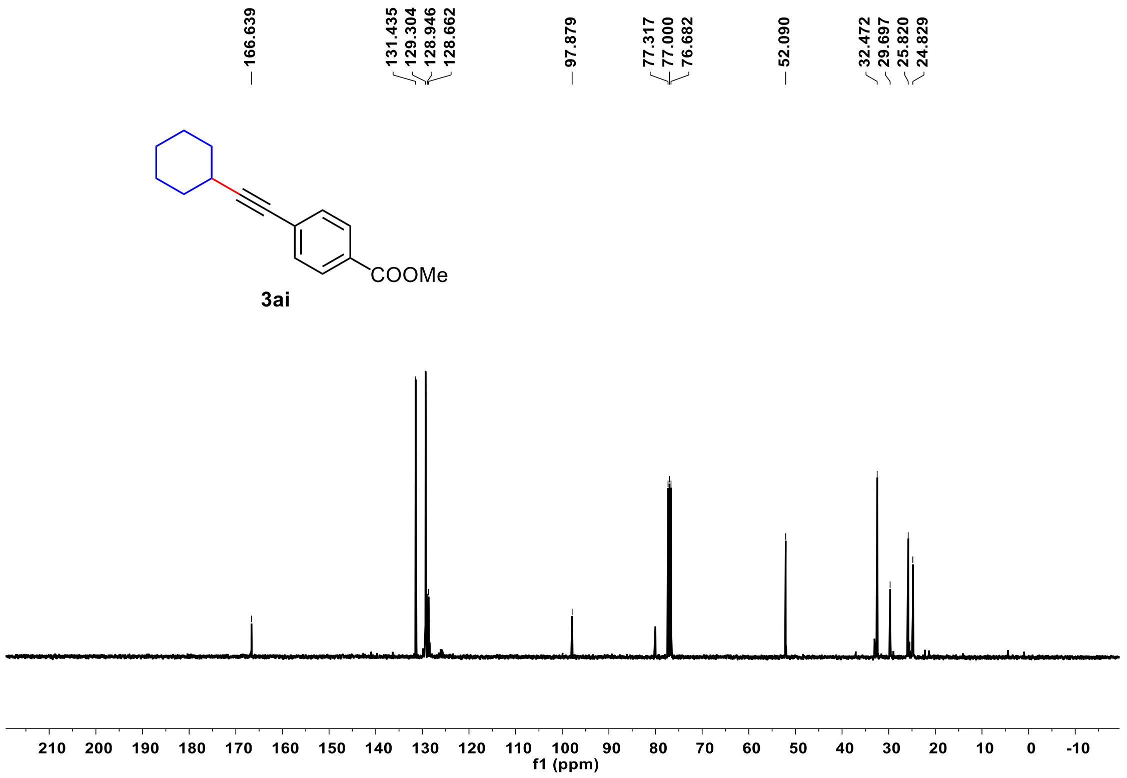

Supplementary Figure 40. **^{13}C NMR (101 MHz, $CDCl_3$) spectrum of 3ai**

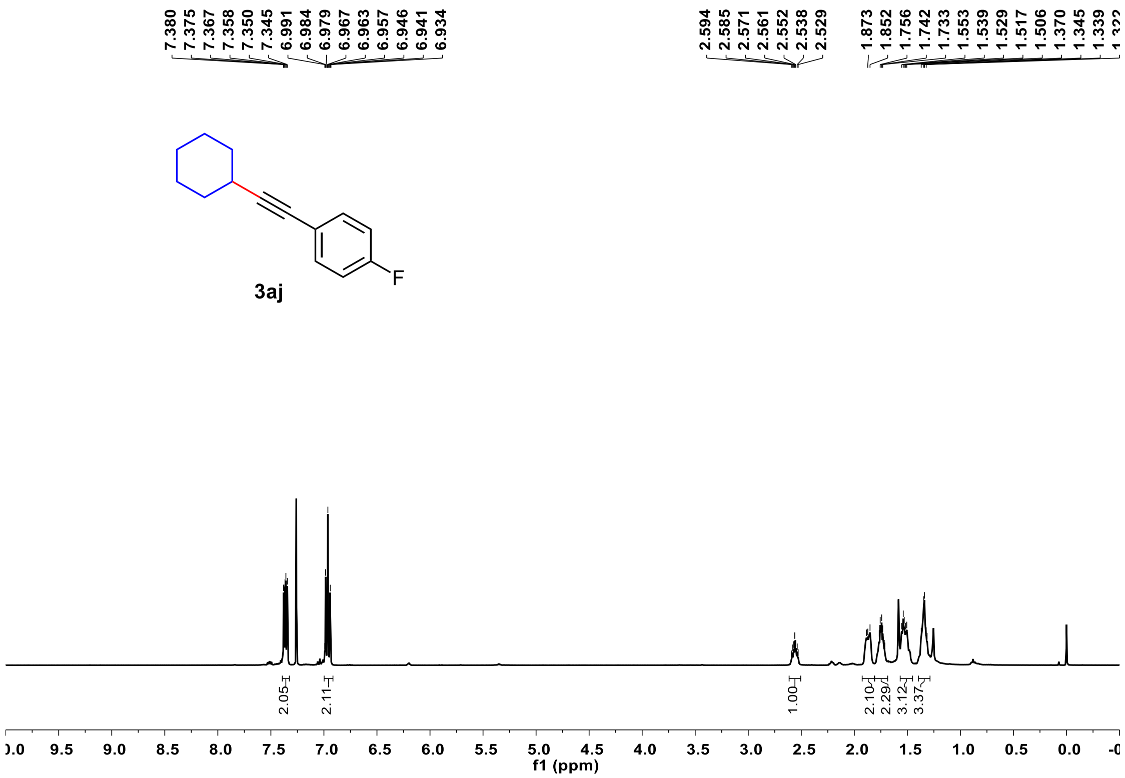

Supplementary Figure 41. ^{1}H NMR (400 MHz, $CDCl_3$) spectrum of 3aj

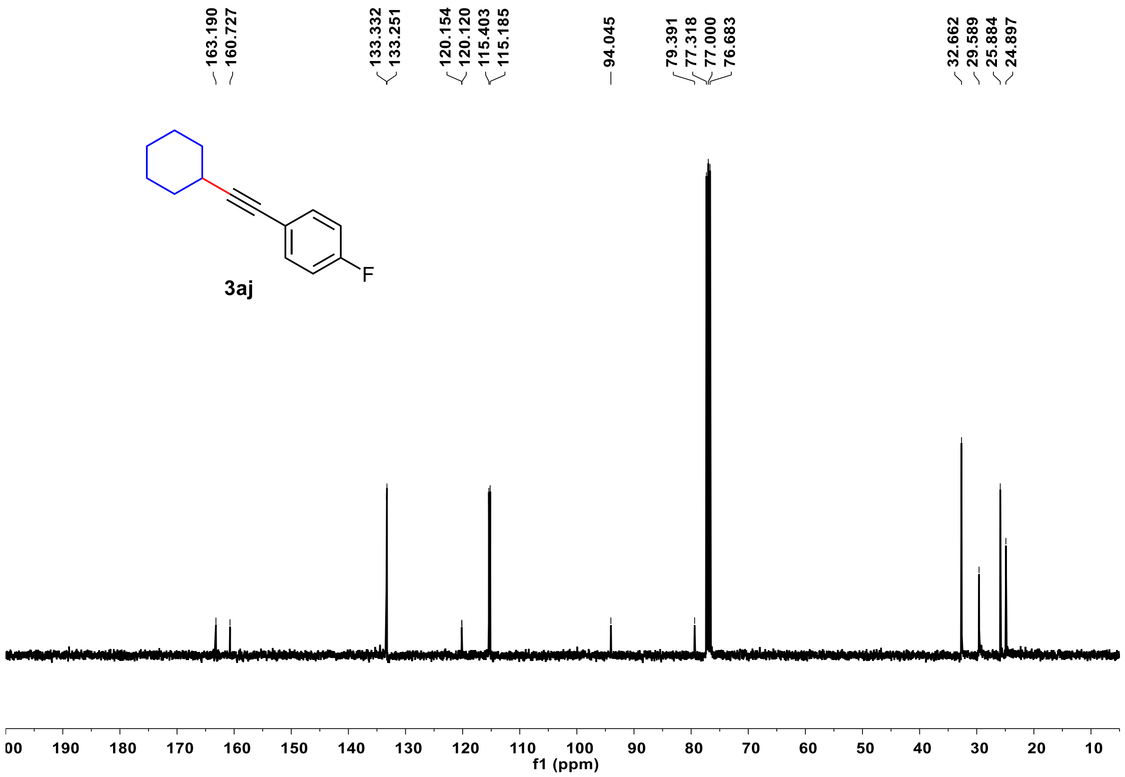

Supplementary Figure 42. ^{13}C NMR (101 MHz, $CDCl_3$) spectrum of 3aj

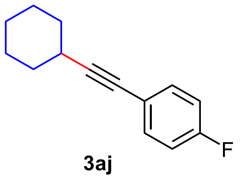

3aj

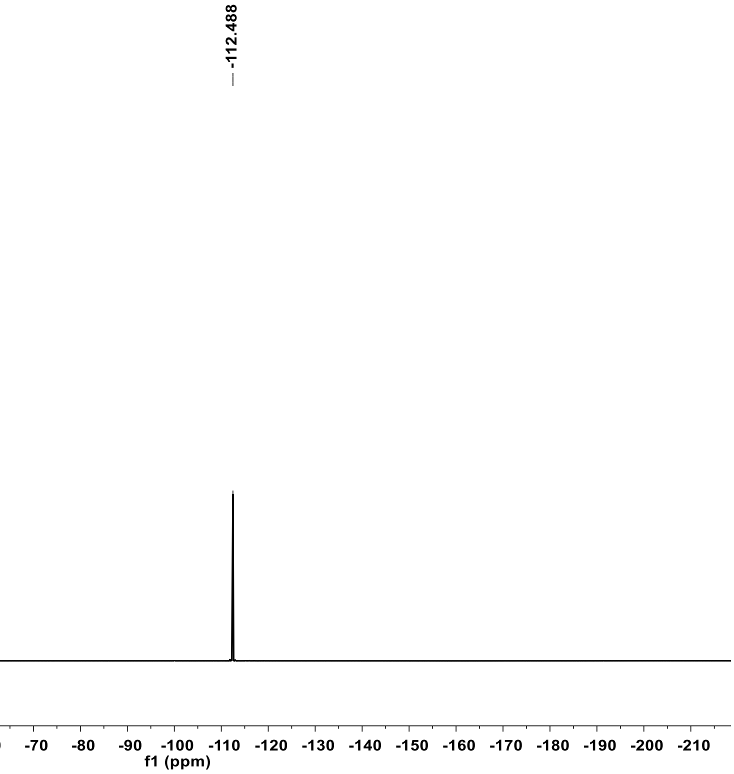

Supplementary Figure 43. ^{19}**F NMR (400 MHz,** $CDCl_3$**) spectrum of 3aj**

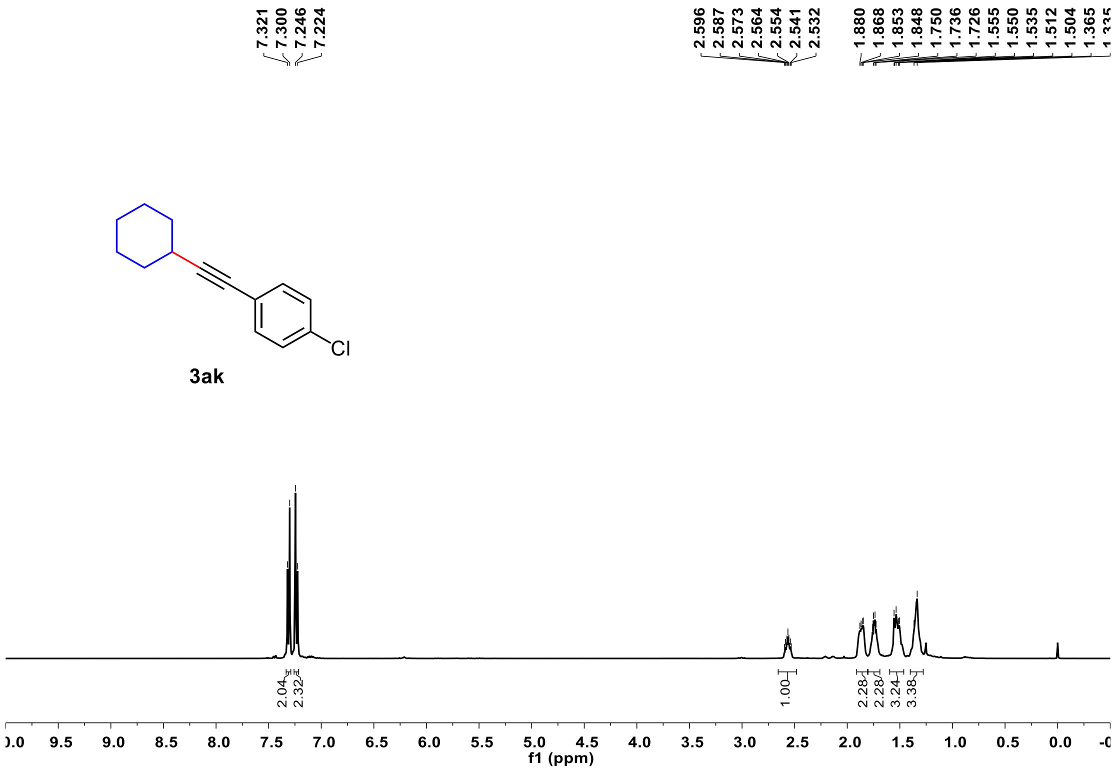

Supplementary Figure 44. **^{1}H NMR (400 MHz, $CDCl_3$) spectrum of 3ak**

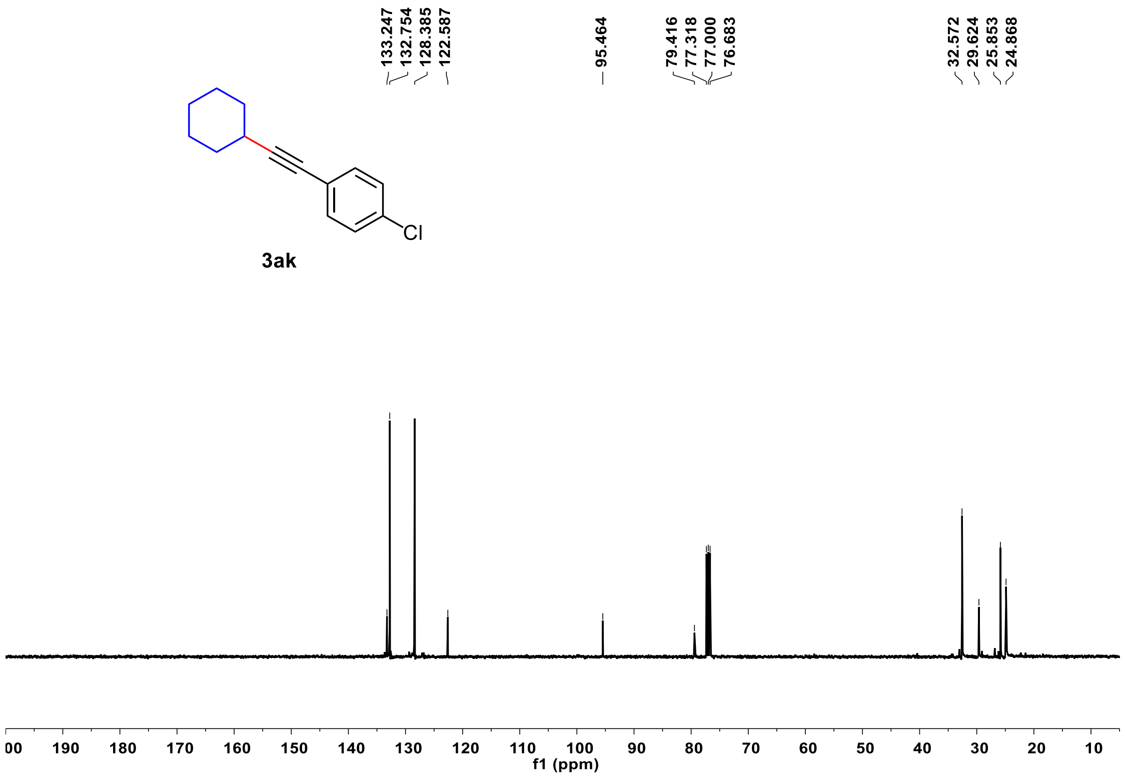

Supplementary Figure 45. **^{13}C NMR (101 MHz, $CDCl_3$) spectrum of 3ak**

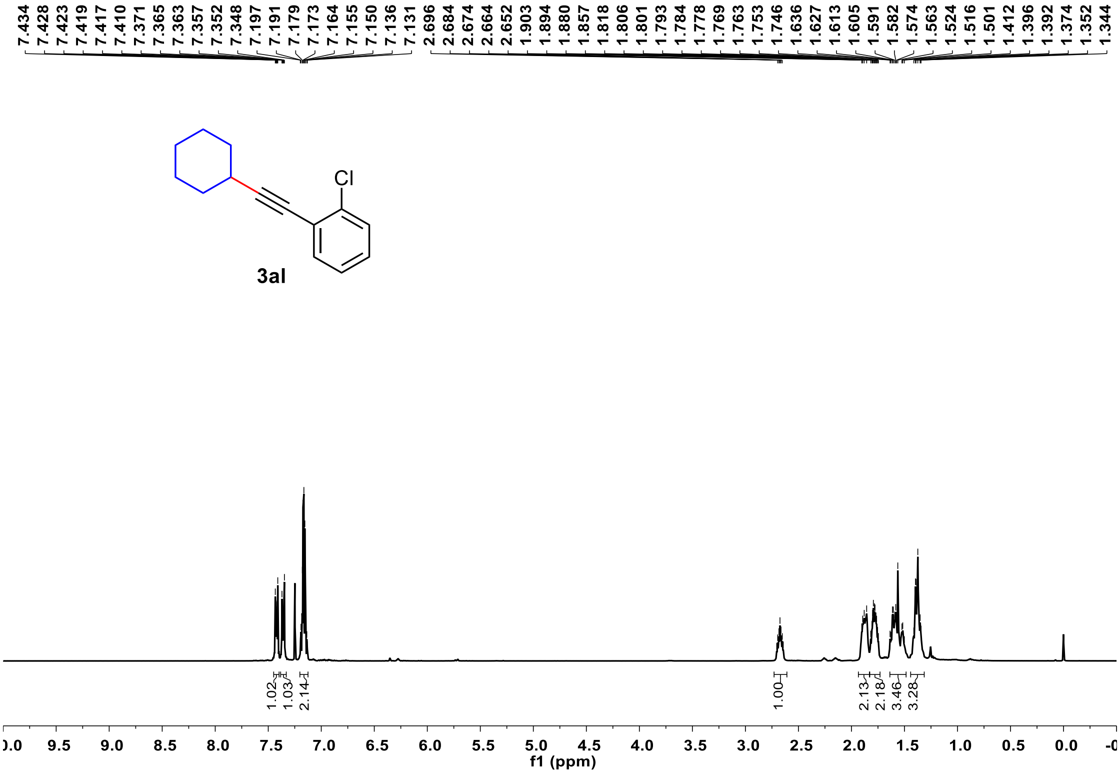

Supplementary Figure 46. ^{1}H NMR (400 MHz, $CDCl_3$) spectrum of 3al

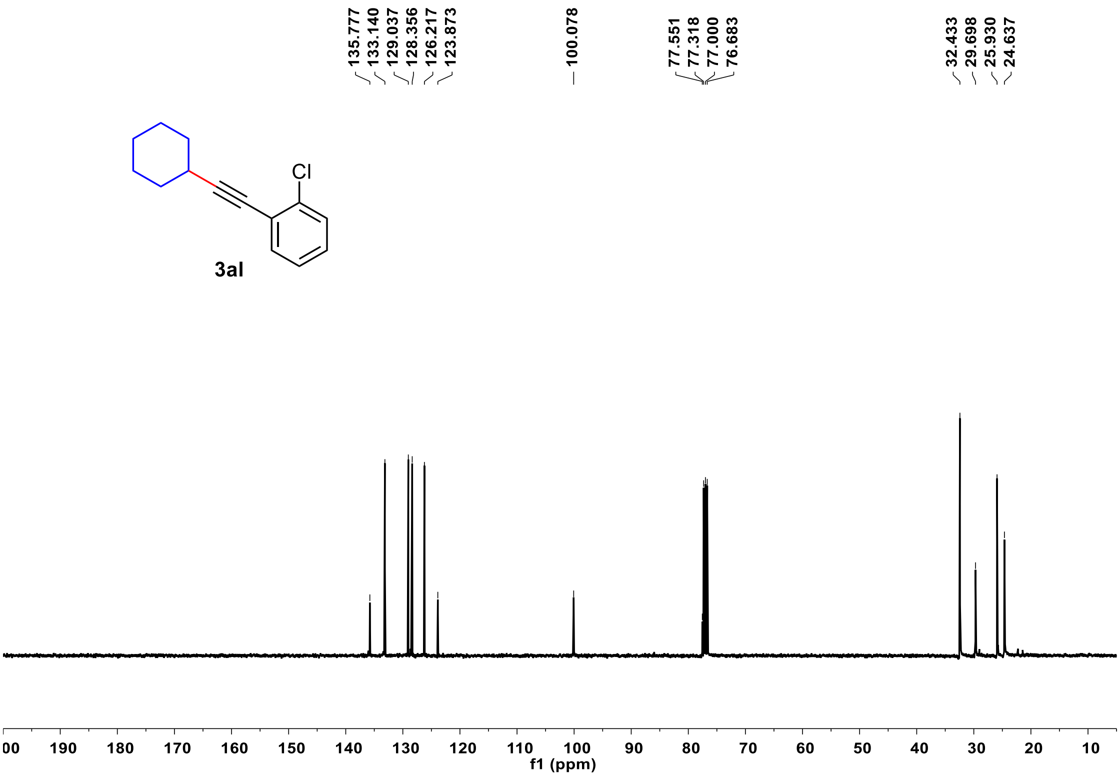

Supplementary Figure 47. ^{13}C NMR (101 MHz, $CDCl_3$) spectrum of 3al

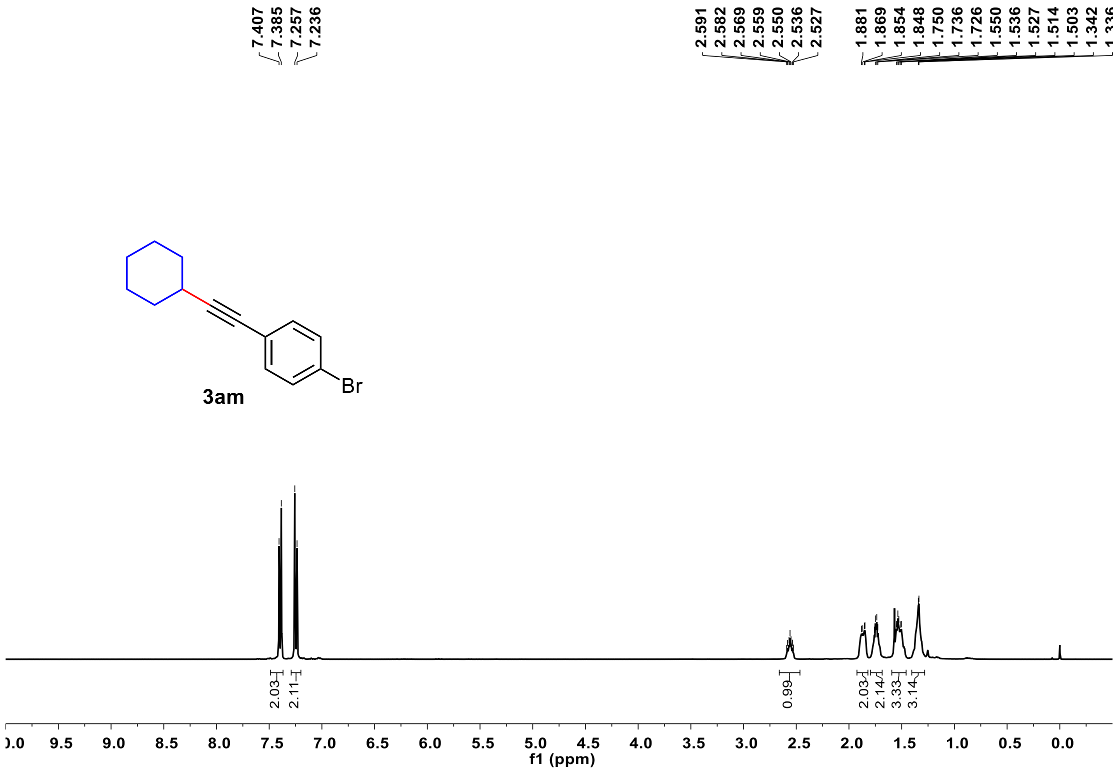

Supplementary Figure 48. ^{1}H NMR (400 MHz, $CDCl_3$) spectrum of 3am

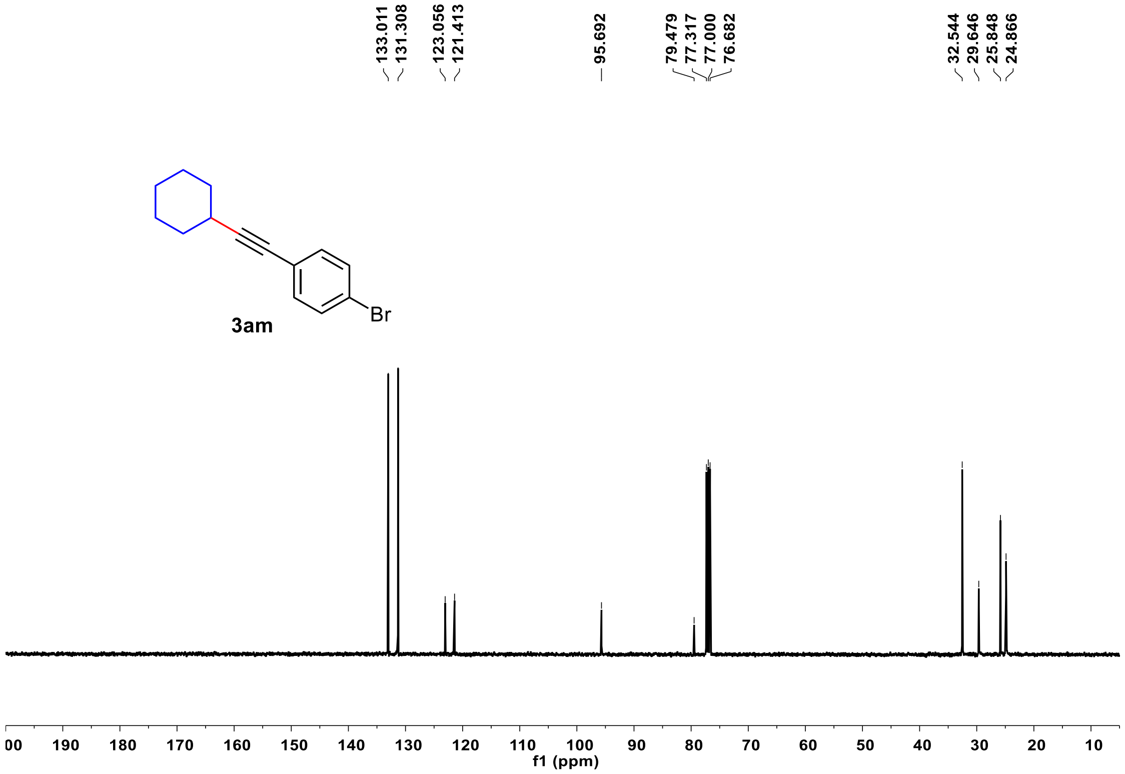

Supplementary Figure 49. ^{13}C NMR (101 MHz, $CDCl_3$) spectrum of 3am

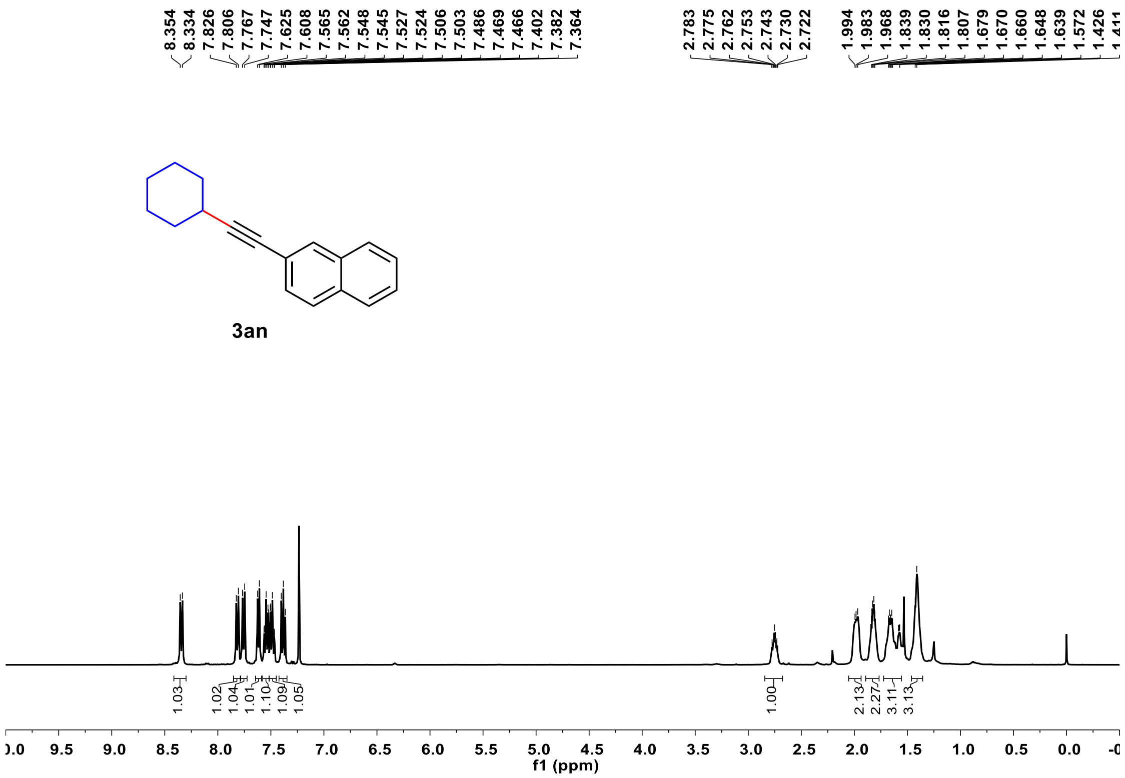

Supplementary Figure 50. ^{1}H **NMR (400 MHz,** $CDCl_3$**) spectrum of 3an**

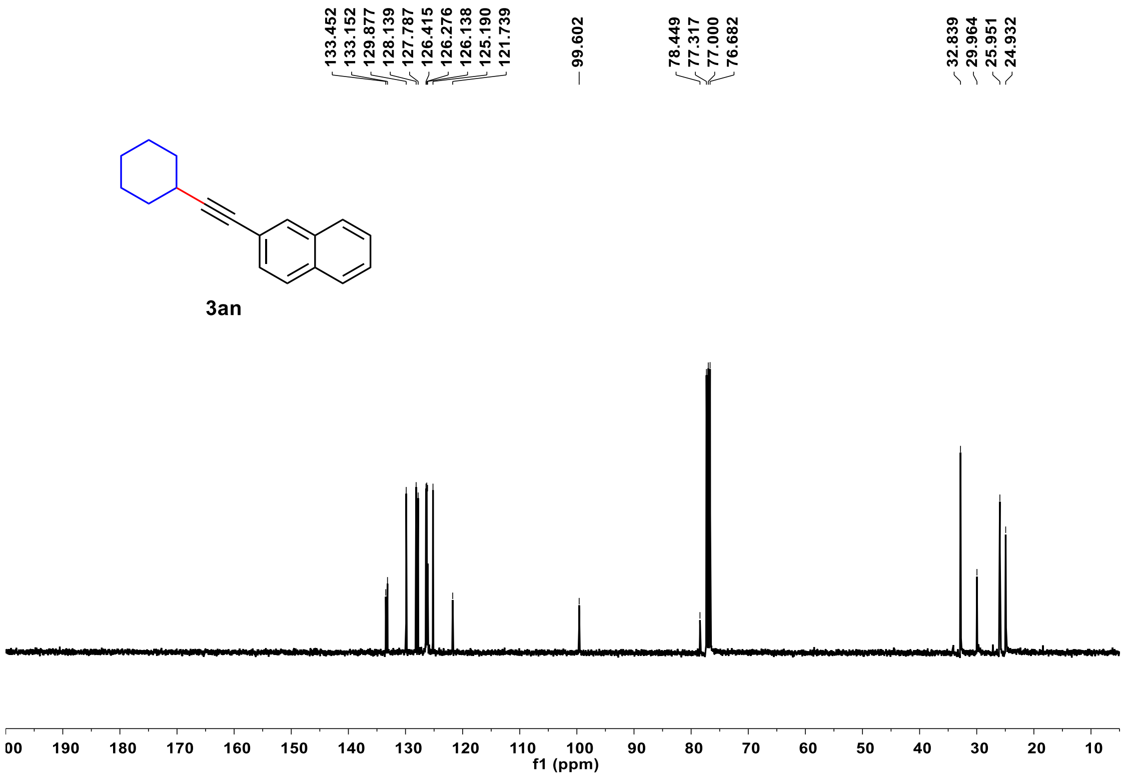

Supplementary Figure 51. ^{13}C **NMR (101 MHz,** $CDCl_3$**) spectrum of 3an**

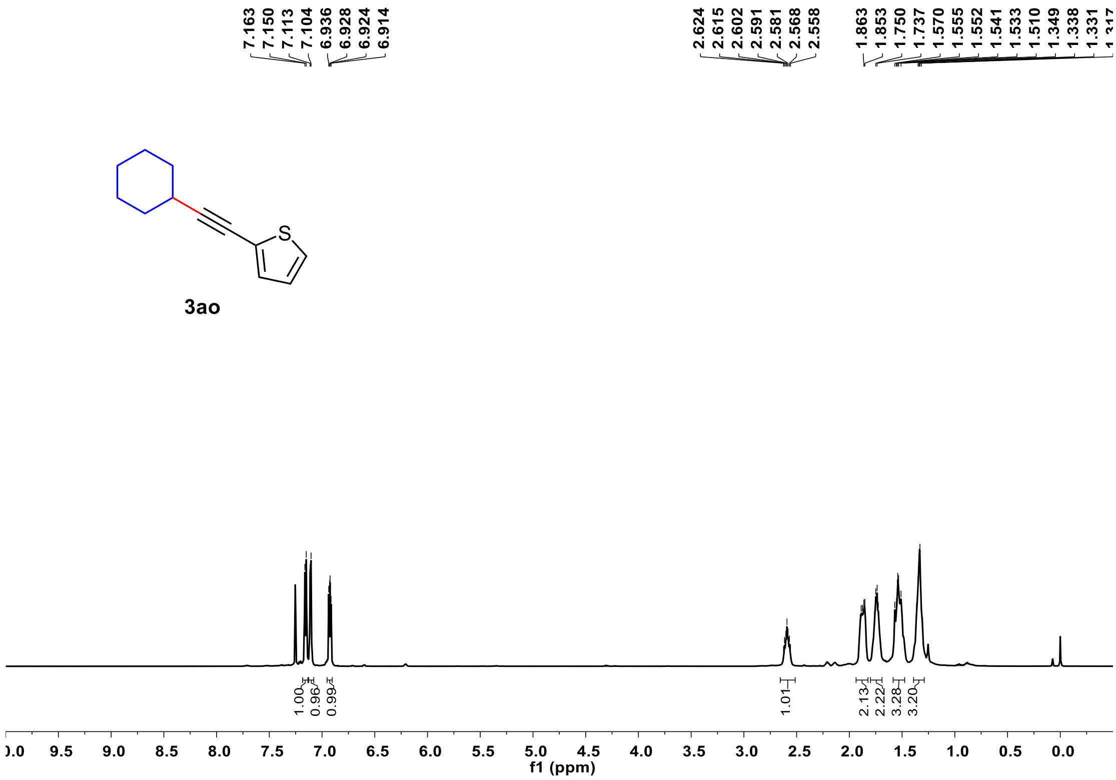

Supplementary Figure 52. ^{1}H **NMR (400 MHz,** $CDCl_3$**) spectrum of 3ao**

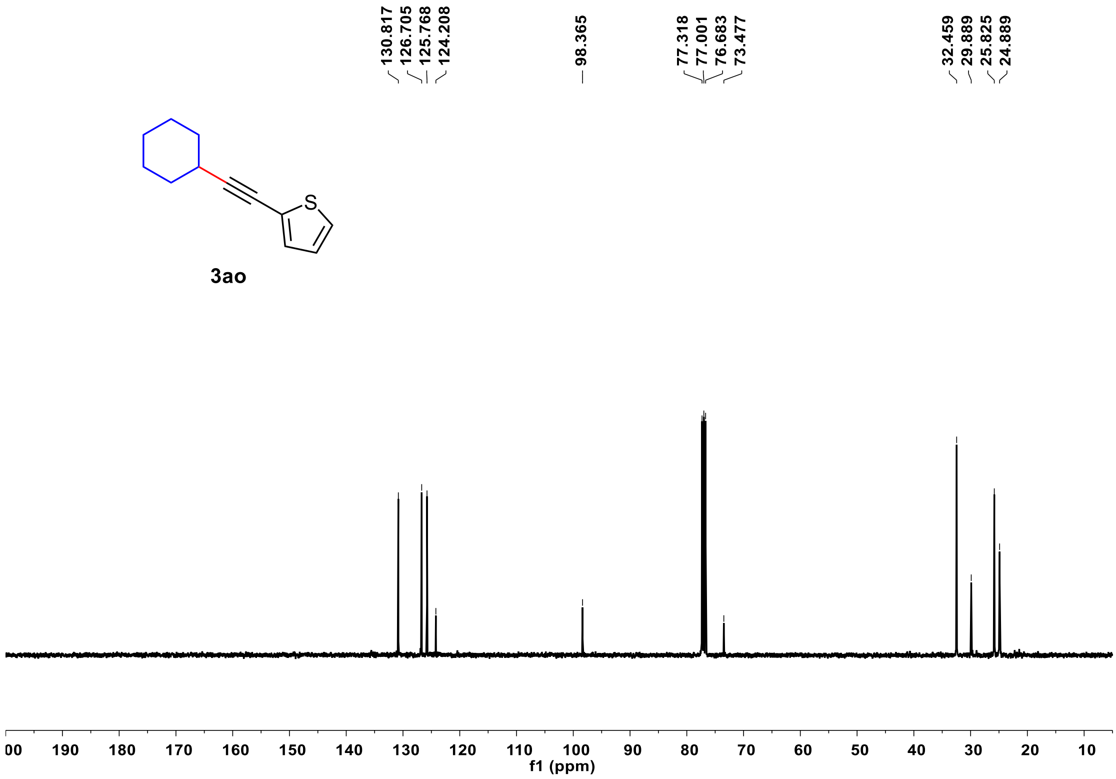

Supplementary Figure 53. ^{13}C **NMR (101 MHz,** $CDCl_3$**) spectrum of 3ao**

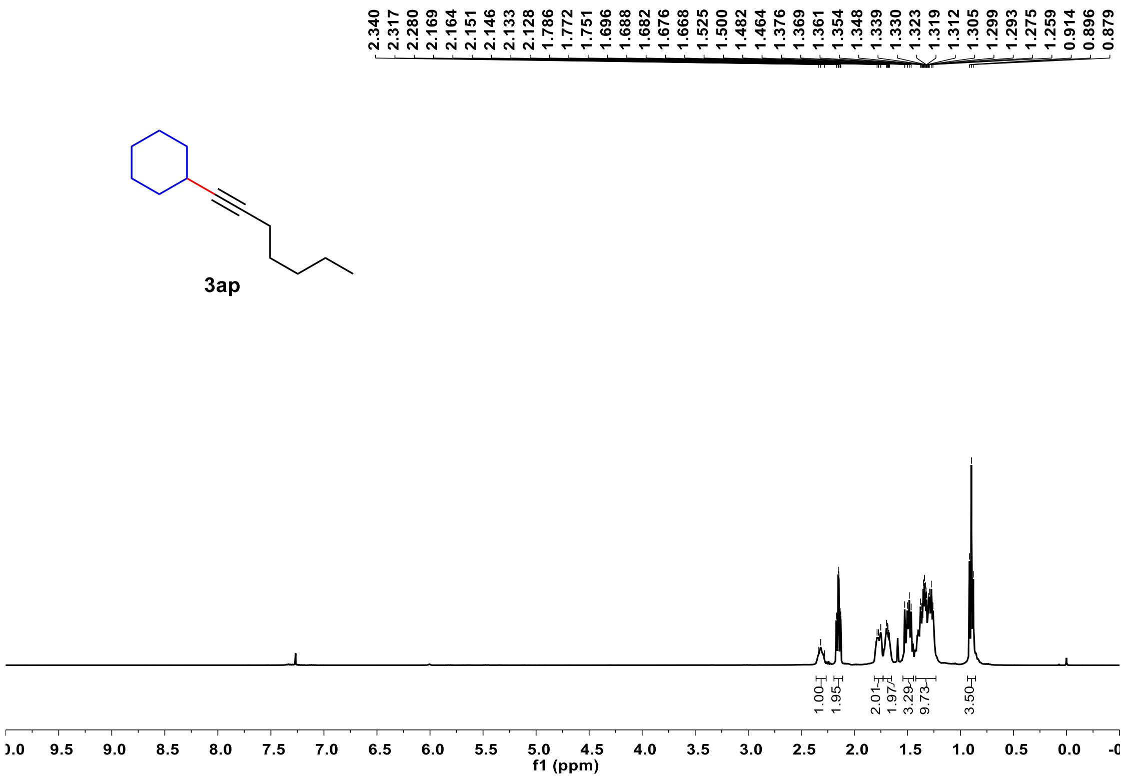

Supplementary Figure 54. ^{1}H NMR (400 MHz, $CDCl_3$) spectrum of 3ap

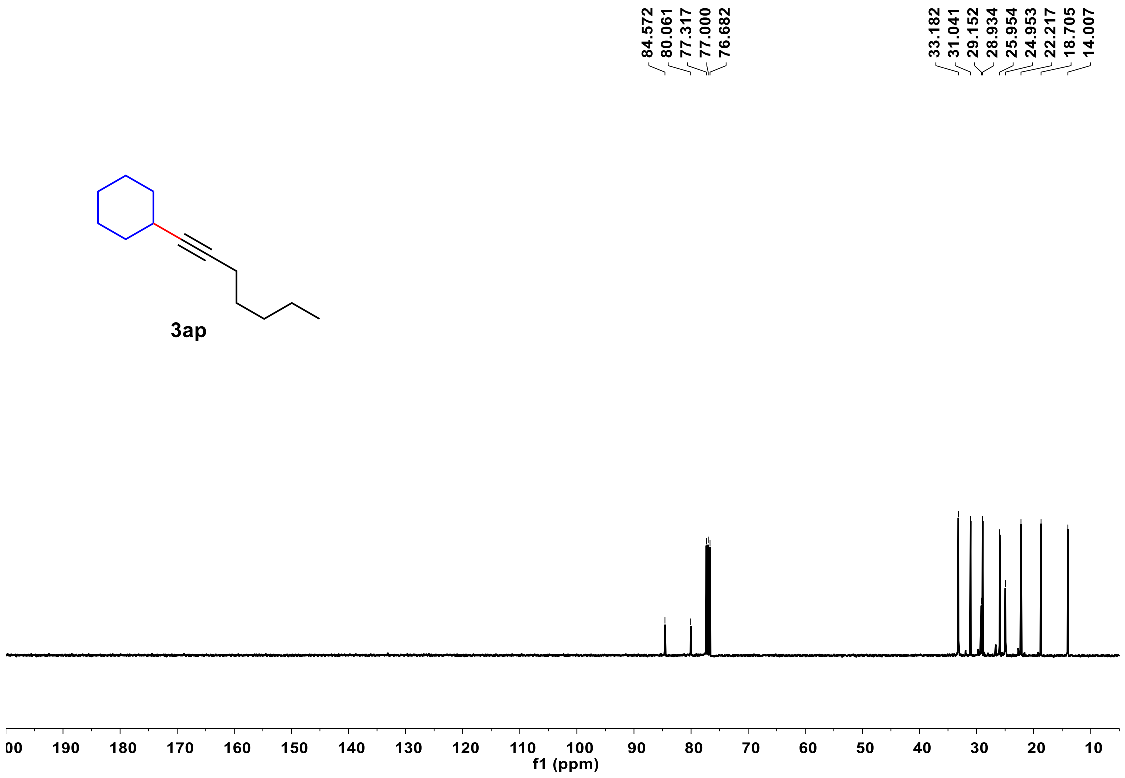

Supplementary Figure 55. ^{13}C NMR (101 MHz, $CDCl_3$) spectrum of 3ap

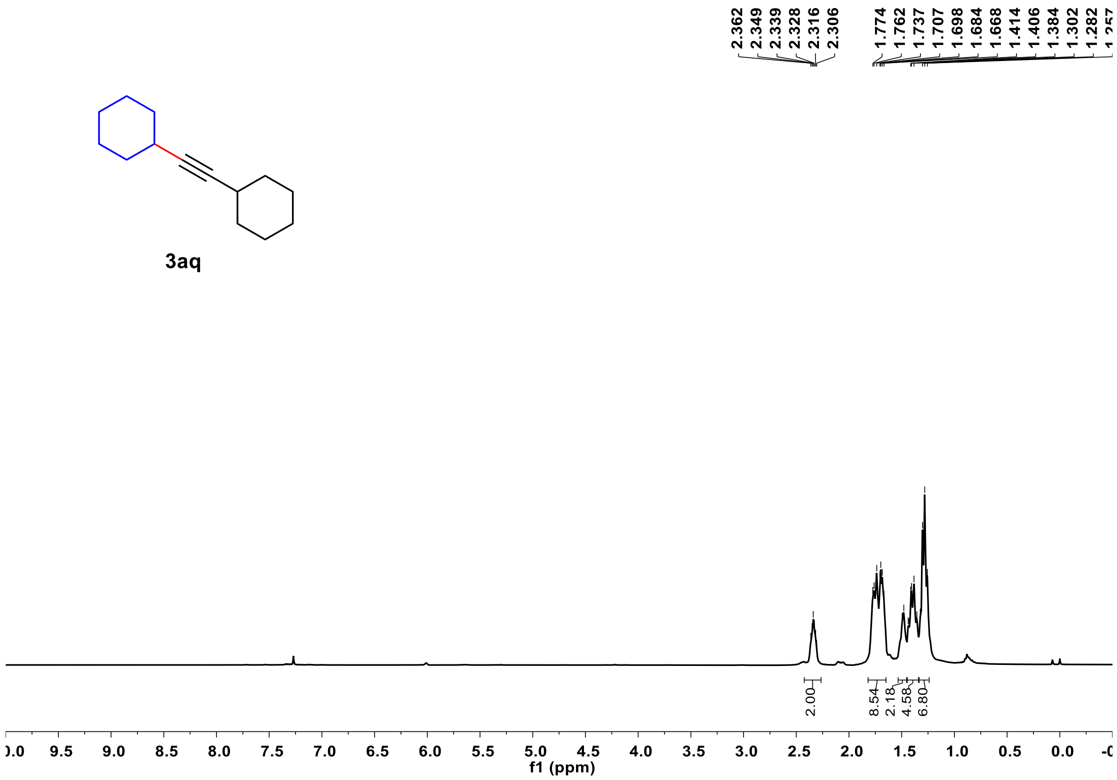

Supplementary Figure 56. ^{1}H NMR (400 MHz, $CDCl_3$) spectrum of 3aq

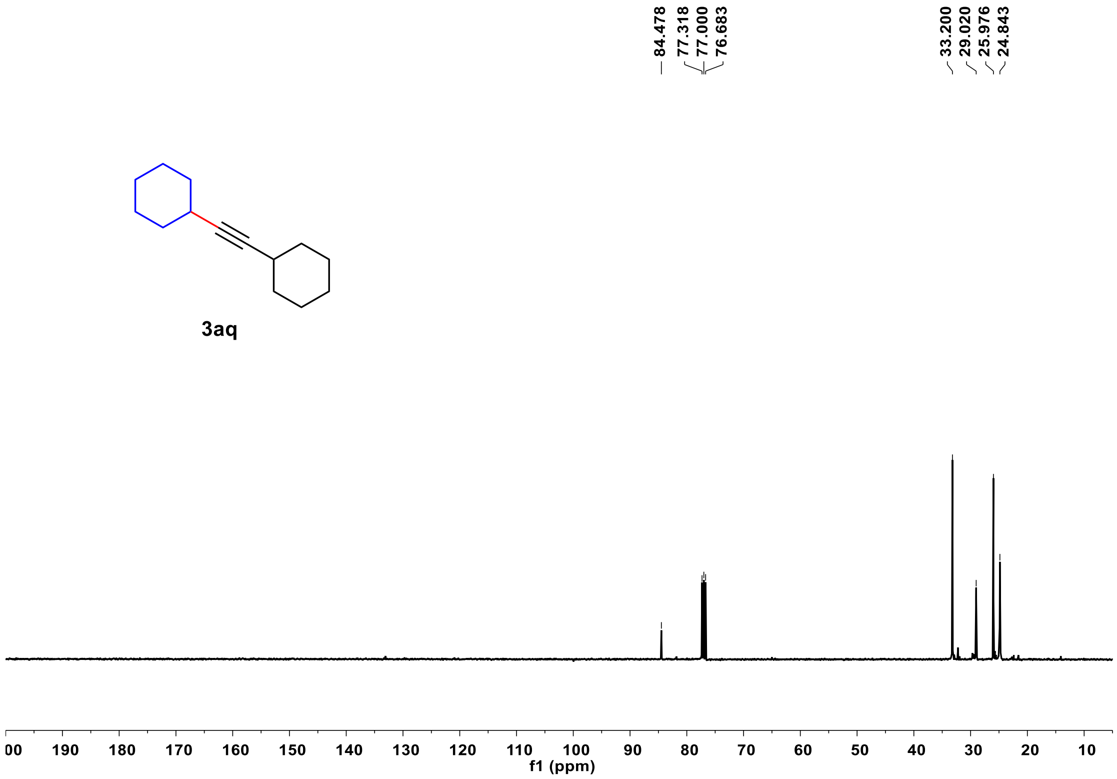

Supplementary Figure 57. ^{13}C NMR (101 MHz, $CDCl_3$) spectrum of 3aq

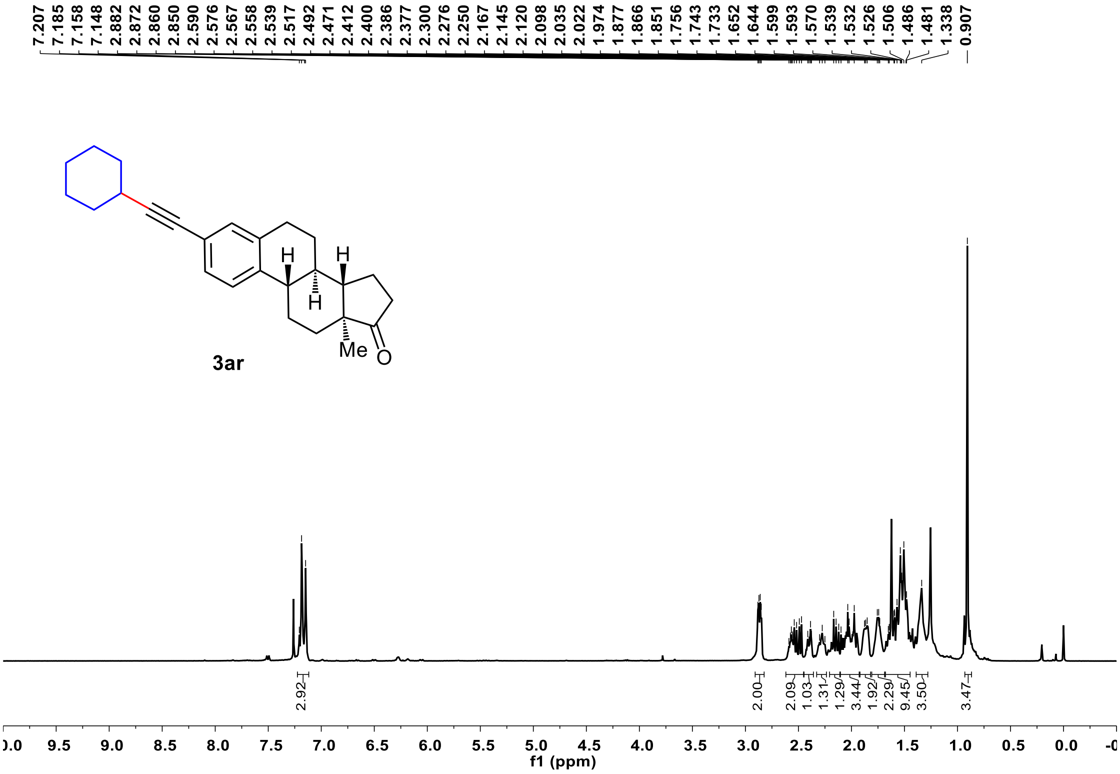

Supplementary Figure 58. ^{1}H NMR (400 MHz, $CDCl_3$) spectrum of 3ar

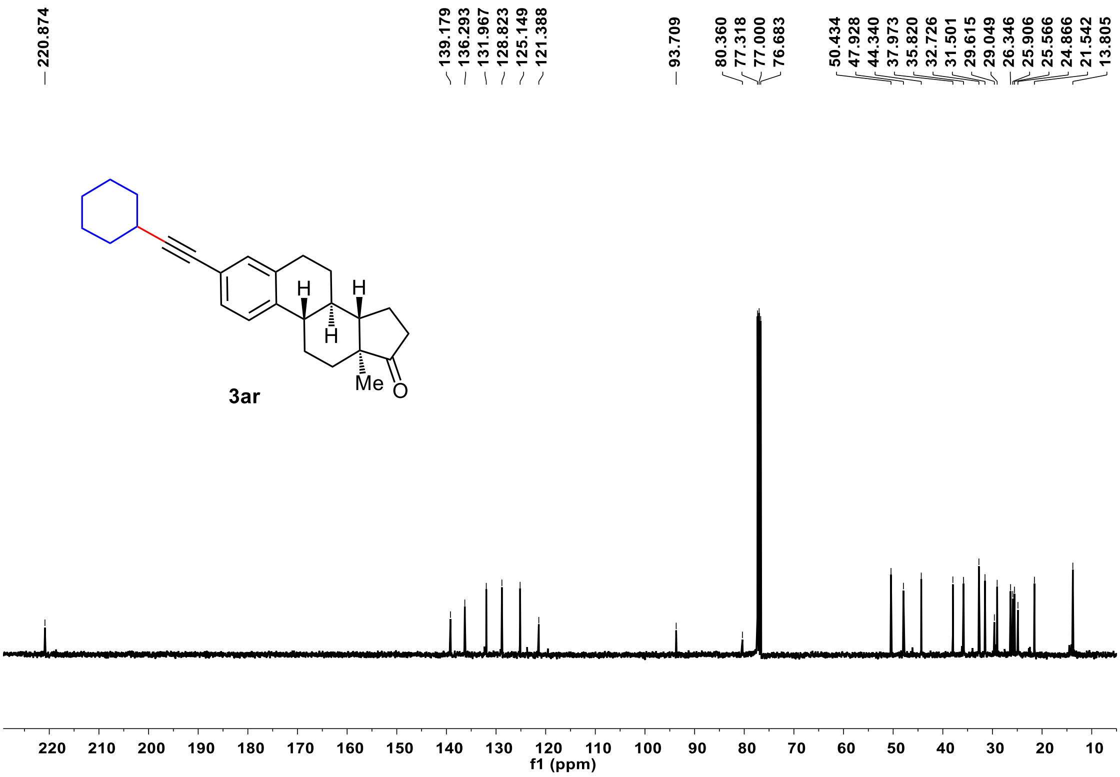

Supplementary Figure 59. ^{13}C NMR (101 MHz, $CDCl_3$) spectrum of 3ar

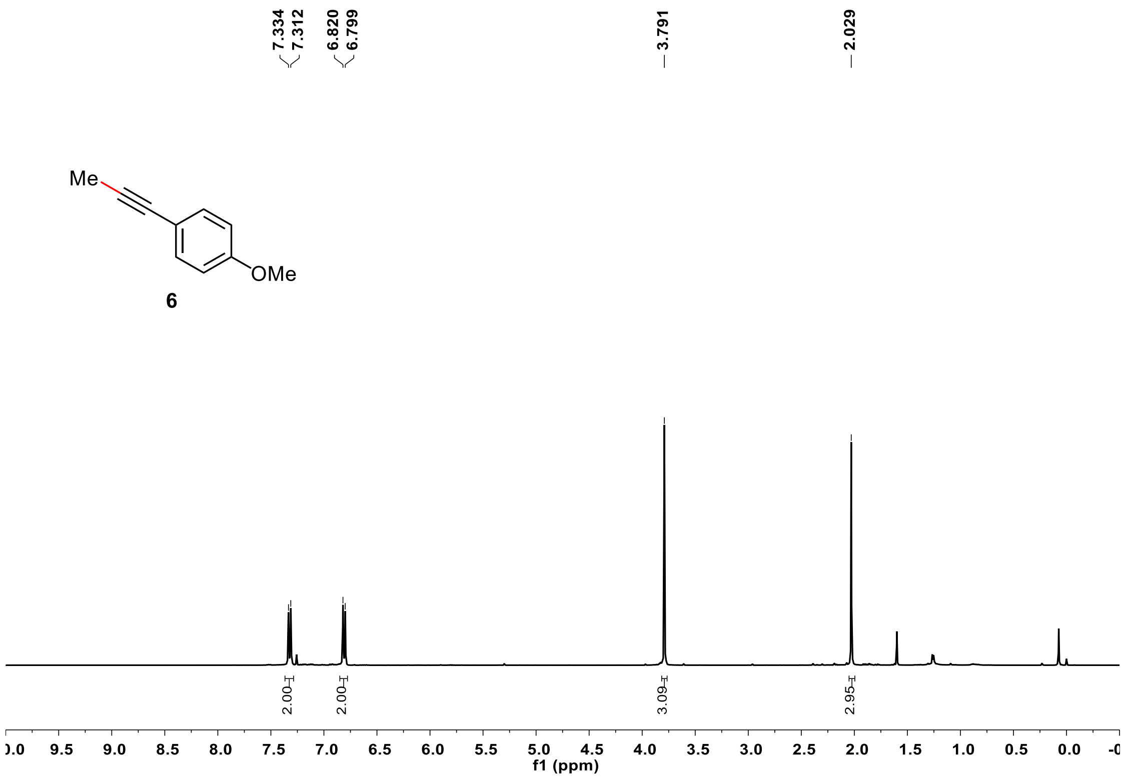

Supplementary Figure 60. ^{1}H NMR (400 MHz, $CDCl_3$) spectrum of 6

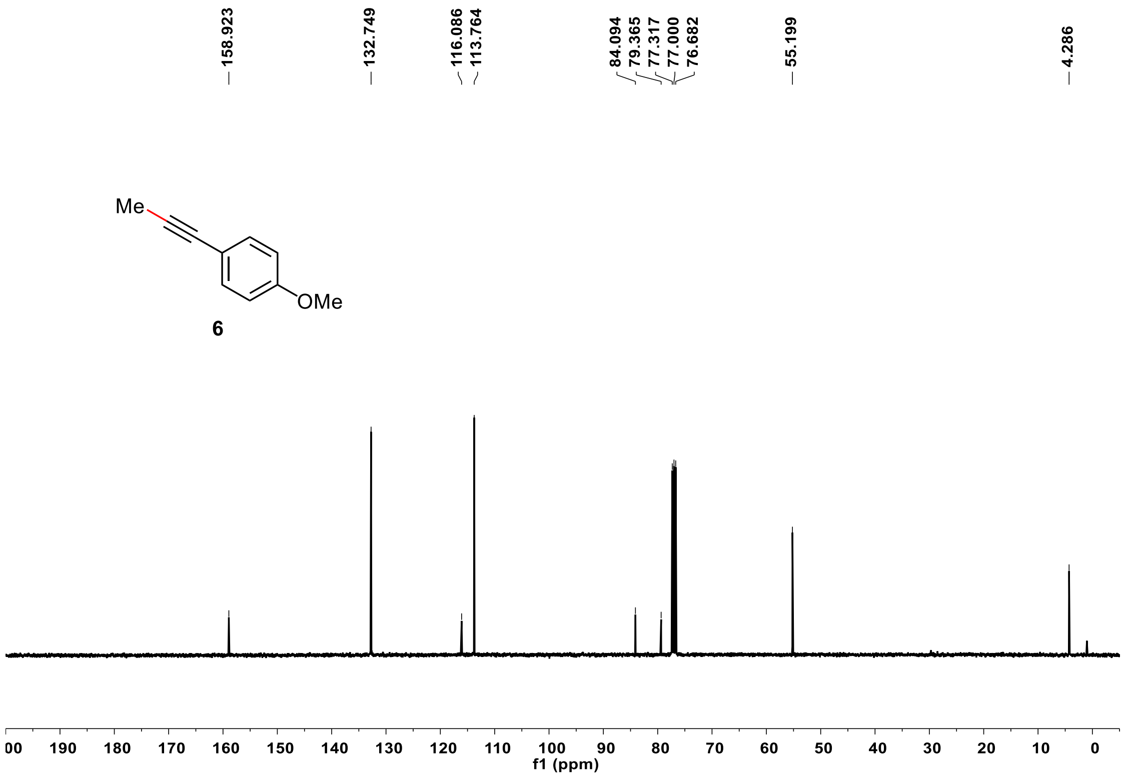

Supplementary Figure 61. ^{13}C NMR (101 MHz, $CDCl_3$) spectrum of 6

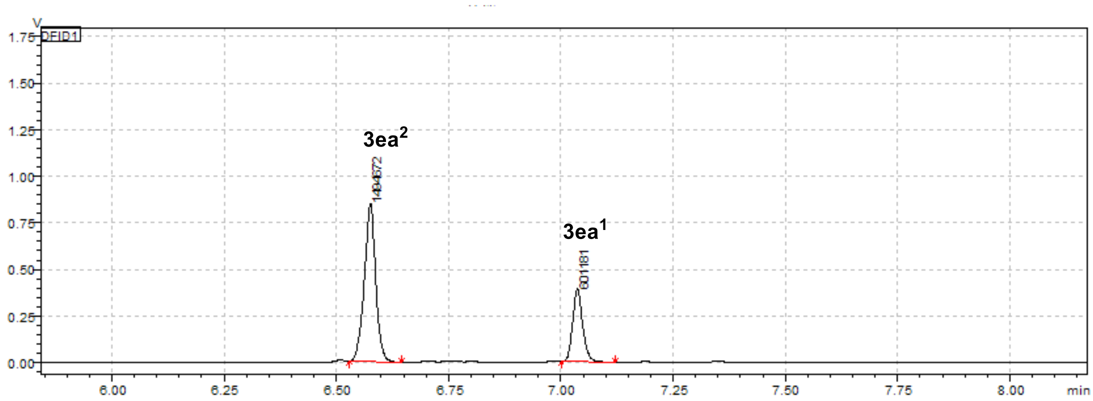

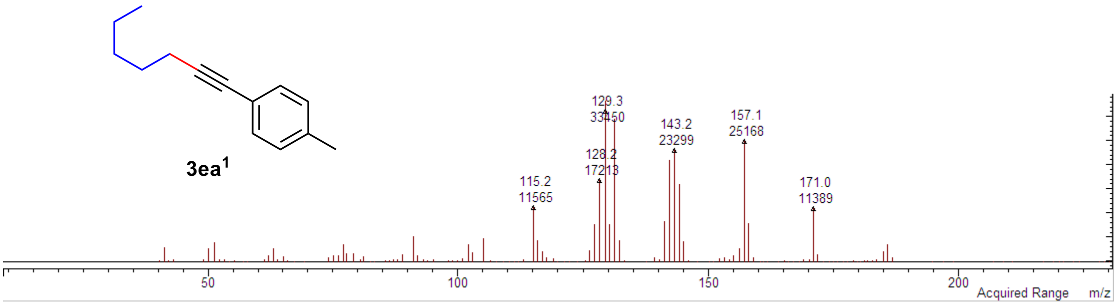

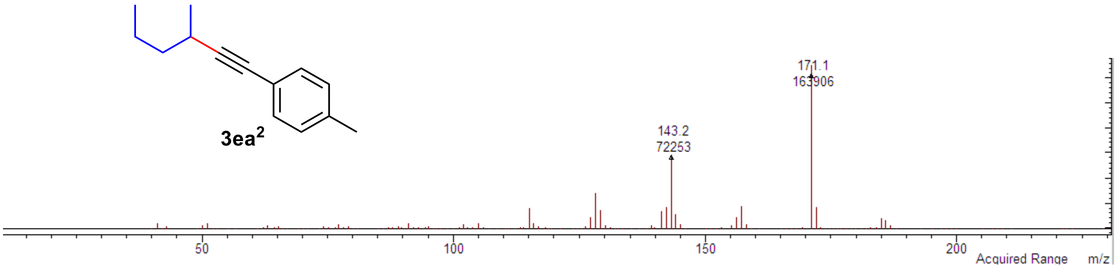

Supplementary Figure 62. GC-MS spectrum of 3ea

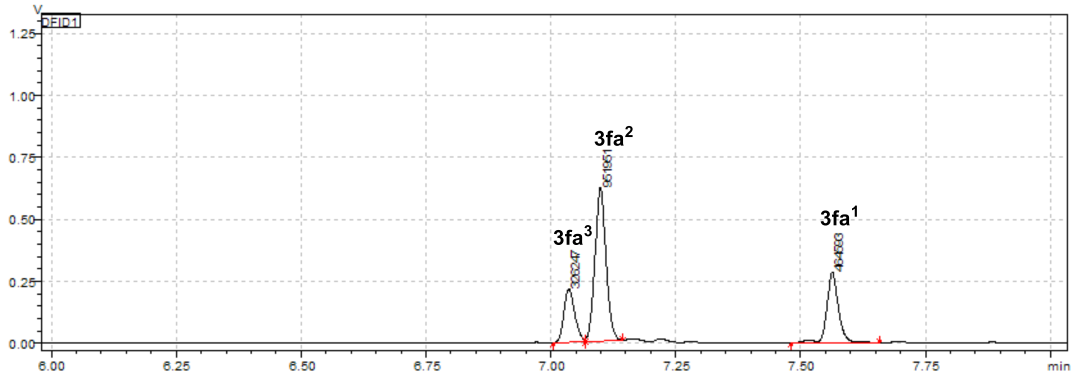

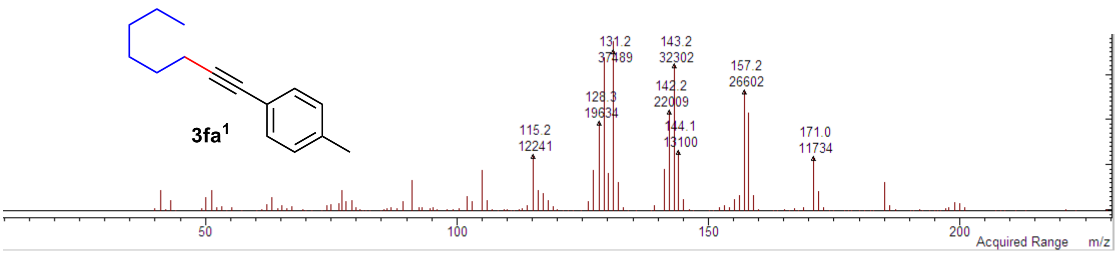

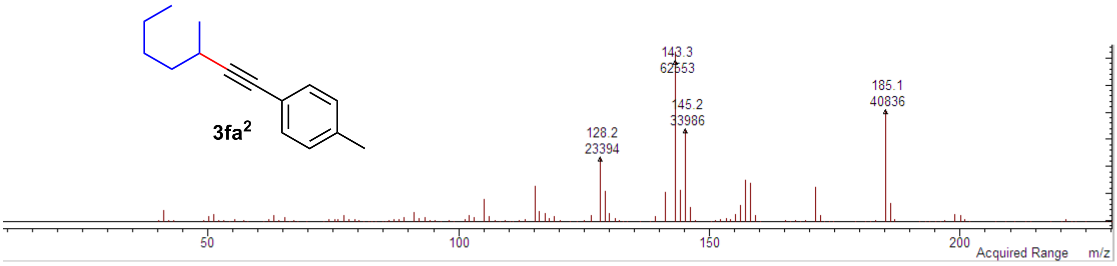

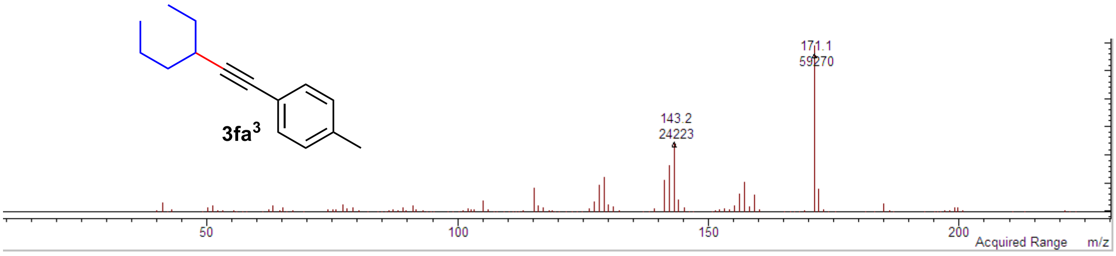

Supplementary Figure 63. GC-MS spectrum of 3fa

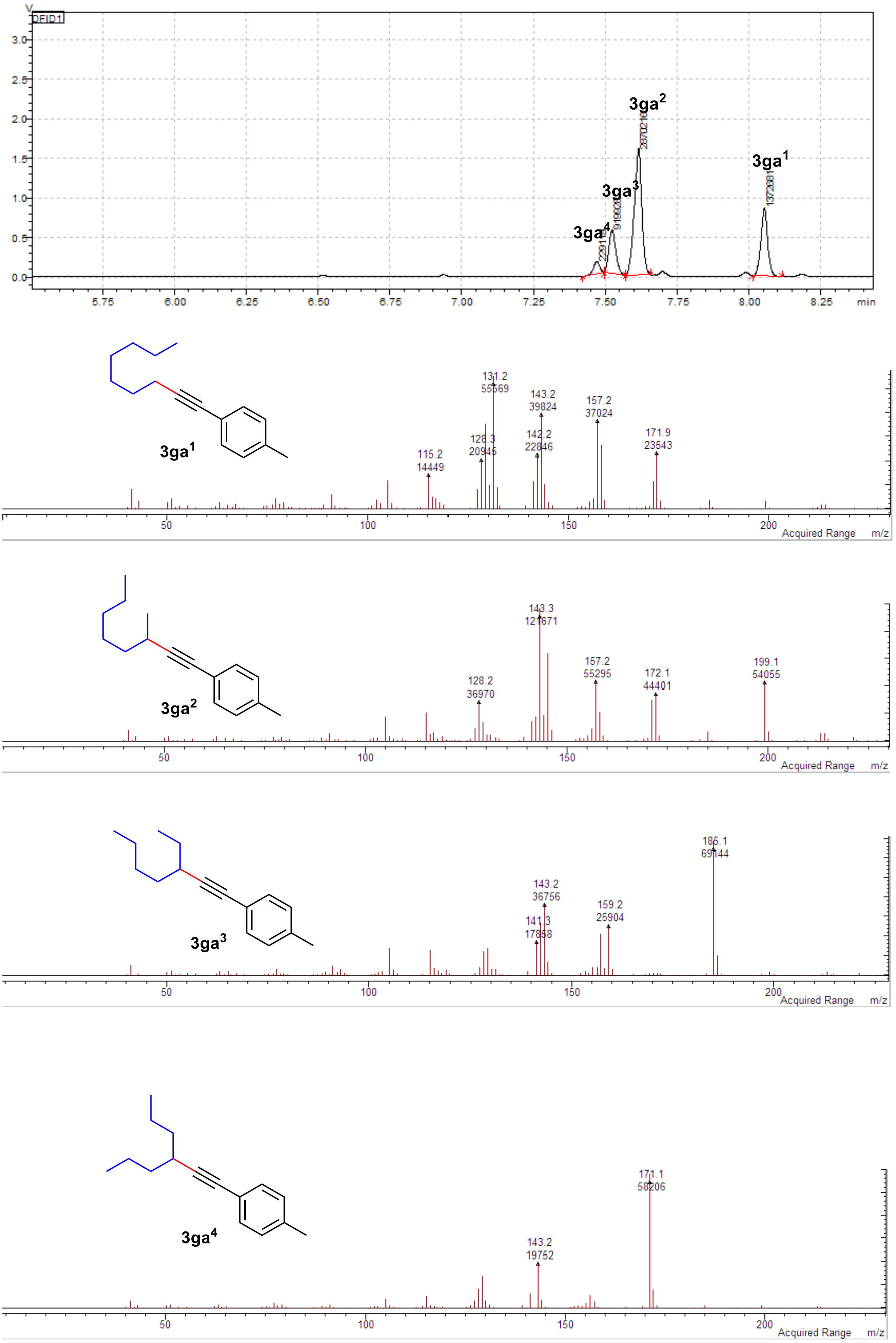

Supplementary Figure 64. GC-MS spectrum of 3ga

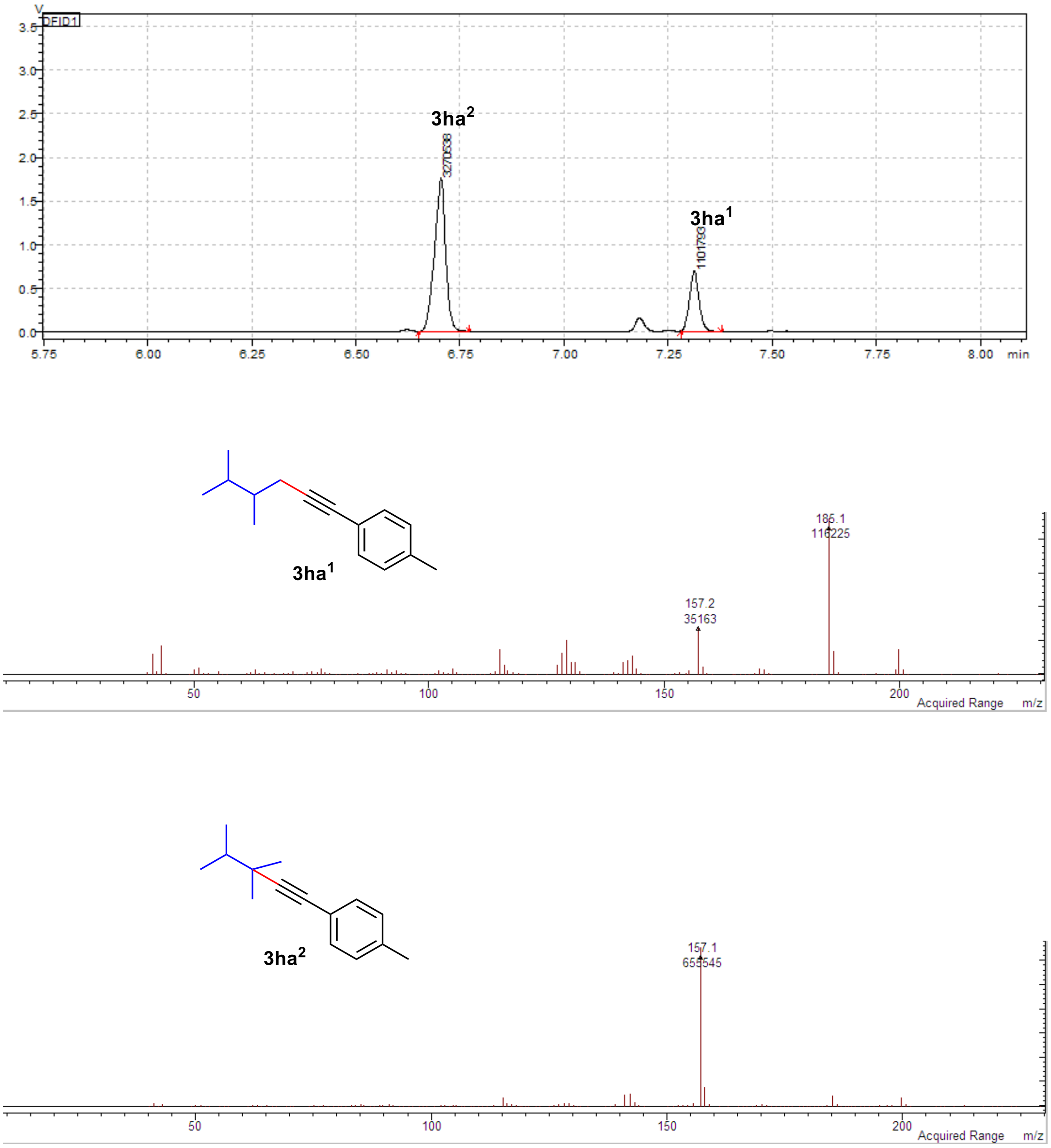

Supplementary Figure 65. GC-MS spectrum of 3ha

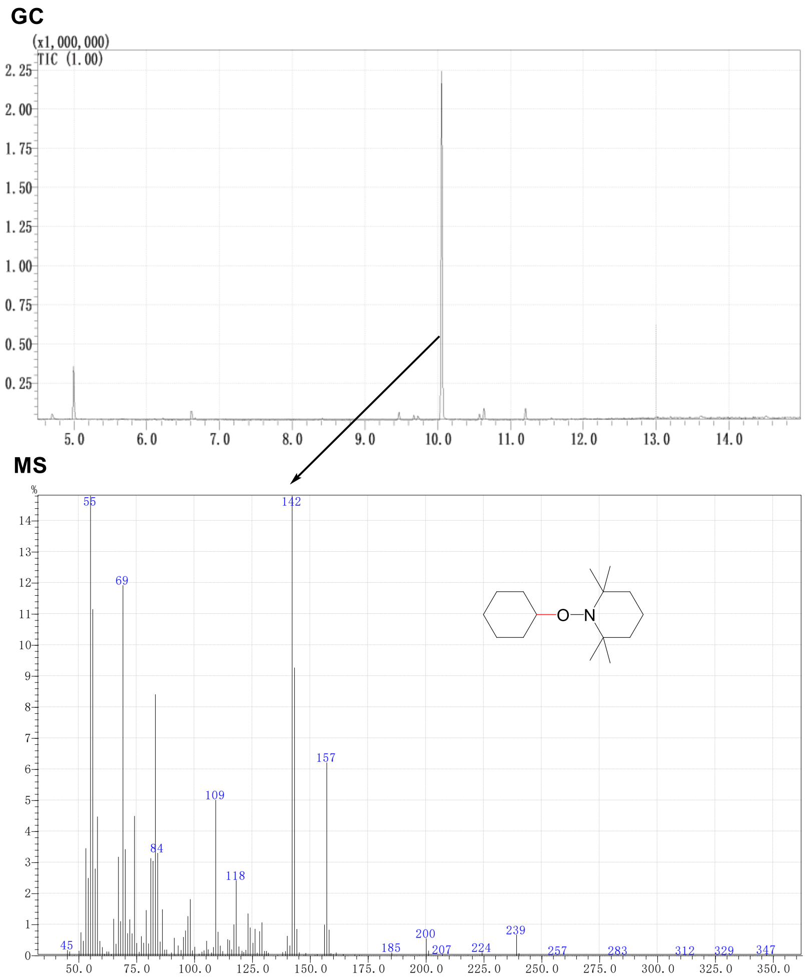

Supplementary Figure 66. GC-MS spectrum of radical trapping product 7

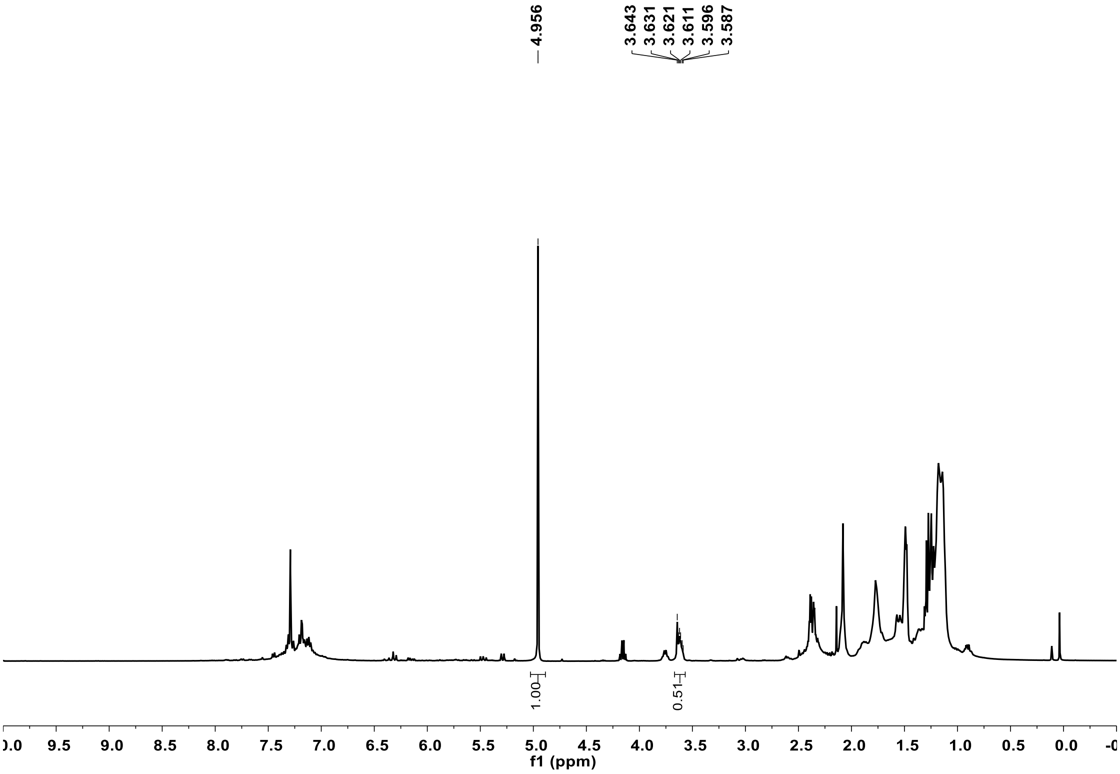

Supplementary Figure 67. Crude NMR of the radical trapping experiment.

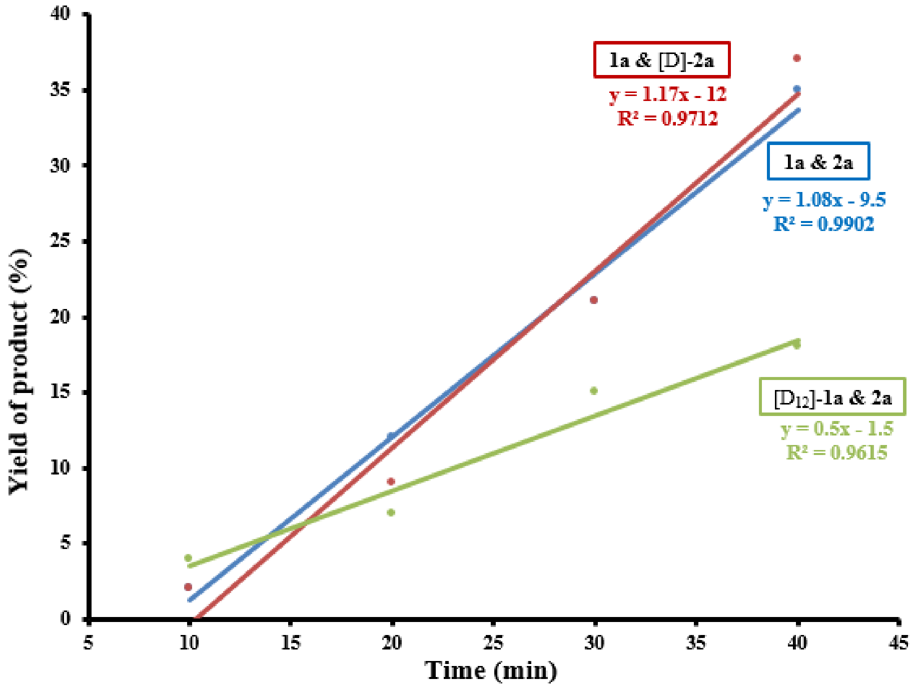

Supplementary Figure 68. Plot of initial rates. The initial reaction rate of **1a** with **2a**, [D_{12}]–**1a** with **2a**, **1a** with [D]–**2a**.

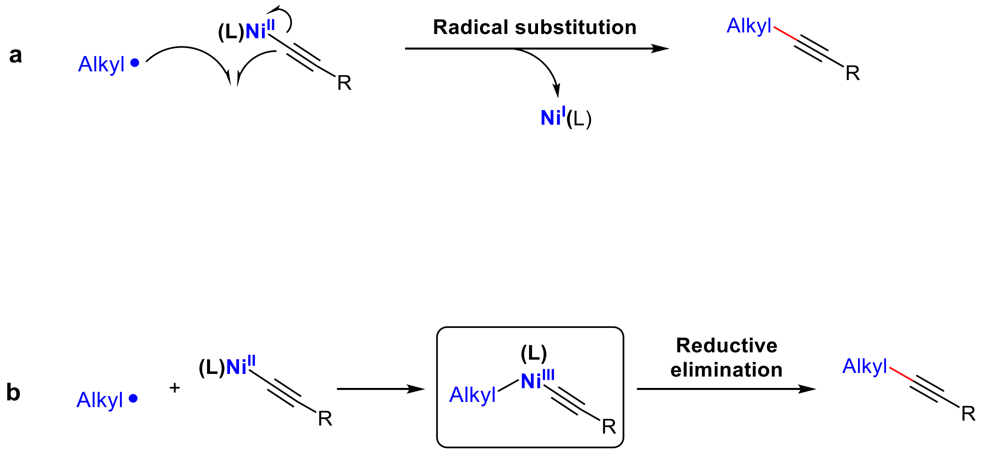

Supplementary Figure 69. Possible reaction pathways for C(sp^3)-C(sp) bond formation. (**a**) Radical homolytic substitution pathway. (**b**) Reductive elimination pathway

Supplementary Tables

Supplementary Table 1. Screening of co-catalysts

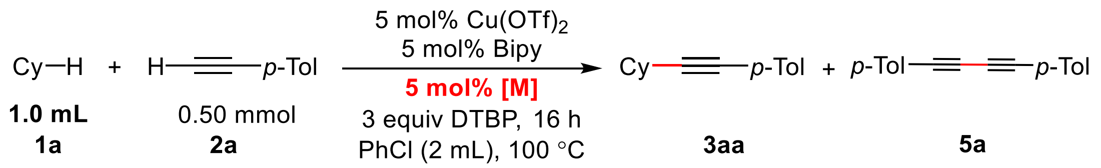

Entry	[M]	Conversion of **2a** (%)	GC Yield (%)	
			**3aa**	**5a**
1	$Ni(acac)_2$	92	**24**	22
2	$Cu(acac)_2$	95	15	41
3	$Co(acac)_2$	82	15	9
4	$Fe(acac)_2$	77	21	n.d.

Supplementary Table 2. Screening of concentrations

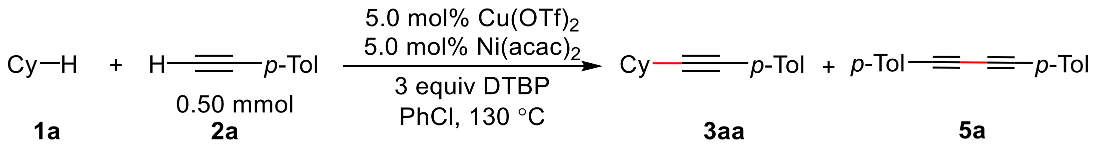

Entry	CyH/PhCl	Conversion of **2a** (%)	GC Yield (%)	
			**3aa**	**5a**
1	3.0 mL / 1.0 mL	100	39	4
2	2.0 mL / 2.0 mL	100	45	14
3	3.0 mL / 2.0 mL	100	48	13
4	4.0 mL / 2.0 mL	100	54	12
5	2.0 mL / 3.0 mL	100	37	13
6	3.0 mL / 3.0 mL	100	44	14
7	4.0 mL / 3.0 mL	100	**57**	10

Supplementary Table 3. Screening of phosphine ligands

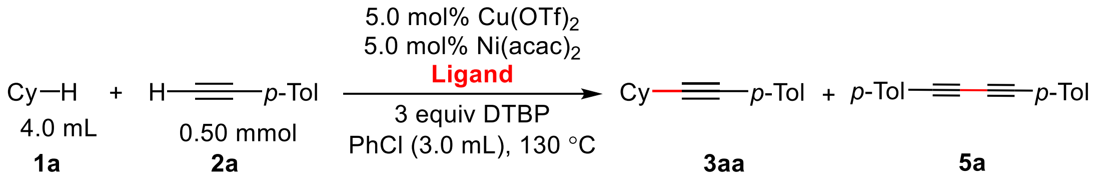

Entry	Ligand	GC Yield (%)	
		**3aa**	**5a**
1	none	51	13
2	10 mol% PPh_3	57	12
3	5.0 mol% dppm	25	12
4	5.0 mol% dppe	56	6
5	5.0 mol% dppp	59	7
6	5.0 mol% dppb	**59**	5
7	5.0 mol% dpph	55	9
8	5.0 mol% DPEphos	15	5
9	5.0 mol% Xantphos	13	4

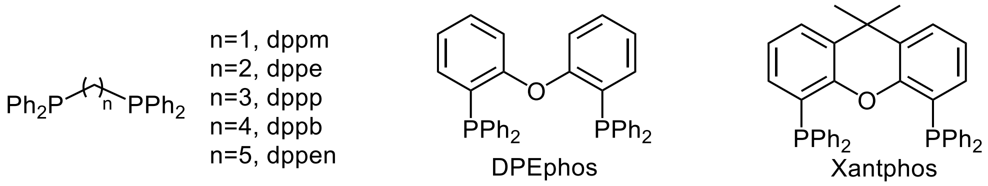

Supplementary Table 4. Effect of the catalyst ratio

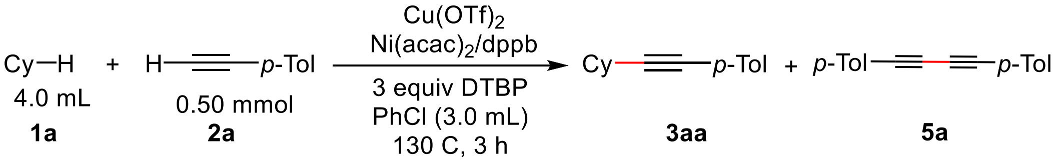

Entry	$Cu(OTf)_2$	$Ni(acac)_2$/dppb	GC Yield (%)	
			**3aa**	**5a**
1	7.5 mol%	5.0 mol%	58	8
2	5.0 mol%	7.5 mol%	5	4
3	7.5 mol%	7.5 mol%	**63**	8
4	none	5.0 mol%	n.d.	3
5	5.0 mol%	none	14	9

Supplementary Table 5. Screening of silver co-catalysts

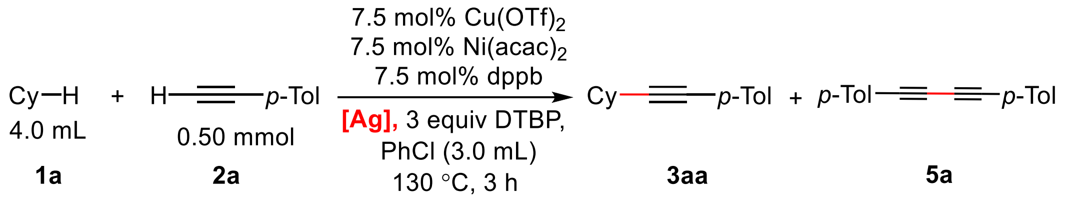

Entry	[Ag]	GC Yield (%)	
		**3aa**	**5a**
1	7.5 mol% AgOAc	72	3
2	7.5 mol% AgOPiv	58	11
3	7.5 mol% $AgBF_4$	68	6
4	5 mol% AgOAc	66	5
5	10 mol% AgOAc	**75**	trace
6	12.5 mol% AgOAc	73	trace
7	15 mol% AgOAc	65	trace

Supplementary Methods

General information: All glassware was oven dried at 110 °C for hours and cooled down under vacuum. **2r**[1], (phenylethynyl)copper (**2d-[Cu]**)[2] and (phenylethynyl)silver (**2d-[Ag]**)[3] were synthesized according to literature reports. Unless otherwise noted, materials were obtained from commercial suppliers and used without further purification. Thin layer chromatography (TLC) employed glass 0.25 mm silica gel plates. Flash chromatography columns were packed with 200-300 mesh silica gel in petroleum (bp. 60-90 °C). Gas chromatographic analyses were performed on SHIMADZU GC-2014 gas chromatography instrument with a FID detector and naphthalene was added as internal standard. GC-MS spectra were recorded on Varian GC MS 3900-2100T or SHIMADZU GC MS-2010. High resolution mass spectra (HRMS) were measured with a Waters Micromass GCT Premier. ^{1}H and ^{13}C NMR data were recorded with Bruker Advance III (400 MHz) spectrometers with tetramethylsilane as an internal standard. All chemical shifts (δ) are reported in ppm and coupling constants (*J*) in Hz. All chemical shifts are reported relative to tetramethylsilane and d-solvent peaks (77.00 ppm, chloroform), respectively.

Reaction conditions A: In an oven-dried Teflon septum screw-capped tube equipped with a stir bar, $Cu(OTf)_2$ (13.6 mg, 0.038 mmol), $Ni(acac)_2$ (9.6 mg, 0.038 mmol), dppb (16.0 mg, 0.038 mmol) and AgOAc (8.3 mg, 0.050 mmol) were combined and sealed. The tube was then charged with nitrogen. Then alkane (4.0 mL) and PhCl (3.0 mL) were injected into the tube by syringe. After stirring for 5 min, DTBP (1.5 mmol or 2.0 mmol) and terminal alkyne (0.50 mmol) were subsequently injected into the reaction tube. The reaction was then heated to 130 °C. After stirring for 3 h, the reaction was cooled down to room temperature and quenched with saturated $Na_2S_2O_3$ solution. After extraction with ethyl acetate (3 x 10 mL), the organic layers were combined and dried over anhydrous Na_2SO_4, the pure product was obtained by flash column chromatography on silica gel (petroleum: ethyl ether = 10:1).

Reaction conditions B: In an oven-dried Teflon septum screw-capped tube equipped with a stir bar, $Cu(OTf)_2$ (16.3 mg, 0.050 mmol), $Ni(acac)_2$ (12.9 mg, 0.050 mmol), dppb (21.3 mg, 0.050 mmol) and AgOAc (4.1 mg, 0.025 mmol) were combined and sealed. The tube was then charged with

nitrogen. Then toluene derivatives (7 mL) were injected into the tube by syringe. After stirring for 5 min, DTBP (109.5, 0.75 mmol) and terminal alkyne (0.50 mmol) were subsequently injected into the reaction tube. The reaction was then heated to 130 ^{o}C. After stirring for 3 h, the reaction was cooled down to room temperature and quenched with saturated $Na_2S_2O_3$ solution. After extraction with ethyl acetate (3 x 10 mL), the organic layers were combined and dried over anhydrous Na_2SO_4, the pure product was obtained by flash column chromatography on silica gel (petroleum: ethyl ether = 10:1).

General method for the condition optimization (Supplementary Tables 1-5)**.** A mixture of catalyst precursor, ligand, oxidant, *p*-tolylacetylene (**1a**) and cyclohexane (**2a**) was sealed in a Teflon septum screw-capped tube under N_2. The mixture was stirred in an oil bath at 130 ^{o}C for 3 hours. After cooling to room temperature, the reaction mixture was then analyzed by GC analysis with biphenyl as the internal standard.

Method of the radical trapping experiment. In an oven-dried Teflon septum screw-capped tube equipped with a stir bar, $Cu(OTf)_2$ (13.6 mg, 0.038 mmol), $Ni(acac)_2$ (9.6 mg, 0.0375 mmol), dppb (16.0 mg, 0.038 mmol) and AgOAc (8.3 mg, 0.050 mmol) were combined and sealed. The tube was then charged with nitrogen. Then **1a** (4.0 mL) and PhCl (3.0 mL) was injected into the tube by syringe. After stirring for 5 min, DTBP (146 mg, 1.5 mmol) and *p*-tolylacetylene **2a** (58.0 mg, 0.50 mmol) were subsequently injected into the reaction tube. The reaction was then heated to 130 ^{o}C. After stirring for 3 h, the reaction was cooled down to room temperature. The solvent of the reaction was filtered to obtain a crude mixture. The GC/MS (Gas chromatography-mass spectrometry) of the reaction mixture clearly showed the existence of cyclohexyl radical trapped by TEMPO (See Supplementary Fig. 66) and the yield of radical trapping product **7** was determined to be 80% by ^{1}H NMR analysis with CH_2Br_2 (70.0 mg) as the internal standard (See Supplementary Fig. 67).

Method of the kinetic isotope effect experiments. In oven-dried Teflon septum screw-capped tubes equipped with stir bar, $Cu(OTf)_2$ (6.8 mg, 0.019 mmol), $Ni(acac)_2$ (4.8 mg, 0.0198 mmol), dppb (8.0 mg, 0.019 mmol) and AgOAc (4.1 mg, 0.025 mmol) were combined and sealed. The tubes

were then charged with nitrogen. Then **1a** (2.0 mL) and PhCl (1.5 mL) were injected into the tubes by syringe. After stirring for 5 min, DTBP (109.5 mg, 0.75 mmol) and *p*-tolylacetylene **2a** (29.0 mg, 0.25 mmol) were subsequently injected into the reaction tubes. The reactions were then heated to 130 ^{o}C. The tubes were removed at different designated time interval (10, 20, 30, and 40 min). After cooling to room temperature, the reactions were then analyzed by GC analysis with biphenyl as the internal standard. By using above procedure, the similar sets of experiments were conducted by using [D_{12}]-**1a** instead of **1a** and using [D]-**2a** instead of **2a**. The KIE value for C(sp^3)-H bond cleavage of unactivated alkane was k_H/k_D = 2.2 and the KIE value for C(sp)-H bond cleavage of terminal alkyne was k_H/k_D = 0.9 (See Supplementary Fig. 68).

Analytical Data of Compounds

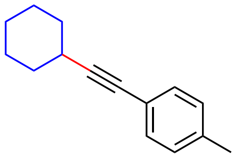

1-(Cyclohexylethynyl)-4-methylbenzene (3aa):[4] colorless oil was obtained in 73% isolated yield. ^{1}H NMR (400 MHz, $CDCl_3$) δ 7.28 (d, *J* = 8.1 Hz, 2H), 7.06 (d, *J* = 7.9 Hz, 2H), 2.56 (tt, *J* = 9.0, 3.6 Hz, 1H), 2.31 (s, 3H), 1.92 – 1.82 (m, 2H), 1.80 – 1.69 (m, 2H), 1.59 – 1.46 (m, 3H), 1.40 – 1.28 (m, 3H). ^{13}C NMR (101 MHz, $CDCl_3$) δ 137.26, 131.38, 128.84, 120.99, 93.58, 80.47, 32.75, 29.65, 25.92, 24.90, 21.34.

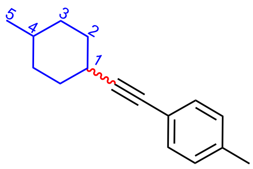

1-Methyl-4-((4-methylcyclohexyl)ethynyl)benzene (3ba): colorless oil was obtained in 70% isolated yield (a mixture of all regioisomers). ^{1}H NMR (400 MHz, Chloroform-*d*) δ 7.40 – 7.31 (m, 2H), 7.18 – 7.10 (m, 2H), 3.09 – 2.86 (m, 0.1H), 2.55 – 2.44 (m, 0.5H), 2.38 (s, 3H), 2.14 – 2.03 (m, 1.5H), 1.99 – 1.68 (m, 3H), 1.56 – 1.41 (m, 1.5H), 1.40 – 1.25 (m, 2.5H), 1.22 – 1.08 (m, 1H), 1.02 – 0.89 (m, 3H). ^{13}C NMR (101 MHz, $CDCl_3$) δ 137.30, 131.39, 128.86, 120.89, 99.93, 93.78, 93.16, 79.81, 41.67, 39.54, 39.51, 37.79, 37.75, 34.62, 34.54, 34.37, 33.23, 33.06, 32.78, 32.76, 32.30, 31.88, 31.08, 30.82, 30.27, 30.00, 29.70, 27.96, 25.80, 23.41, 22.57, 22.53, 21.96, 21.38. HRMS (ESI) calculated for $C_{16}H_{20}$ $[M+H]^+$: 213.1638; found: 213.1638.

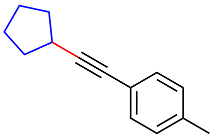

1-(Cyclopentylethynyl)-4-methylbenzene (3ca):[4] colorless oil was obtained in 52% isolated yield. ^{1}H NMR (400 MHz, $CDCl_3$) δ 7.28 (d, *J* = 8.0 Hz, 2H), 7.07 (d, *J* = 7.9 Hz, 2H), 2.80 (p, *J* = 7.5 Hz, 1H), 2.32 (s, 3H), 2.02 – 1.93 (m, 2H), 1.82 – 1.63 (m, 4H), 1.63 – 1.53 (m, 2H). ^{13}C NMR (101 MHz, $CDCl_3$) δ 137.27, 131.34, 131.30, 128.93, 128.86, 120.98, 93.71, 80.00, 33.92, 30.78, 25.01, 21.37.

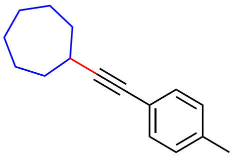

(*p*-Tolylethynyl)cycloheptane (3da): colorless oil was obtained in 59% isolated yield. A small amount of the direct addition product was observed in the spectra. ^{1}H NMR (400 MHz, Chloroform-*d*) δ 7.28 (d, *J* = 8.0 Hz, 2H), 7.07 (d, *J* = 7.8 Hz, 2H), 2.79 (tt, *J* = 7.7, 3.9 Hz, 1H), 2.32 (s, 3H), 1.95 – 1.85 (m, 2H), 1.81 – 1.70 (m, 4H), 1.63 – 1.48 (m, 6H). ^{13}C NMR (101 MHz, $CDCl_3$) δ 137.26, 131.34, 128.87, 121.08, 94.35, 80.73, 34.72, 31.68, 27.90, 25.62, 21.38. HRMS (ESI) calculated for $C_{16}H_{20}$ $[M+H]^+$: 213.1638; found: 213.1638.

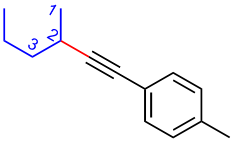

1-Methyl-4-(3-methylhex-1-yn-1-yl)benzene (3ea): colorless oil was obtained in 46% isolated yield (a mixture, C(1):C(2):C(3) =2:5:0, determined by GC-MS). ^{1}H NMR (400 MHz, $CDCl_3$) δ 7.28 (d, *J* = 8.0 Hz, 2H), 7.10 – 7.05 (m, 2H), 2.70 – 2.57 (m, 0.6H), 2.38 (t, *J* = 7.1 Hz, 0.8H), 2.32 (s, 3H), 1.64 – 1.40 (m, 4.5H), 1.40 – 1.30 (m, 0.9H), 1.25 – 1.22 (m, 1.9H), 1.05 (t, *J* = 7.4 Hz, 0.9H), 0.97 – 0.91 (m, 2.4H). ^{13}C NMR (101 MHz, $CDCl_3$) δ 137.37, 137.31, 131.39, 131.37, 128.90, 128.86, 120.97, 120.95, 93.98, 89.62, 81.85, 80.60, 39.23, 35.70, 31.13, 28.52, 27.80, 26.24, 22.25, 21.38, 21.14, 20.61, 19.38, 13.96, 11.92. HRMS (ESI) calculated for $C_{14}H_{18}$ $[M+H]^+$: 187.1481; found: 187.1482.

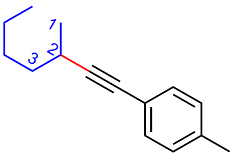

1-Methyl-4-(3-methylhept-1-yn-1-yl)benzene (3fa): colorless oil was obtained in 47% isolated yield (a mixture, C(1):C(2):C(3) =3:6:2, determined by GC-MS). ^{1}H NMR (400 MHz, $CDCl_3$) δ 7.28 (d, *J* = 8.1 Hz, 2H), 7.07 (d, *J* = 7.9 Hz, 2H), 2.67 – 2.56 (m, 0.5H), 2.51 – 2.43 (m, 0.2H), 2.38 (t, *J* = 7.1 Hz, 0.6H), 2.32 (s, 3H), 1.63 – 1.41 (m, 4.5H), 1.37 – 1.29 (m, 2.3H), 1.23 (d, *J* = 6.9 Hz, 1.8H), 1.05 (t, *J* = 7.4 Hz, 0.6H), 0.95 – 0.89 (m, 2.9H). ^{13}C NMR (101 MHz, $CDCl_3$) δ 137.34, 137.29, 137.24, 131.41, 131.40, 131.37, 128.89, 128.86, 121.13, 121.02, 121.00, 94.05, 92.79, 89.61, 81.74, 80.61, 80.53, 37.05, 36.78, 33.77, 31.38, 29.66, 28.79, 28.61, 28.21, 26.49, 22.57, 21.36, 21.15, 20.67, 19.42, 14.08, 14.06, 14.02, 11.88. HRMS (ESI) calculated for $C_{16}H_{20}$ $[M+H]^+$: 201.1638; found: 201.1639.

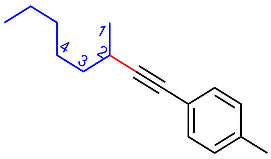

1-Methyl-4-(3-methyloct-1-yn-1-yl)benzene (3ga): colorless oil was obtained in 54% isolated yield (a mixture, C(1):C(2):C(3):C(4) =6:13:4:1, determined by GC-MS). ^{1}H NMR (400 MHz, $CDCl_3$) δ 7.31 – 7.26 (m, 2H), 7.07 (d, *J* = 7.7 Hz, 2H), 2.67 – 2.57 (m, 0.5H), 2.57 – 2.51 (m, 0.1H), 2.49 – 2.42 (m, 0.2H), 2.38 (t, *J* = 7.1 Hz, 0.6H), 2.32 (s, 3H), 1.64 – 1.40 (m, 5H), 1.36 – 1.28 (m, 4H), 1.27 – 1.21 (m, 2H), 1.05 (t, *J* = 7.4 Hz, 0.6H), 0.97 – 0.85 (m, 3H). ^{13}C NMR (101 MHz, $CDCl_3$) δ 137.34, 137.28, 137.24, 131.40, 131.39, 131.36, 131.31, 128.93, 128.89, 128.85, 121.12, 121.00, 120.97, 94.04, 92.97, 92.86, 89.61, 81.71, 81.57, 80.61, 80.51, 37.45, 37.02, 34.57, 33.99, 31.85, 31.76, 31.69, 29.72, 28.89, 28.85, 28.83, 28.20, 27.11, 26.51, 21.37, 21.15, 20.65, 19.41, 14.08, 11.89. HRMS (ESI) calculated for $C_{16}H_{22}$ $[M+H]^+$: 215.1794; found: 215.1793.

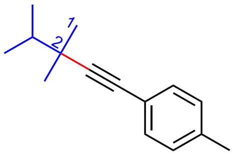

1-Methyl-4-(3,3,4-trimethylpent-1-yn-1-yl)benzene (3ha): colorless oil was obtained in 45% isolated yield (a mixture, C(1):C(2) =1:3, determined by GC-MS). Spectra of the isomer at the C(2) position was given. ^{1}H NMR (400 MHz, $CDCl_3$) δ 7.28 (d, *J* = 8.1 Hz, 2H), 7.07 (d, *J* = 7.9 Hz, 2H), 2.32 (s, 3H), 1.62 (p, *J* = 6.8 Hz, 1H), 1.24 (s, 6H), 1.02 (d, *J* = 6.8 Hz, 6H). ^{13}C NMR (101 MHz, $CDCl_3$) δ 137.18, 131.39, 128.82, 121.15, 95.99, 80.81, 37.83, 35.43, 27.00, 21.37, 18.39. HRMS (ESI) calculated for $C_{16}H_{20}$ $[M+H]^+$: 201.1638; found: 201.1639.

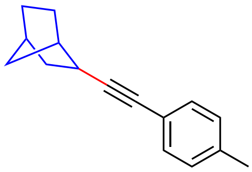

2-(*p*-Tolylethynyl)bicyclo[2.2.1]heptane (3ia):[5] colorless oil was obtained in 49% isolated yield. ^{1}H NMR (400 MHz, Chloroform-*d*) δ 7.26 (d, *J* = 8.1 Hz, 2H), 7.07 (d, *J* = 7.7 Hz, 2H), 2.48 – 2.42 (m, 1H), 2.42 – 2.37 (m, 1H), 2.34 – 2.29 (m, 4H), 1.74 – 1.61 (m, 3H), 1.56 – 1.48 (m, 2H), 1.27 – 1.15 (m, 3H). ^{13}C NMR (101 MHz, $CDCl_3$) δ 137.22, 131.30, 128.86, 121.09, 94.83, 80.10, 43.67, 39.39, 36.65, 36.19, 33.56, 28.82, 28.80, 21.37.

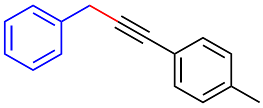

2-(Cyclohexylethynyl)thiophene (3ja):[6] colorless oil was obtained in 74% isolated yield. ^{1}H NMR (400 MHz, Chloroform-*d*) δ 7.42 (d, *J* = 7.6 Hz, 2H), 7.38 – 7.30 (m, 4H), 7.27 – 7.22 (m, 1H), 7.10 (d, *J* = 7.8 Hz, 2H), 3.83 (s, 2H), 2.34 (s, 3H). ^{13}C NMR (101 MHz, $CDCl_3$) δ 137.80, 136.85, 131.47, 128.96, 128.49, 127.92, 126.55, 120.51, 86.66, 82.66, 25.72, 21.42.

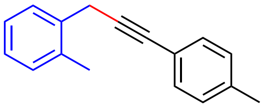

1-Methyl-2-(3-(p-tolyl)prop-2-yn-1-yl)benzene (3ka): colorless oil was obtained in 78% isolated yield. ^{1}H NMR (400 MHz, Chloroform-*d*) δ 7.55 – 7.48 (m, 1H), 7.33 (d, *J* = 7.8 Hz, 2H), 7.23 – 7.15 (m, 3H), 7.10 (d, *J* = 7.7 Hz, 2H), 3.74 (s, 2H), 2.37 (s, 3H), 2.34 (s, 3H). ^{13}C NMR (101 MHz, $CDCl_3$) δ 137.75, 136.01, 135.09, 131.44, 130.03, 128.94, 128.28, 126.80, 126.15, 120.59, 86.35, 82.78, 23.92, 21.42, 19.35. HRMS (EI) calculated for $C_{17}H_{16}$ $[M]^+$: 220.1252; found: 220.1258.

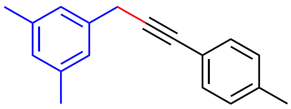

1,3-Dimethyl-5-(3-(p-tolyl)prop-2-yn-1-yl)benzene (3la): colorless oil was obtained in 80% isolated yield. ^{1}H NMR (400 MHz, $CDCl_3$) δ 7.34 (d, *J* = 8.1 Hz, 2H), 7.10 (d, *J* = 7.8 Hz, 2H), 7.02 (d, *J* = 1.7 Hz, 2H), 6.88 (s, 1H), 3.75 (s, 2H), 2.34 (s, 3H), 2.31 (s, 6H). ^{13}C NMR (101 MHz, $CDCl_3$) δ 138.06, 137.71, 136.71, 131.50, 128.93, 128.19, 125.77, 120.67, 87.01, 82.36, 25.54, 21.41, 21.27. HRMS (EI) calculated for $C_{18}H_{18}$ $[M]^+$: 234.1409; found: 234.1413.

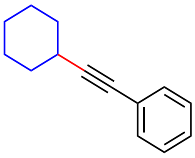

(Cyclohexylethynyl)benzene (3ab):[4] colorless oil was obtained in 66% isolated yield. ^{1}H NMR (400 MHz, $CDCl_3$) δ 7.42 – 7.36 (m, 2H), 7.29 – 7.22 (m, 3H), 2.58 (tt, J = 9.0, 3.6 Hz, 1H), 1.94 – 1.83 (m, 2H), 1.81 – 1.69 (m, 2H), 1.60 – 1.47 (m, 3H), 1.41 – 1.27 (m, 3H). ^{13}C NMR (101 MHz, $CDCl_3$) δ 131.52, 128.09, 127.35, 124.08, 94.40, 80.47, 32.68, 29.63, 25.90, 24.88.

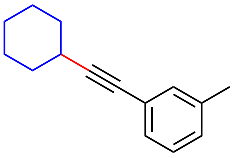

1-(Cyclohexylethynyl)-3-methylbenzene (3ac): colorless oil was obtained in 73% isolated yield. ^{1}H NMR (400 MHz, $CDCl_3$) δ 7.24 – 7.17 (m, 2H), 7.15 (t, J = 7.5 Hz, 1H), 7.05 (d, J = 7.4 Hz, 1H), 2.57 (tt, J = 9.0, 3.6 Hz, 1H), 2.30 (s, 3H), 1.92 – 1.82 (m, 2H), 1.80 – 1.70 (m, 2H), 1.59 – 1.46 (m, 3H), 1.40 – 1.28 (m, 3H). ^{13}C NMR (101 MHz, $CDCl_3$) δ 137.69, 132.15, 128.55, 128.23, 127.99, 123.86, 94.01, 80.59, 32.71, 29.62, 25.91, 24.87, 21.15. HRMS (ESI) calculated for $C_{15}H_{18}$ $[M+H]^+$: 199.1481; found: 199.1482.

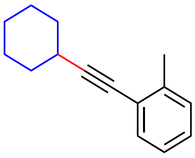

1-(Cyclohexylethynyl)-2-methylbenzene (3ad): colorless oil was obtained in 76% isolated yield. ^{1}H NMR (400 MHz, $CDCl_3$) δ 7.36 (d, J = 7.2 Hz, 1H), 7.18 – 7.11 (m, 2H), 7.11 – 7.05 (m, 1H), 2.64 (tt, J = 8.6, 3.6 Hz, 1H), 2.41 (s, 3H), 1.93 – 1.82 (m, 2H), 1.81 – 1.71 (m, 2H), 1.63 – 1.47 (m, 3H), 1.43 – 1.31 (m, 3H). ^{13}C NMR (101 MHz, $CDCl_3$) δ 139.87, 131.64, 129.19, 127.34, 125.34, 123.82, 98.53, 79.37, 32.79, 29.73, 25.96, 24.77, 20.74. HRMS (ESI) calculated for $C_{15}H_{18}$ $[M+H]^+$: 199.1481; found: 199.1482.

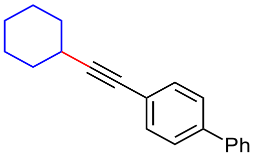

4-(Cyclohexylethynyl)-1,1'-biphenyl (3ae):[4] white solid was obtained in 74% isolated yield. ^{1}H NMR (400 MHz, $CDCl_3$) δ 7.60 – 7.55 (m, 2H), 7.54 – 7.48 (m, 2H), 7.48 – 7.39 (m, 4H), 7.36 – 7.30 (m, 1H), 2.60 (tt, J = 9.0, 3.6 Hz, 1H), 1.97 – 1.83 (m, 2H), 1.83 – 1.69 (m, 2H), 1.61 – 1.49

(m, 3H), 1.41 – 1.30 (m, 3H). ^{13}C NMR (101 MHz, $CDCl_3$) δ 140.50, 140.07, 131.94, 128.77, 127.39, 126.93, 126.80, 123.07, 95.18, 80.33, 32.71, 29.72, 25.92, 24.91.

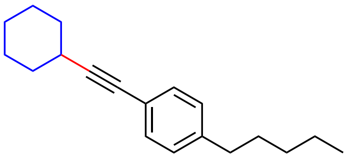

1-(Cyclohexylethynyl)-4-pentylbenzene (3af): colorless oil was obtained in 83% isolated yield. ^{1}H NMR (400 MHz, $CDCl_3$) δ 7.30 (d, J = 8.0 Hz, 2H), 7.08 (d, J = 8.0 Hz, 2H), 2.61 – 2.51 (m, 3H), 1.94 – 1.81 (m, 2H), 1.80 – 1.69 (m, 2H), 1.63 – 1.46 (m, 5H), 1.39 – 1.23 (m, 7H), 0.87 (t, J = 6.8 Hz, 3H). ^{13}C NMR (101 MHz, $CDCl_3$) δ 142.36, 131.39, 131.31, 128.27, 128.23, 121.17, 93.62, 80.50, 35.75, 32.76, 31.38, 30.97, 29.64, 25.93, 24.90, 22.51, 14.01. HRMS (ESI) calculated for $C_{19}H_{26}$ $[M+H]^+$: 255.2107; found: 255.2107.

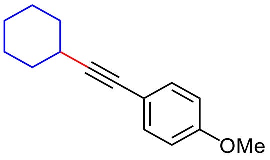

1-(Cyclohexylethynyl)-4-methoxybenzene (3ag):[4] colorless oil was obtained in 91% isolated yield. ^{1}H NMR (400 MHz, $CDCl_3$) δ 7.33 (d, J = 8.6 Hz, 2H), 6.80 (d, J = 8.6 Hz, 2H), 3.79 (s, 3H), 2.63 – 2.48 (m, 1H), 1.94 – 1.82 (m, 2H), 1.82 – 1.69 (m, 2H), 1.58 – 1.44 (m, 3H), 1.40 – 1.27 (m, 3H). ^{13}C NMR (101 MHz, $CDCl_3$) δ 158.87, 132.84, 116.23, 113.70, 92.82, 80.09, 55.21, 32.81, 29.65, 25.92, 24.94.

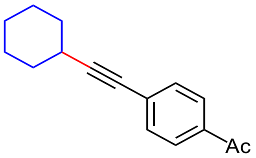

1-(4-(Cyclohexylethynyl)phenyl)ethan-1-one (3ah):[7] colorless oil was obtained in 50% isolated yield. A small amount of the direct addition product was observed in the spectra. ^{1}H NMR (400 MHz, Chloroform-*d*) δ 7.87 (d, J = 8.4 Hz, 2H), 7.46 (d, J = 8.4 Hz, 2H), 2.66 – 2.60 (m, 1H), 2.59 (s, 3H), 1.94 – 1.85 (m, 2H), 1.81 – 1.71 (m, 2H), 1.61 – 1.46 (m, 3H), 1.41 – 1.30 (m, 3H). ^{13}C NMR (101 MHz, $CDCl_3$) δ 197.44, 135.51, 131.63, 129.18, 128.33, 128.10, 98.33, 32.46, 29.73, 26.58, 25.81, 24.84.

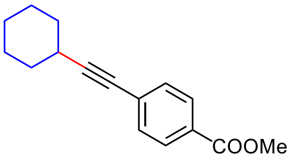

Methyl 4-(cyclohexylethynyl)benzoate (3ai):[8] colorless oil was obtained in 65% isolated yield. A small amount of the direct addition product was observed in the spectra. ^{1}H NMR (400 MHz, Chloroform-*d*) δ 7.95 (d, *J* = 8.5 Hz, 2H), 7.44 (d, *J* = 8.4 Hz, 2H), 3.90 (s, 3H), 2.60 (tt, *J* = 8.8, 3.5 Hz, 1H), 1.94 – 1.82 (m, 2H), 1.82 – 1.68 (m, 2H), 1.61 – 1.48 (m, 3H), 1.41 – 1.30 (m, 3H). ^{13}C NMR (101 MHz, $CDCl_3$) δ 166.64, 131.44, 129.30, 128.95, 128.66, 97.88, 52.09, 32.47, 29.70, 25.82, 24.83.

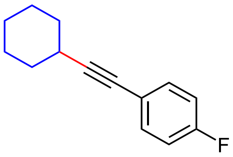

1-(Cyclohexylethynyl)-4-fluorobenzene (3aj):[4] colorless oil was obtained in 61% isolated yield. ^{1}H NMR (400 MHz, $CDCl_3$) δ 7.39 – 7.33 (m, 2H), 7.00 – 6.91 (m, 2H), 2.56 (tt, *J* = 9.0, 3.6 Hz, 1H), 1.93 – 1.81 (m, 2H), 1.81 – 1.69 (m, 2H), 1.57 – 1.45 (m, 3H), 1.40 – 1.29 (m, 3H). ^{13}C NMR (101 MHz, $CDCl_3$) δ 161.96 (d, J_{C-F} = 248.8 Hz), 133.29 (d, J_{C-F} = 8.2 Hz), 120.14 (d, J_{C-F} = 3.4 Hz), 115.29 (d, J_{C-F} = 22.0 Hz), 94.05, 79.39, 32.66, 29.59, 25.88, 24.90. ^{19}F NMR (377 MHz, $CDCl_3$) δ -112.49.

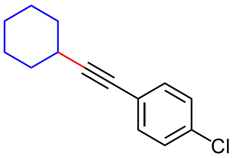

1-Chloro-4-(cyclohexylethynyl)benzene (3ak):[9] colorless oil was obtained in 78% isolated yield. ^{1}H NMR (400 MHz, $CDCl_3$) δ 7.31 (d, *J* = 8.5 Hz, 2H), 7.23 (d, *J* = 8.5 Hz, 2H), 2.56 (tt, *J* = 8.8, 3.5 Hz, 1H), 1.91 – 1.81 (m, 2H), 1.80 – 1.69 (m, 2H), 1.60 – 1.46 (m, 3H), 1.40 – 1.28 (m, 3H). ^{13}C NMR (101 MHz, $CDCl_3$) δ 133.25, 132.75, 128.39, 122.59, 95.46, 79.42, 32.57, 29.62, 25.85, 24.87.

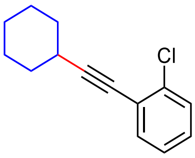

1-Chloro-2-(cyclohexylethynyl)benzene (3al): colorless oil was obtained in 71% isolated yield. ^{1}H NMR (400 MHz, $CDCl_3$) δ 7.45 – 7.40 (m, 1H), 7.38 – 7.33 (m, 1H), 7.20 – 7.12 (m, 2H), 2.67 (tt, *J* = 8.4, 3.6 Hz, 1H), 1.93 – 1.83 (m, 2H), 1.83 – 1.73 (m, 2H), 1.64 – 1.48 (m, 3H), 1.44 – 1.31 (m, 3H). ^{13}C NMR (101 MHz, $CDCl_3$) δ 135.78, 133.14, 129.04, 128.36, 126.22, 123.87, 100.08, 77.55, 32.43, 29.70, 25.93, 24.64. HRMS (ESI) calculated for $C_{14}H_{15}Cl$ $[M+H]^+$: 219.0935; found:

219.0934.

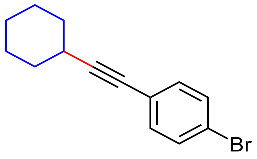

1-Bromo-4-(cyclohexylethynyl)benzene (3am):[4] colorless oil was obtained in 65% isolated yield. ^{1}H NMR (400 MHz, $CDCl_3$) δ 7.40 (d, *J* = 8.5 Hz, 2H), 7.25 (d, *J* = 8.4 Hz, 2H), 2.56 (tt, *J* = 8.9, 3.5 Hz, 1H), 1.92 – 1.82 (m, 2H), 1.80 – 1.69 (m, 2H), 1.59 – 1.46 (m, 3H), 1.40 – 1.28 (m, 3H). ^{13}C NMR (101 MHz, $CDCl_3$) δ 133.01, 131.31, 123.06, 121.41, 95.69, 79.48, 32.54, 29.65, 25.85, 24.87.

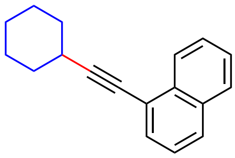

2-(Cyclohexylethynyl)naphthalene (3an):[10] colorless oil was obtained in 66% isolated yield. ^{1}H NMR (400 MHz, $CDCl_3$) δ 8.34 (d, *J* = 8.2 Hz, 1H), 7.82 (d, *J* = 7.9 Hz, 1H), 7.76 (d, *J* = 8.2 Hz, 1H), 7.62 (d, *J* = 6.6 Hz, 1H), 7.58 – 7.52 (m, 1H), 7.52 – 7.45 (m, 1H), 7.42 – 7.35 (m, 1H), 2.75 (tt, *J* = 8.3, 3.2 Hz, 1H), 2.05 – 1.94 (m, 2H), 1.89 – 1.77 (m, 2H), 1.72 – 1.56 (m, 3H), 1.46 – 1.36 (m, 3H). ^{13}C NMR (101 MHz, $CDCl_3$) δ 133.45, 133.15, 129.88, 128.14, 127.79, 126.42, 126.28, 126.14, 125.19, 121.74, 99.60, 78.45, 32.84, 29.96, 25.95, 24.93.

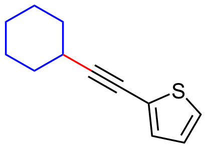

2-(Cyclohexylethynyl)thiophene (3ao):[11] colorless oil was obtained in 69% isolated yield. ^{1}H NMR (400 MHz, Chloroform-*d*) δ 7.16 (d, *J* = 5.2 Hz, 1H), 7.11 (d, *J* = 3.6 Hz, 1H), 6.93 (dd, *J* = 5.2, 3.6 Hz, 1H), 2.59 (tt, *J* = 9.2, 3.8 Hz, 1H), 1.94 – 1.82 (m, 2H), 1.80 – 1.69 (m, 2H), 1.58 – 1.48 (m, 3H), 1.39 – 1.29 (m, 3H). ^{13}C NMR (101 MHz, $CDCl_3$) δ 130.82, 126.71, 125.77, 124.21, 98.37, 73.48, 32.46, 29.89, 25.83, 24.89.

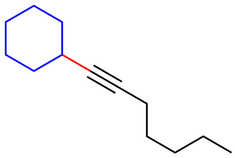

Hept-1-yn-1-ylcyclohexane (3ap):[12] colorless oil was obtained in 63% isolated yield. ^{1}H NMR

(400 MHz, $CDCl_3$) δ 2.36 – 2.27 (m, 1H), 2.15 (td, *J* = 7.1, 2.2 Hz, 2H), 1.81 – 1.73 (m, 2H), 1.73 – 1.65 (m, 2H), 1.54 – 1.44 (m, 3H), 1.42 – 1.23 (m, 9H), 0.90 (t, *J* = 7.0 Hz, 3H). ^{13}C NMR (101 MHz, $CDCl_3$) δ 84.57, 80.06, 33.18, 31.04, 29.15, 28.93, 25.95, 24.95, 22.22, 18.70, 14.01.

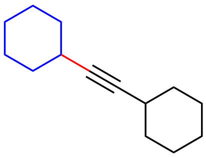

1,2-Dicyclohexylethyne (3aq):[13] colorless oil was obtained in 86% isolated yield. ^{1}H NMR (400 MHz, $CDCl_3$) δ 2.33 (td, *J* = 9.0, 4.5 Hz, 2H), 1.82 – 1.65 (m, 8H), 1.53 – 1.45 (m, 2H), 1.45 – 1.34 (m, 4H), 1.33 – 1.24 (m, 6H). ^{13}C NMR (101 MHz, $CDCl_3$) δ 84.48, 33.20, 29.02, 25.98, 24.84.

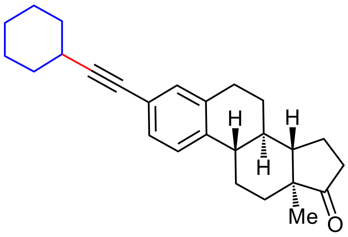

3-Cyclohexylethynylestrone (3ar): white solid was obtained in 70% isolated yield. ^{1}H NMR (400 MHz, Chloroform-*d*) δ 7.22 – 7.12 (m, 3H), 2.87 (dd, *J* = 8.8, 4.0 Hz, 2H), 2.62 – 2.45 (m, 2H), 2.45 – 2.36 (m, 1H), 2.33 – 2.24 (m, 1H), 2.21 – 2.11 (m, 1H), 2.10 – 1.93 (m, 3H), 1.92 – 1.82 (m, 2H), 1.81 – 1.69 (m, 2H), 1.68 – 1.45 (m, 9H), 1.39 – 1.28 (m, 3H), 0.91 (s, 3H). ^{13}C NMR (101 MHz, $CDCl_3$) δ 220.87, 139.18, 136.29, 131.97, 128.82, 125.15, 121.39, 93.71, 80.36, 50.43, 47.93, 44.34, 37.97, 35.82, 32.73, 31.50, 29.62, 29.05, 26.35, 25.91, 25.57, 24.87, 21.54, 13.81. HRMS (ESI) calculated for $C_{26}H_{32}O$ $[M+H]^+$: 361.2526; found: 361.2526.

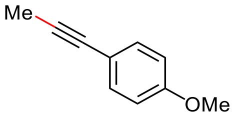

1-Methoxy-4-(prop-1-yn-1-yl)benzene (6):[14] pale yellow oil was obtained in 59% isolated yield. ^{1}H NMR (400 MHz, Chloroform-*d*) δ 7.32 (d, *J* = 8.7 Hz, 2H), 6.81 (d, *J* = 8.7 Hz, 2H), 3.79 (s, 3H), 2.03 (s, 3H). ^{13}C NMR (101 MHz, $CDCl_3$) δ 158.92, 132.75, 116.09, 113.76, 84.09, 79.37, 55.20, 4.29.

Supplementary References

1. Zhang Z, Jiang X. Oxidative Coupling of Terminal Alkyne with α-Hydroxy Ketone: An Expedient Approach toward Ynediones. *Org Lett* **16**, 4400-4403 (2014).
2. Osakada K, Sakata R, Yamamoto T. Preparation and Properties of trans-Pd(Ar)(C⋮CPh)(PEt3)2. Intermolecular Alkynyl Ligand Transfer between Copper(I) and Palladium(II) Complexes Relevant to Palladium Complex Catalyzed Cross-Coupling of Terminal Alkyne with Haloarene in the Presence of CuI Cocatalyst. *Organometallics* **16**, 5354-5364 (1997).
3. Vitérisi A, Orsini A, Weibel J-M, Pale P. A mild access to silver acetylides from trimethylsilyl acetylenes. *Tetrahedron Lett* **47**, 2779-2781 (2006).
4. Liu W, Li L, Li C-J. Empowering a transition-metal-free coupling between alkyne and alkyl iodide with light in water. *Nat Commun* **6:6526**, doi: 10.1038/ncomms7526 (2015).
5. Kohno K, Nakagawa K, Yahagi T, Choi J-C, Yasuda H, Sakakura T. Fe(OTf)3-Catalyzed Addition of sp C−H Bonds to Olefins. *J Am Chem Soc* **131**, 2784-2785 (2009).
6. Qian M, Negishi E-i. Palladium-catalyzed cross-coupling reaction of alkynylzincs with benzylic electrophiles. *Tetrahedron Lett* **46**, 2927-2930 (2005).
7. Rao MLN, Jadhav DN, Dasgupta P. Pd-Catalyzed Domino Synthesis of Internal Alkynes Using Triarylbismuths as Multicoupling Organometallic Nucleophiles. *Org Lett* **12**, 2048-2051 (2010).
8. Semba K, Fujihara T, Terao J, Tsuji Y. Copper-Catalyzed Highly Regio- and Stereoselective Directed Hydroboration of Unsymmetrical Internal Alkynes: Controlling Regioselectivity by Choice of Catalytic Species. *Chem –Eur J* **18**, 4179-4184 (2012).
9. Gil-Moltó J, Nájera C. Palladium(II) Chloride and a (Dipyridin-2-ylmethyl)amine-Derived Palladium(II) Chloride Complex as Highly Efficient Catalysts for the Synthesis of Alkynes in Water or in NMP and of Diynes in the Absence of Reoxidant. *Eur J Org Chem* **2005**, 4073-4081 (2005).
10. Cívicos JF, Alonso DA, Nájera C. Microwave-Promoted Copper-Free Sonogashira–Hagihara Couplings of Aryl Imidazolylsulfonates in Water. *Adv Synth Catal* **355**, 203-208 (2013).
11. Fleckenstein CA, Plenio H. Aqueous/organic cross coupling: Sustainable protocol for Sonogashira reactions of heterocycles. *Green Chem* **10**, 563-570 (2008).
12. Gerard J, Hevesi L. Transformation of β-chalcogeno alkenylboranes into tetrasubstituted olefins. *Tetrahedron* **60**, 367-381 (2004).
13. Malanga C, Aronica LA, Lardicci L. Nickel mediated double bond formation from vic-dibromides and ethyl magnesium bromide. *Tetrahedron Lett* **36**, 9189-9192 (1995).
14. Zhang W, Kraft S, Moore JS. Highly Active Trialkoxymolybdenum(VI) Alkylidyne Catalysts Synthesized by a Reductive Recycle Strategy. *J Am Chem Soc* **126**, 329-335 (2004).
